# Operationalising routinely collected patient data in research to further the pursuit of social justice and health equity: a team-based scoping review

**DOI:** 10.1186/s12874-025-02466-9

**Published:** 2025-01-21

**Authors:** Katie Chadd, Anna Caute, Anna Pettican, Pam Enderby

**Affiliations:** 1 University of Essex, Colchester, Essex UK; 2 University of Sheffield, Sheffield, UK

**Keywords:** Routinely collected data, Real world data, Big data, Social justice, Health inequalities, Allied health professions

## Abstract

**Background:**

Vast volumes of routinely collected data (RCD) about patients are collated by health professionals. Leveraging this data – a form of real-world data - can be valuable for quality improvement and contributing to the evidence-base to inform practice. Examining routine data may be especially useful for examining issues related to social justice such as health inequities. However, little is known about the extent to which RCD is utilised in health fields and published for wider dissemination.

**Objectives:**

The objective of this scoping review is to document the peer-reviewed published research in allied health fields which utilise RCD and evaluate the extent to which these studies have addressed issues pertaining to social justice.

**Methods:**

An enhanced version of the Arksey and O’Malley’s framework, put forth by Westphalm et al. guided the scoping review. A comprehensive literature search of three databases identified 1584 articles. Application of inclusion and exclusion criteria was piloted on 5% of the papers by three researchers. All titles and abstracts were screened independently by 2 team members, as were full texts. A data charting framework, developed to address the research questions, was piloted by three researchers with data extraction being completed by the lead researcher. A sample of papers were independently charted by a second researcher for reliability checking.

**Results:**

One hundred and ninety papers were included in the review. The literature was diverse in terms of the professions that were represented: physiotherapy (33.7%) and psychology/mental health professions (15.8%) predominated. Many studies were first authored by clinicians (44.2%), often with clinical-academic teams. Some (33.25%) directly referenced the use of their studies to examine translation of research to practice. Few studies (14.2%) specifically tackled issues pertaining to social justice, though many collected variables that could have been utilised for this purpose.

**Conclusion:**

Studies operationalising RCD can meaningfully address research to practice gaps and provide new evidence about issues related to social justice. However, RCD is underutilised for these purposes. Given that vast volumes of relevant data are routinely collected, more needs to be done to leverage it, which would be supported by greater acknowledgement of the value of RCD studies.

**Supplementary Information:**

The online version contains supplementary material available at 10.1186/s12874-025-02466-9.

## Introduction

The contemporary capabilities of digital health infrastructures create enormous potential for scrutinising health care practices and treatments by analysing real-time, real-world data (RWD) to create real-world evidence (RWE) [1]. RWD most typically refers to information collected outside of traditional research studies, with RWE coined to refer to the evidence produced from its analysis [1]. Methodological approaches which tap into RWD sources offer an alternative understanding of ‘what works’ in practice, which can supplement evidence obtained through traditional approaches such as clinical trials [2]. The potential of analysing health data obtained outside of trials has been recognised by major stakeholders globally including the World Health Organization [3], the Food and Drug Administration [4] as well as the National Institute of Health and Care Excellence [5] in the United Kingdom. Nevertheless, RWD and RWE research is not without risks or challenges, and its observational nature, through the traditional lens of the research hierarchy in mainstream health and medicine research, often means it is sidelined and critiqued in favour of the rigour provided within a clinical trial [6].

Routinely collected data (RCD) describes data about patients that is documented routinely in clinical practice, typically in electronic medical records and registries [7], and is a vast source of RWD. Historically, RCD been associated with either being used by insurance providers using claims databases to make decisions about treatment options they will or will not cover [8] or individual care providers’ to run local service evaluations or audits, to drive quality improvement [9]. Such investigations therefore may not always be reported as *research*, be recognised with any value beyond their immediate means or have the chance to be published. This means their findings traditionally lack visibility, although the scholarly tide is beginning to change. Notably, through the COVID-19 pandemic, RCD was fundamental in the production of timely evidence regarding symptoms and treatment effects across sizeable cohorts which could inform public health policy [10]. RWD studies, including those based on RCD, are increasingly observed in the literature, alongside the acceleration of artificial-intelligence (AI) methods of analysis, and are being utilised as an opportunity to provide methodological enhancements, or substitutions in clinical trials [11–13].

Conducting analyses using RCD may also offer practising clinicians opportunities to interrogate their practice and lead research that has direct relevance to their daily services and address pertinent research-to-practice gaps. While recent shifts have been seen, in the UK especially, to value clinicians’ priorities for what health research is needed [14], challenges remain for non-medical and non-nursing staff (referred to herein as *allied health professionals)* to actually carry it out [15–17]. By minimising time and resource constraints through conducting analyses of already-collected data, available at a clinician’s fingertips, it is possible that clinician-led research may be maximised. This offers allied health professionals - who are often disadvantaged in their capacity to conduct research compared with medics and nurses [16, 18] - an opportunity to engage in research. However, the extent to which RWD studies using RCD in allied health fields are being conducted is unknown.

Beyond this, RWD studies using RCD can expose, and monitor changes in, social injustices created by healthcare systems, and are well placed for “*asking tough research questions that focus on dismantling racism*” [19, p. 724]. That is to say that harnessing patient data through RCD analysis can investigate inequities and disparities in access to and outcomes from healthcare. Moreover, such evidence can be used to ask *why* they exist and tackle pertinent barriers to social justice, which describes “*full participation in society… resulting in equitable living and a just ordering of society*” [20, p.955]. When good quality data is routinely collected about the social identities, social strata and/or likely marginalisation of an individual within their given context, alongside information about their health care access and outcomes, rich analyses exploring interactions of and equity across societal factors and health are possible [21, 22]. Taking a social justice lens to leveraging RCD also offers an alternative avenue to knowledge production which partly address the valid and historical reluctance of marginalised populations to participate in traditional research studies [23, 24]. It should however be noted that by the nature of the data, samples would be restricted only to individuals already accessing healthcare, thus RCD analyses are prone to risk of “data absenteeism” [25]. People such as those who are living without a fixed address, or gypsy, traveller and Roma communities may be especially vulnerable to this, and *absenteeism* should be attended to in any study. Furthermore, the value of RCD is inextricably dependent on good quality data that is consistently collected, specific to the cause, and valid and reliable, which can often be a challenge [26] .

Studies utilising RCD have several advantages over conventional trials. Firstly, they can utilise non-selected samples, meaning that the whole population receiving healthcare can be included and studied. This signals a major departure from the problematic consequences of conventional research approaches which historically fail to recruit diverse samples from their populations (even when the populations are highly diverse), as mentioned previously [27–30]. Fundamentally, this has resulted in the production of an evidence-base for clinical practice that is skewed towards benefiting the already-privileged population (notably, White people living in Western contexts) and thus represents one factor (though there are many, see Powell et al. for discussion in the UK [31]) that contributes to systemic injustices and inequities in health. A second advantage of using RCD to improve the evidence-base is there is an enhanced capacity for intersectional analyses through the systematic collection and linkage of large-scale patient data. This describes the capability of interrogating data using multiple levels to explore differences and associations between health metrics and compounding levels of disadvantage based on one’s multiple intersecting identities [32, 33]. Whilst the potential of intersectional analysis to help address issues such as health inequalities is noted [34], the extent to which this is applied to RCD in the allied health fields is not known.

A search on PROSPERO and the JBI systematic review register for ongoing and intended systematic reviews on the topic was conducted, which identified there were no similar reviews exploring the key components of allied heath, RCD and social justice. A literature search of key databases similarly indicated no other literature reviews which address the nexus of these topics. However, we did find several examples of scoping and systematic reviews exploring RWD for specific diseases related to allied health [35–37] and literature exploring social justice and allied health [38, 39]. But, we did not find research bridging these aspects to form a methodological perspective across professions. Existing reviews that bring together RWD and social justice are infrequent, and are limited to a clinical area (for example, on emergency care [40] or psychiatry [41]). An exception to this is a recent scoping review by Moorthrie et al. [21] who analysed RWD studies exploring health inequalities from diverse clinical areas. However their scoping exercise was relatively narrow, by including only studies which explicitly referred to improvements to data quality in relation to health inequalities. Therefore, to date, there is no comprehensive review of RWD studies in a broader sense that seeks to explicate their relevance to social justice, in order to understand the potential of operationalising data in this way.

To develop a greater understanding of the potential for RWD and RCD studies to foster this aspiration, it is helpful to conduct a scoping review to ascertain and map what already exists in the literature [42]. This scoping review signals as a departure point from the current rhetoric regarding health research and RWD on two fronts:

1. By validating RCD studies as sources of valuable evidence that can contribute to various health fields.
2. By positioning RCD studies as a fundamental approach for interrogating and dismantling injustices in healthcare treatments and services.

Drawing on the transformative research paradigm, guided by the transformative checklist [43], we outline a scoping review which aimed to:

- Document the use of RCD research in allied health fields in the published peer-reviewed literature.
- Describe the researcher team (clinician or academic), aims of studies, data sources and methods used in these studies.
- Evaluate the extent to which RCD research has aimed to address issues pertaining to social justice, specifically to document reference to:
  - ◦ tackling issues of health inequity.
  - ◦ inclusion of typically underserved populations in their samples.
  - ◦ intersectional analyses.
- Use the findings to create recommendations for future real-world data studies to advance the evidence base and particularly further the pursuit of social justice in health.

## Methods

A draft a priori protocol was uploaded onto the Open Science Framework (OSF) platform for the purposes of a consultation period [44]. The consultation was open from 02 November to 07 December 2023, and participation was encouraged through social media and via the researchers’ personal contacts. Comments and feedback were invited to be directly shared with the lead author, and the protocol was revised accordingly. The full protocol was published in 2024 [45] and is summarised here for convenience. Arksey and O’Malley’s methodological framework [46] was utilised to guide this scoping review, which also incorporated recommendations from Westphalm et al. to ‘enhance’ this process and utilise a team-based approach [47]. Operationalising ‘team-based’ meant developing and applying a framework to aid the selection and invitation of collaborators based on specific clinical and research expertise “into the method” (p5) [47], as reported in our protocol [45]. Whilst guided methodologically by these, our review is reported in line with the PRISMA Extension for Scoping Reviews (PRISMA-ScR) guidance [48].

### Stage 1) Specify the research question

The Sample, Phenomenon of Interest, Design, Evaluation, Research type (‘SPIDER’) framework guided the development of the research questions which explore qualitative and quantitative aspects of studies [49, 50], leading to the identification of the following components:

- Sample: Peer-reviewed studies that have used RCD in non-medial, non-nursing health (*allied health*) fields.
- Phenomenon of Interest: How RCD has been used, by whom, and whether it addresses issues related to social justice (health inequities, inequalities, inclusive sampling and intersectional analysis).
- Design: studies of any design using RCD besides those utilising data collected as part of a clinical trial (i.e. outside routine care).
- Evaluation: The volume of research published and the extent to which it addresses our phenomena of interest.
- Research type: Primary research adopting any methodology.

The research question guiding this review is: “What research has been published in allied health fields that utilises RCD as its primary method, and to what extent does it address key issues pertaining to social justice?”

### Stage 2) Identify the relevant literature

#### Eligibility criteria

Guided by the SPIDER framework, the eligibility criteria were that studies were peer-reviewed full-text papers related to allied health fields published at any time, and available in a language accessible to the research team (limited to English) (S-sample). This was to ensure maximal coverage, but limit results with insufficient data to evaluate to meet the aims of the review (such as conference proceedings which may not include detail on data sources, for example). Eligible studies were those demonstrating application of RCD (PI- phenomenon of interest), of any type of primary research (R-research) excluding studies utilising RCD collected through clinical trials (D-design).

#### Information sources

Searches were conducted in three health and medical electronic databases (MEDLINE Ultimate, CINAHL Ultimate and PubMed) which are presented and rationalised in the protocol. No other strategy was adopted (e.g. citation chasing or snowballing) nor was grey literature searched due to the volume and breadth of retrieved articles in trial searches [45]. CINAHL Ultimate and MEDLINE Ultimate databases were harnessed via using EBSCOHost. A separate (but identical) search was run on PubMed. The final searches were conducted on 22 January 2024.

#### Search strategy

Table 1 shows the search strategy entered and limiters applied for the searches in both search platforms which is also explained further in the protocol [45].

**Table 1:** Search strategy

Database	Search string	Limiters/expanders
PubMed	(((“Routine data“[Title] OR “routine clinical data“[Title] OR “routinely collected“[Title] OR “routine clinical data“[Title] OR “real world data“[Title] OR “real world evidence“[Title] OR “electronic health“[Title] OR “medical records“[Title] OR “health record“[Title] OR “patient record“[Title] OR “patient data“[Title] OR (“registry“[Title] NOT “trials registry“[Title]) OR “service data“[Title] OR “service evaluation“[Title] OR “audit“[Title] OR “case note“[Title] OR “case notes“[Title]) AND (((“speech“[Title/Abstract] OR “language“[Title/Abstract] OR “occupational“[Title/Abstract] OR “physical“[Title/Abstract] OR “physio“[Title/Abstract]) AND (“therap*“[Title/Abstract] OR “patholog*“[Title/Abstract])) OR “physiotherap*“[Title/Abstract] OR “psycholog*“[Title/Abstract] OR “radiography“[Title/Abstract] OR “radiographer*“[Title/Abstract] OR “paramedic*“[Title/Abstract] OR “biomedical scientist“[Title/Abstract])) NOT (“systematic review“[Title] OR “meta*“[Title] OR “trial“[Title] OR “survey“[Title])) NOT (Interview*[Title/Abstract] OR “focus group“[Title/Abstract] OR “focus groups“[Title/Abstract])	Limiters: English language
EBSCOHost (for CINAHL and MEDLINE)	TI ( “Routine data” OR “routine clinical data” OR “routinely collected” OR “routine clinical data” OR “real world data” OR “real world evidence” OR “electronic health” OR “medical records” OR “health record” OR “patient record” OR “patient data” OR (“registry” NOT “trials registry”) OR “service data” OR “service evaluation” OR “audit” OR “case note” OR “case notes” ) AND AB ( ((speech OR language OR occupational OR physical OR physio) AND (therap* OR patholog*)) OR physiotherap* OR psycholog* OR radiography OR Radiographer* OR paramedic* OR “biomedical scientist” ) NOT TI ( “systematic review” OR meta* OR trial OR survey ) NOT AB ( Interview* OR “focus group” OR “focus groups” )	**Limiters** - Peer Reviewed; Research Article; Publication Type: Academic Journal **Expanders** - Apply equivalent subjects **Search modes** - Boolean/Phrase

*Table 1 Search strategy applied to search CINAHL, MEDLINE Ultimate and PubMed databases*

### Stage 3) Select studies

#### Selection of sources of evidence

Inclusion and exclusion criteria were developed based on the research question and search strategy, to guide the screening process. The criteria were first piloted by three researchers (KC, AP and AC) collectively, on the same 5% of articles to assess reliability, and reach consensus on where additions or changes to the inclusion/exclusion criteria were required to facilitate the screening process and bolster reliability. Following this, titles and abstracts were screened by 2 screeners independently (KC screened 100% and AP and AC screened 50% each). Again, the inclusion and exclusion criteria were discussed at interim points where clarifications were needed, and minor modifications were made as necessary. The final version of the inclusion and exclusion criteria are provided in Table 2. Full-text reviews for eligibility were conducted by two researchers (KC screened 100% and AP and AC screened 50% each which, where possible, were allocated based on expertise).

**Table 2:** Inclusion and exclusion criteria

Inclusion criteria	Exclusion criteria
Study designs which clearly indicate use of routine **clinical (patient)** data, **either prospectively or retrospectively**,** s**o long as the studies are exploring clinical or service-related questions (i.e. data collected in the everyday running of services)	Studies using non-routine data or are very unclear about the data obtained, which encompasses: • Studies using specialised apps or tracking software to collate data that would not otherwise typically be collected in clinical services • Studies using surveys or interviews • Observational studies using other forms of data • Studies using data collected through clinical trials (including in nested forms).
	Studies which use or explore routine data but not for the direct purposes of generating new information relevant to practice, which encompasses: • Studies exploring methodological approaches to analysing routinely collected data (e.g. utilising AI or machine learning) UNLESS a clinical question was also addressed which used the data that was collected/analysed • Studies exploring new approach to collecting routine data (e.g. new databases or data collection tools UNLESS a clinical question was also addressed which used the data that was collected. • Studies exploring perspectives on collecting patient data, using routine-data for research or similar
Studies that **primarily concern** the selected professions (physiotherapists, occupational therapists, radiographers, paramedics, practitioner psychologists, biomedical scientists and speech and language therapists), which also encompasses: • Studies which heavily discuss recommendations for the selected professions, who are stated • Studies which explicitly relate to clinical areas or topics where the involvement of the selected professionals’ is *strongly* implied. Examples of the latter include: *rehabilitation*,* falls*,* mental health services / professions / clinicians*	Studies that do not explicitly relate to the professions listed, which also encompasses: • Studies where reference to the included professionals is limited or not a core part of the study e.g. studies that refer to anxiety levels or mental health diagnoses without referring to psychologists, studies that refer to physical activity without referring to physiotherapist, studies that refer to professions briefly as a ‘future recommendation’ or given as a minority mention in the context of a multi-disciplinary team. • Studies which only report on diagnoses or symptoms without referring to any of the listed professionals who make diagnoses or measure symptoms, or the professionals who may treat them • Studies that relate to dental x-rays which are not performed by radiotherapists.
Studies that are empirical research studies	Studies which are: • Study protocols • Literature reviews, including systematic reviews and meta-analyses • Discussion papers • Commentaries • Conference proceedings or poster abstracts
Studies which are peer-reviewed journal articles which are either written in English or translated to English.	Non peer-reviewed articles Written in languages other than English with no translated version available
Studies where full-texts are retrievable	Studies where no full texts can be retrieved by the existing institutional subscriptions

*Table 2 Inclusion and exclusion criteria developed iteratively through screening and implemented in the scoping review*

### Stage 4) Extracting, mapping and charting the data

#### Data charting process

The data charting framework was initially piloted on five papers by three members of the team (AC, AP, KC) providing an initial opportunity for it to be refined if needed. A random 30% sample of the papers were then charted in line with this, by a first researcher (KC) and a second researcher (AP and AC looking at 15% each). This was to ensure a consistent approach was being taken and provide an opportunity for further modifications to the charting framework should they be required. The first researcher (KC) subsequently completed extraction of all the remaining papers.

#### Data items

Some data items were extracted automatically by Rayyan software (i.e. authors, title, year, journal). The data charting framework ensured capture of the remaining items to both describe the nature of the research and address specific elements central to the research questions. To achieve the latter, we sought to be guided by existing frameworks related to RCD and health equity. Variables extracted from ‘The Reporting of studies Conducted using Observational Routinely-collected health Data (RECORD)’ statement [51] and the ‘NHS CORE20PLUS’ approach which describes a range of population groups likely to experience health inequality were used to guide this section of the data framework [52].

Ultimately, this included the following data items: Title of study, authors, journal of publication, year of publication, Country of first author’s institution, first author's institution (clinical, academic or mixed), clinical and academic composition of the research team, health profession or area of focus, number of datasets used, reference to translating evidence to practice, rationale for study, objective, setting, participants, data sources, methodological approach (qualitative, quantitative, mixed). To address social justice aspects, the following were extracted and mapped as ‘central’ (i.e. a study with a stated aim to address an element of social justice), ‘secondary’ (i.e. a study which had a different primary aim or objective, but still explored an element of social justice as a secondary research aim or objective) or ‘mention’ (i.e. a study which did not explicitly intend to address issues of social justice but nonetheless reported on them): resence of aim pertaining to any facet of social justice, presence of explicit aim pertaining to health inequalities or inequities, presence of aim to include underrepresented populations, presence of aim to undertake intersectional analyses.

### Stage 5) Summarise, synthesize and report the results

#### Synthesis of results

Data was compiled in an Excel spreadsheet comprising the charting framework (as columns) and study information (as rows) to produce a summary table. Quantitative (categorical) data was analysed descriptively through producing basic pivot charts and analysing frequencies (for example, how many studies represented each professional area of practice). Appropriate measures of central tendencies were calculated for the number of datasets used. For qualitative aspects (such as describing the rationale, objective or how the studies addressed components of social justice), summary information recorded was imported into NVivo and analysed inductively by assigning codes at the phrase-level, to create a coding framework used to identify themes. An initial draft report of the results was shared via OSF for consultation between 11 October 2024–18 October 2024, prior to the full analysis and write up in line with the protocol, though no comments or feedback were received and thus no changes made in response to the consultation [44, 45].

## Results

### Selection and characteristics of sources of evidence

**Fig. 1:**
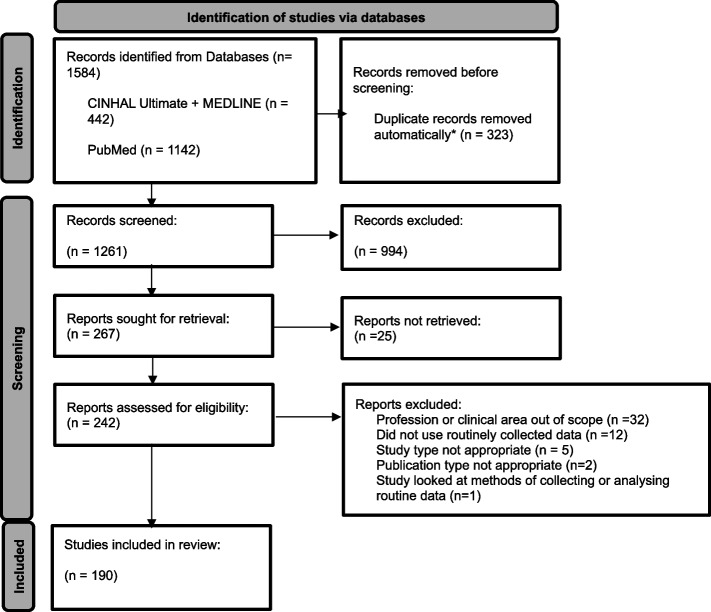
PRIMSA flowchart. Adapted from the PRISMA 2020 flow diagram for new systematic reviews to demonstrate results from database searches and screening procedures

The initial search led to the retrieval of 1584 studies for title/abstract screening against the inclusion and exclusion criteria. The initial agreement rate for title/abstract screening was 86%, leaving 200 conflicts which were resolved by a third screener, resulting in 267 full-text papers being sought for retrieval and assessed for eligibility. Full texts were also screened for eligibility by two screeners, with an initial agreement rate of 82%. After disagreements were resolved, 190 papers were identified as being eligible for inclusion in the review. Papers were excluded for several reasons including not utilising RCD or involving a profession or clinical area that was out of scope. Figure 1 provides the Preferred Reporting Items for Systematic Reviews and Meta-Analyses (PRIMSA) flowchart outlining the search and screening process [53].

### Overview of studies

The data charting framework was considered accurate and comprehensive. Minor adjustments to wording were made through the initial pilot on 5 articles, which was followed by the obtaining of a 100% agreement rate in the extraction of data from a random sample of 55 studies (29%) which were analysed by 2 members of the research team.

The 190 studies included in this analysis was published by authors in institutions across 40 nations, though studies with first authors based in English institutions comprised 42.6% of these (*n* = 81). Studies were associated with a range of allied health fields selected for this review, though over a third of the studies were related to physiotherapy (*n* = 64, 33.7%) and 30 (15.8%) related to psychology / mental health. There were fewer studies on paramedical sciences (*n* = 17, 9.0%) and even fewer associated with speech and language therapy (*n* = 15, 7.9%), occupational therapy (*n* = 15, 7.9%), and radiography /radiotherapy (*n* = 11, 5.8%). Only 1 (0.5%) study was retrieved which related to biomedical sciences. The remainder of the studies (*n* = 37, 19.5%) were relevant to a mix of professions or clinical areas with a range of professions at stake, most noticeably this related to stroke rehabilitation (*n* = 11), accounting for 29.7% of this subgroup. Table 3 summarises this and provides reference to the corresponding studies.

**Table 3:** Summary table of studies and professional or clinical areas

Professional or clinical area	*n*	%	References
Physiotherapy	64	33.7%	[54–117]
Psychology/ mental health	30	15.8%	[118–147]
Paramedical science	17	8.9%	[148–164]
Speech and language therapy	15	7.9%	[165–179]
Occupational therapy	15	7.9%	[180–194]
Radiography/Radiotherapy	11	5.8%	[195–205]
Biomedical sciences	1	0.5%	[206]
Mixed- stroke rehabilitation	11	5.8%	[207–217]
Mixed- other area or combination	26	13.7%	[218–243]
**Grand Total**	**190**	**100.0%**	

*Table 3 Proportion of studies relevant to each profession or clinical area. ‘Mixed- other or combination’ includes studies with combinations of the professions (e.g. speech and language therapy and psychology/mental health) or clinical areas considered to span across professions where they may not be explicitly stated (e.g. pain rehabilitation, falls management, end-of-life care)*

First authors were mostly from clinical institutions (*n* = 84, 44.2%) though there was a substantial proportion of academic first-authors (*n* = 57, 30.0%), and where studies were authored by more than one person, most research teams comprised both (*n* = 115, 60.5%). Although, team compositions varied from purely clinical (*n* = 35, 18.4%), purely academic (*n* = 18, *n* = 9.47%), professional society-led (*n* = 3, 1.6%) or a mixture of academic and professional societies, academic and industry, academic and charitable organisations, or charitable organisations and clinicians (collectively, *n* = 6, 3.2%).

**Fig. 2:**
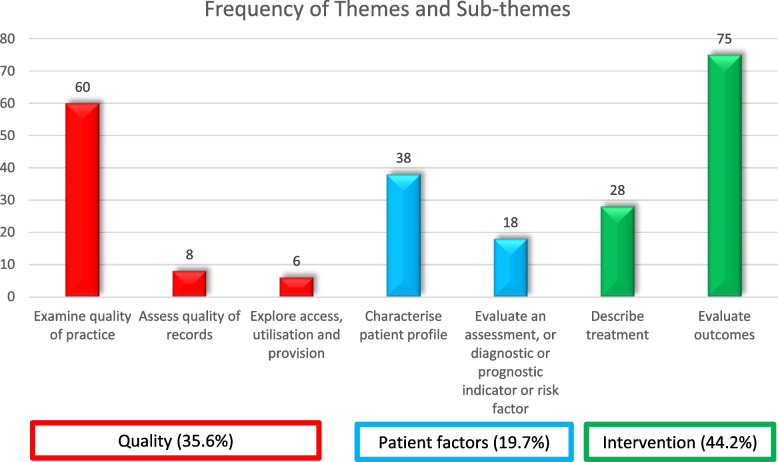
Bar chart illustrating thematic analysis of the aims of the included studies, illustrating the frequency of themes (bottom boxes) and sub-themes (bars)

A third of studies (*n* = 63, 33.2%) explicitly situated their RCD study in the context of a research to practice or research translation gap, however many did not directly do this (*n* = 69, 36.3%) or only partially had this notion (*n* = 57, 30.0%). Three major themes and 8 sub-themes (arising from codes) were identified relating to what the studies aimed to achieve. These were: *quality* *(quality of practice*,* quality of record keeping*,* access to and utilisation of services and quality of provision);* *patient factors* *(profiling a cohort of patients*,* evaluating the utility of a prognostic*,* diagnostic tool*,* assessment or indicator or identifying risk);* and *intervention (describing treatments and evaluating outcomes).* Some studies addressed multiple aims represented across these themes (see Fig. 2).

A full summary chart of the extracted details for the selected studies can be found in the Supplementary Material.

### Datasets and methodologies

Every study utilised patient records in some format via either electronic health systems, registries, logbooks or by linking relevant databases of these kinds (*n* = 190, 100.0%). The size of datasets varied widely though the mean and standard deviation indicated a heavy negative skew (x̄= 16273.80851, SD = 82069.15373). The median therefore as a more appropriate measure was calculated to be 315 (interquartile range = 2098.74). The smallest dataset contained 10 records, whereas the largest was 959,100.

The variables collected and reported differed though some standard fields of patient age and gender emerged. Many studies included forms of outcome measures relevant to the clinical area, however even when clinical areas were the same, there was not always standardisation. For example, ten studies specifically explored back pain [55, 68, 76, 84, 91, 95, 97, 100, 101, 113] and many included a measure of pain severity, however the way this was measured varied study to study. Some made use of visual analogue scales [70], others used formal tools such as the Modified Low Back Pain Disability Questionnaire (MDQ) [55, 101] or less formal approaches such a self-report of pain improvement [84].

Most studies were purely quantitative (*n* = 179, 94.7%) and only one study’s main investigation comprised a qualitative analysis of textual components of medical records (though they did utilise quantitative data to describe the patient cohort) [175]. Several (*n* = 7, 3.2%) adopted a mixed-methods approach [127, 174, 176, 177, 191, 230], with the qualitative components also being analyses of textual components of patient records. A small number of studies were predominantly quantitative but incorporated qualitative elements which were not from RCD, such as questionnaires (*n* = 2, 1.1%).

### Social justice

A total of 27 studies (14.2%) had some degree of association with elements of social justice, and some addressed multiple components of this. Of the 27 studies, only 15 (55.6%) were reported as *centring* social justice in their study. Table 4 provides the distribution of this mapping and corresponding citations.

**Table 4:** Studies exploring elements of social justice

Degree of focus	Social justice		Health inequities		Under-represented groups		Intersectionality	
	**n**	%	**n**	%	**n**	%	**n**	%
Central	14 (83,92,93,118,126–128,130,135,145,161,174,209,219)	7.4%	9 (83,93,118,126–128,130,135,219)	4.7%	8 (83,93,118,126–128,174,221)	4.2%	1 (118)	0.5%
Secondary	5 (84,121,122,182,217)	2.6%	1 (69)	0.5%	3 (130,135,219)	1.6%	0	0.0%
Mention	4 (69,79,134,144)	2.1%	6 (92,121,122,182,209,244)	3.2%	3 (84,169,209)	1.6%	1 (207)	0.5%
Total	23	12.1%	16	8.4%	14	7.4%	2	1.1%

*Table 4 The degree to which studies utilising RCD included in this review addressed elements of social justice*

Of those studies which centred on issues related to social justice, they were largely linked to one of three themes: (1) *equal access to healthcare* (*n* = 7); (2) *marginalised populations or research from post-colonial contexts* (*n* = 4), or (3) *human rights* more broadly (*n* = 3, which included consideration of the rights of people with disabilities and victims of sex trafficking). The studies that explored health inequity mostly looked at a range of social determinants of health and how this was related to health care access, though others were more focused on specific aspects such as equity arising from discrimination due to disability or ethnicity (particularly Aboriginal peoples in Australia). The singular study which centred on intersectionality focused on men who faced multiple levels of disadvantage and their use of mental health services.

It was noted that many of the studies collected data on relevant variables for exploring issues related to social justice including social determinants of health. Socio-economic status, deprivation or educational level was evaluated in 30 studies (15.8%), ethnicity or race was reported on in 25 studies (12.6%), and language spoken in 8 studies (5.7%). However, only very few of these studies operationalised these variables in a way that tackled questions pertinent to social justice. Furthermore, only 6 studies situated themselves as bridging a research-to-practice gap which also focused on an element of social justice [93, 128, 145, 161, 209, 219]. These have been summarised in Table 5 to illustrate the nuances of this particular subset of studies.

**Table 5:** A summary of six studies addressing social justice and research to practice gaps

Reference	Aim of study	Social justice aspect	Research to practice aspect
128	Evaluation of a service for people with a disability and a mental health difficulty	In the service, both the service user and therapist had a disability, mitigating barriers for people with a disability to accessing high quality care.	Disability studies provide a wealth of evidence to support the value of uniquely disability-related shared experiences in therapeutic relationships, but clinical research and practice ignores, and fails to implement this.
161	Evaluation of a model of trauma care provided across regions near the Iran-Iraq border.	Research about what works in trauma care for low- income, war-torn countries prone to complex mass casualties, such as Iraq is essential.	The existing evidence base for trauma care is problematic as there is limited transferability of much paramedical sciences research which is related to models applied in high-income, mostly Western contexts.
219	Exploration of inequalities in access to public neurodevelopmental services.	Analyses on comparisons of service access between families from mainstream and typically marginalised communities (e.g. culturally or linguistically diverse, or socio-economically disadvantaged.	There is an identified challenge of applying best practice guidelines for providing neurodevelopment services in the ‘real world’.
93	Examination of the provision of physiotherapy for children or Australian First Nations heritage with bronchiectasis.	Australian First Nation Children experience the highest rates of bronchiectasis globally, which is associated with a 20-year mortality gap, arising from sub-optimal detection and management.	Best-practice guidance exists, though none of the research focuses on this specific population. The authors highlight that research about *access* to services is lacking.
145	Evaluation of the effectiveness of narrative exposure therapy for female victims of trafficking.	Victims of human trafficking are vulnerable to post-traumatic stress-disorder (PTSD), with specific stressors related to migration status and socio-political contexts, requiring specialist intervention.	Engagements of trafficking victims in trials is challenging and evidence is lacking on how to support this group. Narrative Exposure Therapy (NET) is one intervention developed for use in countries with insecurity and high risk of repeat trauma, but it has not been evidenced in this population yet.
209	Investigation of characteristics influencing the amount of therapy provided in stroke care.	There is substantial variation in the amount of therapy that people receive post-stroke, which may be due to patient factors (such as ethnicity) and organisational factors.	Best practice guidance recommends set amounts of therapy following stroke, but this is rarely implemented, which previous research has highlighted.

Table 5 *A summary of the six identified studies addressing social justice and research to practice gaps, including the study aim and short description of each* *component*

### Narrative summary of results

This analysis has unveiled the extent to which allied health fields are utilising RCD which is substantiated by the volume of studies included in this review (*n* = 190). Whilst there is indicative evidence of the use of these data sources for research across the professions, physiotherapy predominates, and biomedical sciences is significantly underrepresented. Though, it is important this is reflected upon in the context of the relative sizes of the profession (for example, the physiotherapy workforce is substantially larger than the other included professions, at least in the UK [244]) and their fit with more traditional research approaches, where for example, biomedical sciences may be more typically core to pharmaceutical or clinical trials.

A significant finding from this review pertains to the volume of clinician-led research, comprising 44.2% (*n* = 84) of all articles retrieved, including 5.8% (*n* = 11) which were sole-authored. Yet, most clinician-led studies were collaborative and included a team of either fellow clinicians (*n* = 24, 12.6%), academics (*n* = 38, 20%) or a mixed team of clinicians and academics (*n* = 37, 19.5%). Several publications were produced at least in part by authors employed by professional societies (*n* = 9, 4.7%). Only 16 studies (8.4%) were produced by purely academic authors. There were few qualitative studies, though textual elements of patient notes were used for analysis in this way in some articles.

Studies addressing aspects of social justice were limited, and there was little geographic diversity in the publishing institution. Many of the studies come from Central Europe or the Americas (*n* = 141, 74.2%), and these represent 70.4% (*n* = 19) of the articles addressing social justice. There is a slightly higher rate of social-justice focused literature emerging from outside these areas compared to within, which includes Eastern European countries (Turkey), Middle Eastern countries (United Arab Emirates, Israel, Saudi Arabia, Iran, Iraq), Asia more broadly (China, Japan, Pakistan, Thailand), Africa (Nigeria) as well as Pacific Islands (Papua New Guinea) and Australia and New Zealand. According to data extracted in this review, for every 100 papers published in Central Europe or the Americas, 13 address social justice, whereas in areas outside of this, it would be 16 studies. Many more studies had the potential to tackle questions around social justice based on the types of data that they were collecting but did not do so.

## Discussion

This scoping review has revealed the extent to which allied health professions are utilising RCD, which appears to be embraced most in the field of physiotherapy, though is evidently applied by a range of professions. Notably, the studies were very often produced by a diverse team of practitioners in the specific health fields, along with academics. Perhaps reflective of this, some of these studies were explicitly situated in the context of utilisation of RCD to address an identified research-to-practice or implementation gap, though many studies took a narrower focus to address specific local concerns (such as managing waiting lists). Regardless of the context their study was presented in, most related to exploring the quality of services, patient profiles and intervention processes or outcomes. Despite vast volumes of relevant data often being collected, very few studies utilised it to tackle issues pertaining to social justice. However, those that did largely explored factors related to equity of access to health, and health inequalities more broadly. Very rarely was an intersectional lens taken in analysis, which was only identified in one study in this review.

The discussion herein focuses on the application of our findings to produce three recommendations for the consideration of scholarly and clinical communities.

- Recommendation 1: Greater efforts to leverage RWD for research need to be made to unlock new knowledge, which is uniquely placed to complement, challenge, provide context for and - where needed -stand in the absence of traditional clinical research.

Whilst many studies were identified in this review, the volume and scale of RCD in healthcare should be considered to provide greater perspective on the actual extent to which it is leveraged. It is relevant to consider that no date range was applied to our search, thus the 190 studies examined represents the entirety of the scholarly literature retrieved on this topic. Notwithstanding the finding that most of these studies were based in England, there seems a radical underutilisation and/or under-reporting of RCD investigations globally. As such, our scoping review signals multiple avenues in which maximising RCD through research can enrich the evidence base.

Studies in our review often strived to address a research to practice or implementation gap, and a subset of these specifically aimed to describe what ‘routine’ or ‘usual’ care was in their field, by tapping into their patient data. In many allied health fields, ‘real world’ clinical practice is often eclectic and complex with little consensus around what treatments precisely comprise [245, 246]. The value of unpacking this is threefold, since it can be used: (i) by clinicians to understand common approaches to treatment and guide quality of care, (ii) by clinicians and researchers to compare effectiveness of approaches and (iii) by researchers who can grasp better insight into the often-applied ‘catch-all’ of ‘usual care’ in study control arms, and thus improve the rigour of their trials. This role of RCD is also starting to be explored via AI [247].

Furthermore, the focus of studies identified in this review on describing patient profiles similarly spoke to the need to understand ‘real’ populations and the effects of treatments on them. This can similarly serve several purposes for health care providers to better understand who they are (and aren’t) treating, which is particularly valuable for exploring potential health inequalities (though the studies that did this for such a purpose were minimal in this review). Similarly, understanding ‘real world’ clinical populations and trends in these can support research trials in their sampling strategies and participant recruitment, and can be used to challenge the suitability of evidence-based interventions to real world settings and populations. Facilitated understanding of patient cohorts through AI-assisted data mining of RCD is beginning to emerge in the literature [248], indeed some of the studies included in the review were centred on looking at change over time or the impact of a change imposed on services, which could be leveraged in this way.

Similarly, though the number of studies exploring issues related to social justice was small, those that did signalled very valuable findings and recommendations for both practice and research, and thus can serve as exemplars of how RCD can and should be leveraged for these important investigations. An emergent notion from our analysis was the production of RCD studies from lower income countries, where the authors positioned their studies specifically targeting gaps in research arising from an absence or severe lack of research conducted in their context or on their population, critiquing the appropriateness of largely Western-produced knowledge about health and healthcare. This finding especially illustrates the unique value of RWD studies in a global health context, bringing into focus the tacit knowledge of local actors and local realities, contributing to the decolonisation of health [249].

However, whilst leveraging RCD for these purposes clearly has benefits, this review does reveal a need for greater standardisation of the ways in which data is recorded. Standardisation of treatment outcome measures and the ways treatments are described would maximise data linkage opportunities and enhance the added-value RCD offers to research. Data relevance, completeness and quality issues were commonly cited in the papers in this scoping review, and are often discussed as a significant limitation of leveraging RCD in other studies [250, 251]. The potential of RCD studies would be infinitely greater through the development and adoption of minimum data sets (MDSs) which establish data-collection protocols for clinical conditions or areas. Yet, for MDSs to really solve this issue, they need to be feasible for practitioners to adhere to in an every-day, busy clinical setting (for an example, see work by Harvey et al. involving a project to co-produce an MDS for aphasia, with researchers, clinicians and people with aphasia [252]). Doing so would enhance the quality of data and the potential for more meaningful analyses of RCD, which may also be assisted by AI powered analysis of electronic patient notes [253].

- Recommendation 2: Practitioners should be positioned as the experts for asking the questions of RCD, to expose challenges in implementation of traditional evidence, unveil complexities of clinical practice, and create actionable evidence.

As highlighted through this scoping review, RCD investigations can often explicitly address a research translation gap and support evidence-based practice for on-the-ground clinicians. Recognising the unique perspectives of clinicians in driving RCD studies forward is imperative for the full potential of such studies to be met and their value to be recognised. Exemplified through the comparatively high proportion of clinician-led versus purely academically driven research in our review, practitioners are best-placed to identify the limitations and challenges in the existing evidence base or evidence-based guidance for implementation in the real-world and ask pertinent questions to be explored via their patient data. Many of the studies we reviewed utilised RCD to audit and scrutinise clinical practice compared with best-practice standards or guidelines, often revealing struggles with adhering to them. This ‘practice-based evidence’ created through an RCD study provides important context and rationale for the reconsideration of service design on the one hand or inadequacies of guidelines on the other, as well as new avenues for research and ways to tackle health inequities [254]. This notion resonates with that of knowledge mobilisation, where insights from RCD provide reason for “*collective making”* of knowledge by on-the-ground actors [255]. Such knowledge may be more likely to be actioned in practice (compared with otherwise-distanced ‘research’) due to a greater sense of ownership and thus greater potential for implementation [255].

Often, studies included in this review tapped into their patient data to gather snapshots of their clinical services and generate real-time evidence to inform practice when needed and examine the impact of change. For example, several studies included in this review produced insights to examine the impact of COVID-19 on their health care services and patient outcomes, as well as new conditions where evidence is lacking but very much needed, such as Long Covid [167]. These studies illustrate the usefulness of RCD in unprecedented situations, which cannot always ‘wait’ for large scale trials or longitudinal studies to create required evidence. Echoing the points in the previous section, this novel intelligence can also be highly valuable for planning future clinical studies.

Respecting and empowering allied health practitioners to lead research, which can be in collaboration with academic researchers, has emerged as a strategic focus particularly within the UK (for example, in 2022, NHS England published an Allied Health Professions Research and Innovation Strategy [256]), a move which is supported by the findings of our review highlighting the value of clinician-led and prioritised research in closing research to practice gaps via RCD studies. Scholars are beginning to advocate for a shift towards welcoming practitioners into ‘academic spaces’ to specifically address research translation and evidence-based practice, which is also recognised by clinicians themselves [257, 258]. Moreover, there is growing evidence that clinician engagement in research can directly benefit patient outcomes [259]. Recent advances in supporting allied health practitioners to develop the skills and capabilities have been made, in the UK [260], though given the extent of reported challenges and barriers for on-the-ground allied health clinicians to engage in research [261, 262], it is yet to be seen if this concerted effort overcomes them. Our scoping review adds further impetus for the need for specific strategies to embed research capability in clinical roles, showcasing the unique value of practitioner-led research, especially utilising RCD.

- Recommendation 3: Challenge received wisdom, traditional hierarchies of evidence and research paradigms and expand the academic gaze to value RCD studies (of any magnitude), especially for their role in exploring research applicability, translation and exposing issues related to social justice.

One consideration that may partly explain the discrepancy between the extent to which RCD research has been published – as identified in this review - and it’s potential to contribute to the evidence base and tackle issues pertaining to social justice is the often-narrow perception of what constitutes ‘high quality research’. Whilst it has been remodelled, redeveloped and reclassified overtime, the classic hierarchy of evidence – its original conceptualisation of which is often attributed to pioneer of ‘evidence-based medicine’, David Sackett [263] – places non-controlled and observational studies, evidence generated by single-sites, single-cases and practice-based evidence including studies utilising RCD such as audits, or service evaluations, at the bottom of the hierarchy, signalling the limitations of their rigour, and consequently indicate caution about their appropriateness to inform practice [263]. Supportive of this traditional paradigm, significant warnings and many valid concerns have been voiced about Big Data health research (typically making use of RCD) due to the inherent biases arising at the level of data collection, including those that arise from practitioners’ and providers’ own biases as well as challenges in controlling variables and eliminating ‘noise’ [6, 167, 250, 251]. Despite this cautionary approach, there have been substantial efforts by major health care stakeholders to promote large scale routine data collection for the production of real-world evidence [3–5], investments by AI companies to exploit it, and medical studies to operationalise it. As such, there remains a mismatch in what forms of research have been traditionally perceived of as high quality and value (and those which are not), and what the contemporary priorities for health care are. RCD studies are thus situated amidst this contention, indeed where real-world evidence is posited as a “disruptive force” [264].

Furthermore, examining the classical hierarchy of evidence and research paradigms through the lens of social justice unveils the likelihood that traditional research produces bodies of evidence which are significantly biased (towards certain populations due to underrepresentation of marginalised groups), inappropriate (by centring Western models of medicine and perpetuating barriers to healthcare) and which simply fail to be useful for the global majority (by largely evaluating service delivery and provision that is unobtainable and unsuitable in middle and low income countries). Judgement on research *quality*, as considered through classic paradigms, is thereby challenged. Yet, even RCD studies are not without risk of creating evidence that could reproduce structural and societal injustices – which may be perpetuated by AI especially [265–267] - and it is imperative to adopt a critical lens when reporting findings from such studies. The ‘*QuantCrit’* framework carefully sets out recommendations for how quantitative data, such as Big Data in health, can be operationalised for social justice; an example being the imperative of when examining the variable of ‘race’ to “*read ‘racism’*”, thereby highlighting an “*operation of racism*” rather than ‘race’ being “*a cause in its own right*” [268]. There is a need therefore to carefully examine *how* data is utilised in this way (whether by AI or else) and scrutinise the forces at play which may skew findings at all levels of data collection, analysis and importantly, interpretation. Whilst clearly not the sole answer to tackling these issues and avoiding “*data chauvinism*” [25], we argue that RCD studies have the potential to re-orient and re-balance the evidence base in certain ways to mitigate the perpetuation of social injustices regarding health and health care.

The conflicting schools of thought regarding utilisation of RCD need to be remedied through a reconceptualization of the ‘hierarchy’ of evidence [269]. Resonating with recent *Nature* ponderings [57], emerging calls to decolonise health research [249, 270] and long-standing recommendations from the WHO commission [271], our scoping review supports the need for a departure from the orthodox (and colonial) conceptions of research *quality* and *value* and argue for a shift towards accepting a more flexible and dynamic relationship between and across diverse research approaches, which acknowledges the unique and powerful place that RCD studies and practice-based research (of any magnitude) can occupy in: (a) the production and translation of research into practice (b) improvements to clinical trials and (c) tackling social justice. Widening our definition of research *quality* and understanding of research *value*, and positioning RCD within this, can ultimately develop and enhance the evidence-base and optimise care for *all *patients.

### Limitations

Whilst this scoping review has been expansive, it is nonetheless limited by its conservative search strategy which was largely developed pragmatically to ensure successful and timely completion of the study. However, it is also possible that studies were missed given our selective database choices and the skew in expertise in the team despite attempts to bring in targeted collaborators. During the screening process of the articles that were retrieved, it is possible that the researchers’ biases and naivete of certain clinical topics or professionals’ scope of practice meant that papers were incorrectly excluded. For example, the identification of only one study in the biomedical sciences profession could reflect that this profession is perhaps more removed from the clinical expertise within the team and other studies relevant to this field may have been excluded.

Additionally, our data extraction framework is somewhat reductionist in the way in which it aims to capture complex topics such as social justice, which is subsequently reflected in our synthesis. However, by identifying and mapping the studies in this way, we hope it signals potential future avenues for further exploration of the work done in these areas. For example, it is likely that a narrative literature review may be well suited to exploring the studies that aimed to address issues of social justice in more detail. It is also important to note and reflect on the positionality and location of the research team undertaking this work (all are White women employed by an academic institution in England), from a greatly privileged perspective. Our discourse on social justice, and approach to this research, is thereby highly likely to be influenced by this and we are aware there is an absence of voice and perspective from people who have experienced greater social injustices who may bring a more critical and meaningful lens to the review.

## Conclusions

This scoping review has underscored that there is much potential in leveraging RCD to bridge challenges in evidence-based practice, as well as adding context to traditional research methodologies, and can address pertinent social issues such as health inequities, providing information on client groups with complex, rare conditions who are frequently excluded from such studies, and interrogating injustices in allied health fields. However, to date, there is a relatively low representation of these studies in the literature. Our synthesis underpins three recommendations for consideration by academics, practitioners, and the applied health scholarly community more broadly, which advocate for greater: recognition and use of health data, enablement of practitioner-led research and consideration of diverse research forms and their dynamic interplay. We urge adoption of a ‘*QuantCrit’* approach [268] to doing so, where subsequently the evidence-base underpinning clinical practice can be expanded, enriched, and fit for purpose to enable equitable living and health for all. Future research exploring standardisation of data collection across providers and patients and on effective strategies to enhance clinician-researcher capability will be useful to drive this agenda forward. Furthermore, an examination of approaches to data collection and analysis which minimise the risk of reproducing social injustices – with and without AI- and greater consideration of long-standing research paradigms would be central to advancing our understanding of how RCD can be operationalised effectively to further the pursuit of social justice.

## Supplementary Information

Supplementary Material 1.

## Data Availability

No datasets were generated or analysed during the current study.

## Abbreviations

RCD: Routinely collected data
RWD: Real world data
OSF: Open Science Framework
AI: Artificial Intelligence
PRIMSA: Preferred Reporting Items for Systematic Reviews and Meta-Analyses

## References

[1] Chodankar D. Introduction to real-world evidence studies. Perspect Clin Res. 2021;12(3):171–4. 34386383 10.4103/picr.picr_62_21 PMC8323556

[2] Sherman RE, Anderson SA, Dal Pan GJ, Gray GW, Gross T, Hunter NL, et al. Real-world evidence — what is it and what can it tell us? N Engl J Med. 2016;375(23):2293–7. 27959688 10.1056/NEJMsb1609216

[3] World Health Organization. Data collection tools - WHO. Available from: https://www.who.int/data/data-collection-tools. Cited 2024 Jun 27.

[4] Commissioner O of the. FDA. FDA. 2024. Real-World Evidence. Available from: https://www.fda.gov/science-research/science-and-research-special-topics/real-world-evidence. Cited 2024 Oct 17.

[5] National Institute for Health and Care Excellence. NICE real-world evidence framework. 2022. Available from: https://www.nice.org.uk/corporate/ecd9/chapter/overview. Cited 2022 Oct 26.

[6] Grimberg F, Asprion PM, Schneider B, Miho E, Babrak L, Habbabeh A. The real-World Data challenges Radar: a review on the challenges and risks regarding the Use of Real-World Data. Digit Biomark. 2021;5(2):148–57. 34414352 10.1159/000516178 PMC8339486

[7] McDonald L, Lambrelli D, Wasiak R, Ramagopalan SV. Real-world data in the United Kingdom: opportunities and challenges. BMC Med. 2016;14(1):97. 27342341 10.1186/s12916-016-0647-x PMC4921013

[8] Motheral BR, Fairman KA. The use of claims databases for outcomes research: rationale, challenges, and strategies. Clin Ther. 1997;19(2):346–66. 9152572 10.1016/s0149-2918(97)80122-1

[9] Stey AM, Russell MM, Ko CY, Sacks GD, Dawes AJ, Gibbons MM. Clinical registries and quality measurement in surgery: a systematic review. Surgery. 2015;157(2):381–95. 25616951 10.1016/j.surg.2014.08.097

[10] Cooner F, Liao R, Lin J, Barthel S, Seifu Y, Ruan S. Leveraging real-World Data in COVID-19 response. Stat Biopharm Res. 2022;0(0):1–14.

[11] Franklin JM, Schneeweiss S. When and how can real world data analyses substitute for randomized controlled trials?. Clin Pharmacol Ther. 2017;102(6):924–33. 28836267 10.1002/cpt.857

[12] Mc Cord KA, Al-Shahi Salman R, Treweek S, Gardner H, Strech D, Whiteley W, et al. Routinely collected data for randomized trials: promises, barriers, and implications. Trials. 2018;19(1):29. 29325575 10.1186/s13063-017-2394-5 PMC5765645

[13] Monti S, Grosso V, Todoerti M, Caporali R. Randomized controlled trials and real-world data: differences and similarities to untangle literature data. Rheumatology. 2018;57(Supplement7):vii54–8. 30289534 10.1093/rheumatology/key109

[14] Partridge N, Scadding J. The James Lind Alliance: patients and clinicians should jointly identify their priorities for clinical trials. Lancet. 2004;364(9449):1923–4. 15566996 10.1016/S0140-6736(04)17494-1

[15] Avery M, Westwood G, Richardson A. Enablers and barriers to progressing a clinical academic career in nursing, midwifery and allied health professions: a cross-sectional survey. J Clin Nurs. 2022;31(3–4):406–16. 33507578 10.1111/jocn.15673

[16] Trusson D, Rowley E, Bramley L. A mixed-methods study of challenges and benefits of clinical academic careers for nurses, midwives and allied health professionals. BMJ Open. 2019;9(10):e030595. 31594886 10.1136/bmjopen-2019-030595 PMC6797317

[17] Pager S, Holden L, Golenko X. Motivators, enablers, and barriers to building allied health research capacity. J Multidisciplinary Healthc. 2012;5:53–9. 10.2147/JMDH.S27638 PMC3292402 22396626

[18] Palmer S, Coad J, Gamble J, Jones C, Lees-Deutsch L, McWilliams D, et al. Nursing, midwifery, and allied health professions research capacities and cultures: a survey of staff within a university and acute healthcare organisation. BMC Health Serv Res. 2023;23(1):647. 37328877 10.1186/s12913-023-09612-3 PMC10276387

[19] Johnson CE, Whiteside YO. Real-world evidence for Equality. Health Equity. 2021;5(1):724–6. 34909542 10.1089/heq.2020.0136 PMC8665792

[20] Buettner-Schmidt K, Lobo ML. Social Justice: a concept analysis. J Adv Nurs. 2012;68(4):948–58. 22032609 10.1111/j.1365-2648.2011.05856.x

[21] Moorthie S, Peacey V, Evans S, Phillips V, Roman-Urrestarazu A, Brayne C, et al. A scoping review of approaches to improving quality of data relating to Health inequalities. Int J Environ Res Public Health. 2022;19(23):15874. 36497947 10.3390/ijerph192315874 PMC9740714

[22] He Z, Pfaff E, Guo SJ, Guo Y, Wu Y, Tao C, et al. Enriching real-world data with Social Determinants of Health for Health Outcomes and Health Equity: successes, challenges, and opportunities. Yearb Med Inform. 2023;32(1):253. 38147867 10.1055/s-0043-1768732 PMC10751148

[23] Shavers-Hornaday VL, Lynch CF, Burmeister LF, Torner JC. Why are African americans under‐represented in medical research studies? Impediments to participation. Ethn Health. 1997;2(1–2):31–45. 9395587 10.1080/13557858.1997.9961813

[24] Seto B. History of Medical Ethics and perspectives on disparities in Minority Recruitment and Involvement in Health Research. Am J Med Sci. 2001;322(5):246–50. 11876183

[25] Lee EWJ, Viswanath K. Big Data in Context: addressing the Twin perils of Data Absenteeism and Chauvinism in the Context of Health Disparities Research. J Med Internet Res. 2020;22(1):e16377. 31909724 10.2196/16377 PMC6996749

[26] Liaw ST, Taggart J, Dennis S, Yeo A. Data quality and fitness for purpose of routinely collected data – a general practice case study from an electronic Practice-Based Research Network (ePBRN). AMIA Annu Symp Proc. 2011;2011:785. 22195136 PMC3243124

[27] George S, Duran N, Norris K. A systematic review of barriers and facilitators to Minority Research Participation among African americans, latinos, Asian americans, and Pacific islanders. Am J Public Health. 2014;104(2):e16–31. 24328648 10.2105/AJPH.2013.301706 PMC3935672

[28] Steinbrenner JR, McIntyre N, Rentschler LF, Pearson JN, Luelmo P, Jaramillo ME, et al. Patterns in reporting and participant inclusion related to race and ethnicity in autism intervention literature: data from a large-scale systematic review of evidence-based practices. Autism. 2022;26(8):2026–40. 35068190 10.1177/13623613211072593 PMC9596958

[29] De Las Nueces D, Hacker K, DiGirolamo A, Hicks LS. A systematic review of community-based Participatory Research to enhance clinical trials in racial and ethnic minority groups. Health Serv Res. 2012;47(3pt2):1363–86. 22353031 10.1111/j.1475-6773.2012.01386.x PMC3418827

[30] Shaw AR, Perales-Puchalt J, Johnson E, Espinoza-Kissell P, Acosta-Rullan M, Frederick S, et al. Representation of racial and ethnic minority populations in Dementia Prevention trials: a systematic review. J Prev Alzheimers Dis. 2022;9(1):113–8. 35098981 10.14283/jpad.2021.49 PMC8804327

[31] Powell RA, Njoku C, Elangovan R, Sathyamoorthy G, Ocloo J, Thayil S, et al. Tackling racism in UK health research. BMJ. 2022;376:e065574. 35042720 10.1136/bmj-2021-065574 PMC8764577

[32] Wemrell M, Karlsson N, Perez Vicente R, Merlo J. An intersectional analysis providing more precise information on inequities in self-rated health. Int J Equity Health. 2021;20(1):54. 33536038 10.1186/s12939-020-01368-0 PMC7856780

[33] Mahendran M, Lizotte D, Bauer GR. Describing Intersectional Health outcomes: an evaluation of Data Analysis methods. Epidemiology. 2022;33(3):395. 35213512 10.1097/EDE.0000000000001466 PMC8983950

[34] Holman D, Salway S, Bell A, Beach B, Adebajo A, Ali N, et al. Can intersectionality help with understanding and tackling health inequalities? Perspectives of professional stakeholders. Health Res Policy Syst. 2021;19(1):97. 34172066 10.1186/s12961-021-00742-w PMC8227357

[35] Canfell OJ, Davidson K, Sullivan C, Eakin E, Burton-Jones A. Data sources for precision public health of obesity: a scoping review, evidence map and use case in Queensland, Australia. BMC Public Health. 2022;22(1):584. 35331189 10.1186/s12889-022-12939-x PMC8953390

[36] Stewart J, Kee F, Hart N. Using routinely collected primary care records to identify and investigate severe asthma: a scoping review. npj Prim Care Respir Med. 2021;31(1):1–11. 33500422 10.1038/s41533-020-00213-9 PMC7838272

[37] Kent L, McGirr M, Eastwood KA. Global trends in prevalence of maternal overweight and obesity: a systematic review and meta-analysis of routinely collected data retrospective cohorts. Int J Popul Data Sci. 2024;9(2). 10.23889/ijpds.v9i2.2401 PMC12045326 40313349

[38] Millar C, Carey LB, Hill AE, Attrill S, Avgoulas MI, Drakopoulos E, et al. Global citizenship and social justice among speech-language pathologists: a scoping review. J Commun Disord. 2023;103:106317. 36893492 10.1016/j.jcomdis.2023.106317

[39] Synovec CE, Aceituno L. Social Justice considerations for occupational therapy: the role of addressing social determinants of health in unstably housed populations. Work. 2020;65(2):235–46. 32007967 10.3233/WOR-203074

[40] Morisod K, Luta X, Marti J, Spycher J, Malebranche M, Bodenmann P. Measuring Health Equity in Emergency Care using routinely Collected Data: a systematic review. Health Equity. 2021;5(1):801–17. 35018313 10.1089/heq.2021.0035 PMC8742300

[41] Barker LC, Hussain-Shamsy N, Rajendra KL, Bronskill SE, Brown HK, Kurdyak P, et al. The use of key social determinants of health variables in psychiatric research using routinely collected health data: a systematic analysis. Soc Psychiatry Psychiatr Epidemiol. 2023;58(2):183–91. 36149450 10.1007/s00127-022-02368-x

[42] Grant MJ, Booth A. A typology of reviews: an analysis of 14 review types and associated methodologies. Health Inform Libr J. 2009;26(2):91–108. 10.1111/j.1471-1842.2009.00848.x 19490148

[43] Sweetman D, Badiee M, Creswell JW. Use of the transformative Framework in mixed methods studies. Qualitative Inq. 2010;16(6):441–54.

[44] Chadd K, Caute A, Pettican A, Enderby P. Open Science Framework - How is routinely collected data being used in allied health research? A scoping review. 2023 [cited 2024 Oct 18]. How is routinely collected data being used in allied health research? A scoping review. Available from: https://osf.io/re4ym/.

[45] Chadd K, Caute A, Pettican A, Enderby P. Methods to advance health equity and social justice in healthcare: protocol for a scoping review on the utilisation of routinely collected data. PLoS ONE. 2024;19(7):e0306786. 38985705 10.1371/journal.pone.0306786 PMC11236175

[46] Arksey H, O’Malley L. Scoping studies: towards a methodological framework. Int J Soc Res Methodol. 2005;8(1):19–32.

[47] Westphaln KK, Regoeczi W, Masotya M, Vazquez-Westphaln B, Lounsbury K, McDavid L, et al. From Arksey and O’Malley and Beyond: Customizations to enhance a team-based, mixed approach to scoping review methodology. MethodsX. 2021;8:101375. 34430271 10.1016/j.mex.2021.101375 PMC8374523

[48] Tricco AC, Lillie E, Zarin W, O’Brien KK, Colquhoun H, Levac D, et al. PRISMA Extension for scoping reviews (PRISMA-ScR): Checklist and Explanation. Ann Intern Med. 2018;169(7):467–73. 30178033 10.7326/M18-0850

[49] Cooke A, Smith D, Booth A, Beyond PICO. The SPIDER Tool for qualitative evidence synthesis. Qual Health Res. 2012;22(10):1435–43. 22829486 10.1177/1049732312452938

[50] Methley AM, Campbell S, Chew-Graham C, McNally R, Cheraghi-Sohi S. PICO, PICOS and SPIDER: a comparison study of specificity and sensitivity in three search tools for qualitative systematic reviews. BMC Health Serv Res. 2014;14(1):579. 25413154 10.1186/s12913-014-0579-0 PMC4310146

[51] Benchimol EI, Smeeth L, Guttmann A, Harron K, Moher D, Petersen I, et al. The REporting of studies conducted using Observational routinely-collected health data (RECORD) Statement. PLoS Med. 2015;12(10):e1001885. 26440803 10.1371/journal.pmed.1001885 PMC4595218

[52] England NHS. Core20PLUS5 – An approach to reducing health inequalities for children and young people. Available from: https://www.england.nhs.uk/about/equality/equality-hub/national-healthcare-inequalities-improvement-programme/core20plus5/core20plus5-cyp/. Cited 2023 Sep 25.

[53] Page MJ, McKenzie JE, Bossuyt PM, Boutron I, Hoffmann TC, Mulrow CD, et al. The PRISMA 2020 statement: an updated guideline for reporting systematic reviews. BMJ. 2021;372:n71. 33782057 10.1136/bmj.n71 PMC8005924

[54] Broman D, Piussi R, Thomeé R, Hamrin Senorski E. A clinician-friendly test battery with a passing rate similar to a ‘gold standard’ return-to-sport test battery 1 year after ACL reconstruction: results from a rehabilitation outcome registry. Phys Ther Sport. 2023;59:144–50. 36566585 10.1016/j.ptsp.2022.12.009

[55] Lutz AD, Windsor BA, Shanley E, Denninger TR, Harrington SE, Thigpen CA. A comparison of treatment signatures of high and low performing physical therapists for patients with lower back pain: analysis of spine care from a physical therapy outcomes registry. Spine J. 2022;22(5):847–56. 34813956 10.1016/j.spinee.2021.11.008

[56] Smith MF, Hillman R. A retrospective service audit of a mobile physiotherapy unit on the PGA European golf tour. Phys Ther Sport. 2012;13(1):41–4. 22261430 10.1016/j.ptsp.2010.09.001

[57] Barratt PA, Selfe J. A service evaluation and improvement project: a three year systematic audit cycle of the physiotherapy treatment for lateral epicondylalgia. Physiotherapy. 2018;104(2):209–16. 29366541 10.1016/j.physio.2017.09.001

[58] Paling C, Hutting N, Devoto K, Galdeano J, Josling K, Goodway L. A service evaluation of the management of patients with suspected cauda equina syndrome from an outpatient physiotherapy service in the United Kingdom. Musculoskelet Sci Pract. 2022;62:102673. 36335852 10.1016/j.msksp.2022.102673

[59] Gardiner J, Turner P. Accuracy of clinical diagnosis of Internal Derangement of the knee by extended scope physiotherapists and orthopaedic doctors: retrospective audit. Physiotherapy. 2002;88(3):153–7.

[60] Liffen N. Achilles tendon diagnostic ultrasound examination: a locally designed protocol and audit. Int Musculoskelet Med. 2014;36(1):1–12.

[61] Gomes YE, Chau M, Banwell HA, Davies J, Causby RS. Adequacy of clinical information in X-ray referrals for traumatic ankle injury with reference to the Ottawa Ankle Rules—a retrospective clinical audit. PeerJ. 2020;8:e10152. 33083152 10.7717/peerj.10152 PMC7548068

[62] Fennelly O, Blake C, FitzGerald O, Breen R, Ashton J, Brennan A, et al. Advanced practice physiotherapy-led triage in Irish orthopaedic and rheumatology services: national data audit. BMC Musculoskelet Disord. 2018;19:181. 29859072 10.1186/s12891-018-2106-7 PMC5984783

[63] Lubrano E, Butterworth M, Hesselden A, Wells S, Helliwell P. An audit of anthropometric measurements by medical and physiotherapy staff in patients with ankylosing spondylitis. Clin Rehabil. 1998;12(3):216–20. 9688037 10.1191/026921598675367725

[64] Al-Mandeel MM, Watson T. An audit of patient records into the nature of pulsed shortwave therapy use… including commentary by Dziedzic K, and Callaghan MJ. Int J Therapy Rehabilitation. 2006;13(9):414–20.

[65] Paim T, Low-Choy N, Dorsch S, Kuys S. An audit of physiotherapists’ documentation on physical activity assessment, promotion and prescription to older adults attending out-patient rehabilitation. Disabil Rehabilitation. 2022;44(8):1537–43. 10.1080/09638288.2020.1805644 32809850

[66] Sumner M, Mead J, ten Hove R. Audit and re-audit of the CSP *Core standards of Physiotherapy Practice*. Physiotherapy. 2000;86(10):512–6.

[67] Hempling M, Adhikari A. Audit of fractured neck of femur integrated care pathway. J Integr Care Pathways. 2005;9(3):106–8.

[68] Wertli MM, Held U, Lis A, Campello M, Weiser S. Both positive and negative beliefs are important in patients with spine pain: findings from the Occupational and Industrial Orthopaedic Center registry. Spine J. 2018;18(8):1463–74. 28756302 10.1016/j.spinee.2017.07.166

[69] Thrush AH. Cardiac Rehabilitation in Abu Dhabi: a Retrospective Investigation of Program Delivery, participants, and Factors Associated with Program Completion utilizing a Hospital Registry. J Saudi Heart Assoc 35(3):235–43. 10.37616/2212-5043.1349 PMC10597596 37881595

[70] Baumbach L, Grønne DT, Møller NC, Skou ST, Roos EM. Changes in physical activity and the association between pain and physical activity – a longitudinal analysis of 17,454 patients with knee or hip osteoarthritis from the GLA:D^®^ registry. Osteoarthr Cartil. 2023;31(2):258–66. 10.1016/j.joca.2022.09.012 36272673

[71] Cottrell M, Judd P, Comans T, Easton P, Chang AT. Comparing fly-in fly-out and telehealth models for delivering advanced-practice physiotherapy services in regional Queensland: an audit of outcomes and costs. J Telemedicine Telecare. 2021;27(1):32–8. 10.1177/1357633X19858036 31280639

[72] Truscott B, Worsam B. Criteria audit as a means of assessing the physiotherapy component of stroke rehabilitation. Aust J Physiother. 1982;28(5):3–9. 25026109 10.1016/S0004-9514(14)60775-3

[73] Bateman M, Whitby E, Kacha S, Salt E. Current physiotherapy practice in the management of tennis elbow: a service evaluation. Musculoskelet Care. 2018;16(2):322–6. 10.1002/msc.1236 29469176

[74] Barlow T, Rhodes-Jones T, Ballinger S, Metcalfe A, Wright D, Thompson P. Decreasing the number of arthroscopies in knee osteoarthritis – a service evaluation of a de-implementation strategy. BMC Musculoskelet Disord. 2020;21(1):140. 32126992 10.1186/s12891-020-3125-8 PMC7055049

[75] Chou A, Johnson JK, Jones DB, Euloth T, Matcho BA, Bilderback A, et al. Effects of an electronic health record-based mobility assessment and automated referral for inpatient physical therapy on patient outcomes: a quasi-experimental study. Health Serv Res. 2023;58(S1):51–62. 36271503 10.1111/1475-6773.14087 PMC9843085

[76] Wingood M, Vincenzo J, Gell N. Electronic health record data extraction: physical therapists’ documentation of physical activity assessments and prescriptions for patients with chronic low back pain. Physiother Theory Pract 0(0):1–10. 10.1080/09593985.2023.2274385 PMC11058108 37902255

[77] Corone S, Iliou MC, Pierre B, Feige JM, Odjinkem D, Farrokhi T, et al. French registry of cases of type I acute aortic dissection admitted to a cardiac rehabilitation center after surgery. Eur J Cardiovasc Prev Rehabilitation. 2009;16(1):91–5. 10.1097/HJR.0b013e32831fd6c8 19237998

[78] White PD, Naish VAB. Graded exercise therapy for chronic fatigue syndrome: an audit. Physiotherapy. 2001;87(6):285–8.

[79] Stephens G, O’Neill S, Clifford C, Cuff A, Forte F, Hawthorn C, et al. Greater trochanteric pain syndrome in the UK National Health Service: a multicentre service evaluation. Musculoskelet Care. 2019;17(4):390–8. 10.1002/msc.1419 31469233

[80] Oostendorp RA, Elvers H, van Trijffel E, Rutten GM, Scholten-Peeters GG, Heijmans M, et al. Has the quality of physiotherapy care in patients with whiplash-associated disorders (WAD) improved over time? A retrospective study using routinely collected data and quality indicators. Patient Prefer Adherence. 2018;12:2291–308. 30519001 10.2147/PPA.S179808 PMC6233472

[81] McDonell I, Barr C, van den Berg M. Implementing circuit class training can increase therapy time and functional independence in people with stroke receiving inpatient rehabilitation: findings from a retrospective observational clinical audit. Physiother Theory Pract. 2024;40(7):1383–9. 36724415 10.1080/09593985.2023.2172634

[82] Oostendorp RAB, Elvers H, van Trijffel E, Rutten GM, Scholten-Peeters GGM, De Kooning M et al. Improved quality of physiotherapy care in patients with Whiplash-Associated Disorders: Results based on 16 years of routinely collected data. Frontiers in Pain Research. 2022;3. Available from: https://www.frontiersin.org/articles/. Cited 2023 Sep 8. 10.3389/fpain.2022.929385 PMC9468444 36110289

[83] Degerstedt F, Enberg B, Keisu B, Björklund M, Keisu BI. Inequity in physiotherapeutic interventions for children with cerebral palsy in Sweden-A national registry study. Acta Paediatr. 2020;109(4):774–82. 31435959 10.1111/apa.14980

[84] Al-Eisa E. Indicators of adherence to physiotherapy attendance among Saudi female patients with mechanical low back pain: a clinical audit. BMC Musculoskelet Disord. 2010;11:124. 20565719 10.1186/1471-2474-11-124 PMC2903506

[85] Wheatley-Smith L, McGuinness S, Wilson FC, Scott G, McCann J, Caldwell S. Intensive physiotherapy for vegetative and minimally conscious state patients: a retrospective audit and analysis of therapy intervention. Disabil Rehabilitation. 2013;35(12):1006–14. 10.3109/09638288.2012.720355 23009212

[86] Wertli MM, Held U, Campello M, Schecter Weiner S. Obesity is associated with more disability at presentation and after treatment in low back pain but not in neck pain: findings from the OIOC registry. BMC Musculoskelet Disord. 2016;17:1–14. 27036857 10.1186/s12891-016-0992-0 PMC4815184

[87] O’Farrell S, Smart KM, Caffrey A, Daly O, Doody C. Orthopaedic triage at a physiotherapist-led ‘Musculoskeletal Assessment Clinic’: a seven-month service evaluation of outcomes. Ir J Med Sci. 2014;183(4):565–71. 24337981 10.1007/s11845-013-1052-5

[88] Monaghan C, Haywood A. Pelvic girdle pain - part 1: quantitative results from a mixed-methods service evaluation introducing a manual therapy treatment approach to usual care. J Pelvic Obstetric Gynaecol Physiotherapy. 2016;119:47–55.

[89] Downie F, McRitchie C, Monteith W, Turner H. Physiotherapist as an alternative to a GP for musculoskeletal conditions: a 2-year service evaluation of UK primary care data. Br J Gen Pract. 2019;69(682):e314–20. 30962224 10.3399/bjgp19X702245 PMC6478452

[90] Caffrey A, Smart KM, FitzGerald O. Physiotherapist-led triage at a Rheumatology‐Based Musculoskeletal Assessment Clinic: an 18‐Month Service evaluation of activity and outcomes. ACR Open Rheumatol. 2019;1(4):213–8. 31777797 10.1002/acr2.1022 PMC6858023

[91] Davies F, Pace J, Angus M, Chan-Braddock S, Jagadamma KC. Physiotherapists with musculoskeletal training in an emergency department for patients with non-specific low back pain: a service evaluation. Musculoskelet Care. 2022;20(4):960–3. 10.1002/msc.1640 35491528

[92] Powell N. Physiotherapy in Mount Hagen General Hospital: an audit of activity over a six-month period. P N G Med J. 2001;44(1–2):24–35. 12418675

[93] Welford A, McCallum GB, Hodson M, Johnston H. Physiotherapy management of first nations children with bronchiectasis from remote top end communities of the northern territory: a retrospective chart audit. Front Pediatr. 2023;11. Available from: 10.3389/fped.2023.1230474/full. Cited 2024 Sep 25. 10.3389/fped.2023.1230474 PMC10613054 37900672

[94] McGlinchey MP, Paley L, Hoffman A, Douiri A, Rudd AG. Physiotherapy provision to hospitalised stroke patients: analysis from the UK Sentinel Stroke National Audit Programme. Eur Stroke J. 2019;4(1):75–84. 31165097 10.1177/2396987318800543 PMC6533865

[95] Nunn N. Practical challenges and limitations using the Oswestry disability low back Pain Questionnaire in a private practice setting in New Zealand. A clinical audit. New Z J Physiotherapy. 2012;40(1):24–8.

[96] Pidani AS, Ahmad T, Panjwani N, Noordin S. Prevention of falls in hospital: audit report from a tertiary care hospital of Pakistan. J Pak Med Assoc. 2021;71(8):S79–82. 34634022

[97] Singh G, McNamee G, Sharpe L, Lucas M, Lewis P, Newton C, et al. Psychological, social and lifestyle screening of people with low back pain treated by physiotherapists in a National Health Service musculoskeletal service: an audit. Eur J Physiotherapy. 2023;25(1):20–6.

[98] Kirby ED, Jones CB, Fickling SD, Pawlowski G, Brodie SM, Boyd LA et al. Real world evidence of improved attention and cognition during physical therapy paired with neuromodulation: a brain vital signs study. Front Hum Neurosci. 2023;17. Available from: 10.3389/fnhum.2023.1209480/full. Cited 2024 Sep 25. 10.3389/fnhum.2023.1209480 PMC10289164 37362950

[99] Dobson C. Record audit: a study of the quality and effectiveness of the treatment of knee conditions. Physiotherapy. 1995;81(4):217–21.

[100] Gorgon E, Maka K, Sullivan J, Nisbet G, Hancock M, Regan G, et al. Redesigning care for back pain in an Australian hospital setting: a service evaluation to identify need for change. Musculoskelet Care. 2023;21(1):232–43. 10.1002/msc.1695 36069172

[101] Lutz AD, Brooks JM, Chapman CG, Shanley E, Stout CE, Thigpen CA. Risk Adjustment of the modified low back Pain Disability Questionnaire and Neck Disability Index to Benchmark Physical Therapist performance: analysis from an outcomes Registry. Phys Ther. 2020;100(4):609–20. 32285130 10.1093/ptj/pzaa019

[102] Slattery B, Ackerman L, Jagadamma KC. Service evaluation of telehealth in a physiotherapy musculoskeletal setting: patient outcomes and results from risk stratification. Musculoskelet Care. 2022;20(4):977–90. 10.1002/msc.1623 35220671

[103] Turner P, Whitfield A, Brewster S, Halligan M, Kennedy J. The assessment of pain: an audit of physiotherapy practice. Australian J Physiotherapy. 1996;42(1):55–62. 10.1016/s0004-9514(14)60441-4 11676636

[104] Bateman M, McClymont S, Hinchliffe SR. The effectiveness and cost of corticosteroid injection and physiotherapy in the treatment of frozen shoulder—a single-centre service evaluation. Clin Rheumatol. 2014;33(7):1005–8. 24487485 10.1007/s10067-014-2501-x

[105] Bryant M, Gough A, Selfe J, Richards J, Burgess E. The effectiveness of ultrasound guided hydrodistension and physiotherapy in the treatment of frozen shoulder/adhesive capsulitis in primary care: a single centre service evaluation. Shoulder Elb. 2017;9(4):292–8. 10.1177/1758573217701063 PMC5598823 28932287

[106] Pearse E, Maclean A, Ricketts D. The Extended Scope Physiotherapist in Orthopaedic Out-patients – an audit. Ann R Coll Surg Engl. 2006;88(7):653–5. 17132315 10.1308/003588406X149183 PMC1963801

[107] Whelan G, Yeowell G, Littlewood C. The impact of introducing hydrodistension as a treatment for frozen shoulder in a primary care musculoskeletal service: a retrospective audit. Musculoskelet Care. 2023;21(3):953–7. 10.1002/msc.1736 36694385

[108] Goubar A, Ayis S, Beaupre L, Cameron ID, Milton-Cole R, Gregson CL, et al. The impact of the frequency, duration and type of physiotherapy on discharge after hip fracture surgery: a secondary analysis of UK national linked audit data. Osteoporos Int. 2022;33(4):839–50. 34748023 10.1007/s00198-021-06195-9 PMC8930962

[109] DENNINGER TR, COOK CE, CHAPMAN CG, MCHENRY T, THIGPEN CA. The influence of patient choice of first provider on costs and outcomes: analysis from a physical therapy patient Registry. J Orthop Sports Phys Therapy. 2018;48(2):63–71. 10.2519/jospt.2018.7423 29073842

[110] Goodwin V, Martin FC, Husk J, Lowe D, Grant R, Potter J. The national clinical audit of falls and bone health-secondary prevention of falls and fractures: a physiotherapy perspective. Physiotherapy. 2010;96(1):38–43. 20113761 10.1016/j.physio.2009.07.003

[111] Gates LS, Cherry L, Grønne DT, Roos EM, Skou ST. The prevalence of foot pain and association with baseline characteristics in people participating in education and supervised exercise for knee or hip osteoarthritis: a cross-sectional study of 26,003 participants from the GLA:D^®^ registry. J Foot Ankle Res. 2023;16(1):83. 37993923 10.1186/s13047-023-00673-5 PMC10666392

[112] Wilde JT. The UK Haemophilia Doctors Organisation triennial audit of UK Comprehensive Care Haemophilia centres. Haemophilia. 2012;18(4):491–5. 22564196 10.1111/j.1365-2516.2012.02817.x

[113] Sparkes V. Treatment of low back pain: monitoring clinical practice through audit. Physiotherapy. 2005;91(3):171–7.

[114] AC L, O H. TL V. Treatment patterns of multiple sclerosis patients: a comparison of veterans and non-veterans using the NARCOMS registry. Multiple sclerosis (Houndmills, Basingstoke, England). 2005;11(1):33–40. 10.1191/1352458505ms1136oa 15732264

[115] Benn R, Rawson L, Phillips A. Utilising a non-surgical intervention in the knee osteoarthritis care pathway: a 6-year retrospective audit on NHS patients. Therapeutic Adv Musculoskelet. 2023;15:1759720X231187190. 10.1177/1759720X231187190 PMC10387773 37529330

[116] Wood L, Eveleigh C, Dixon M, Dunstan E, Salem K. Was the impact of COVID-19 on a spinal triage service as significant as expected? A retrospective service evaluation: results and evaluation. Musculoskelet Care. 2022;20(3):697–704. 10.1002/msc.1680 PMC9539363 35962526

[117] Smyth C, Smart K, Fitzpatrick M, Caffrey A, McLoughlin C, Doody C. Physiotherapist-led triage of patients with thoracic spine pain in a musculoskeletal assessment clinic: a service evaluation of activity and outcomes. Physiotherapy Pract Res. 2019;40(2):145–53.

[118] Smyth N, Buckman JEJ, Naqvi SA, Aguirre E, Cardoso A, Pilling S, et al. Understanding differences in mental health service use by men: an intersectional analysis of routine data. Soc Psychiatry Psychiatr Epidemiol. 2022;57(10):2065–77. 35318495 10.1007/s00127-022-02256-4 PMC9477949

[119] Kaptan SK, Dernedde C, Dowden T, Akan A. Without it, I am not sure I would still be here’: a mixed methods service evaluation for online EMDR trauma therapy in a primary care network in England. Front Psychiatry. 2023;14:1301540. 38090696 10.3389/fpsyt.2023.1301540 PMC10714000

[120] Clarke J, Hyde A, Caswell RJ. A service evaluation of current practices in the assessment of mental-health and referral for support following disclosure of sexual violence. Int J STD AIDS. 2023;34(1):62–6. 36287485 10.1177/09564624221135295 PMC9806461

[121] McGowan NM, Syam N, McKenna D, Pearce S, Saunders KEA. A service evaluation of short-term mentalisation based treatment for personality disorder. BJPsych Open. 2021;7(5):e140. 34334153 10.1192/bjo.2021.974 PMC8358973

[122] Robinson A, De Boos D, Moghaddam N. A service evaluation of the assessment process in a Step4 psychological therapies Service. Mental Health Rev J. 2023;28(2):167–79.

[123] K Y. An audit of obsessive compulsive disorder in a Bedford (UK) Community Mental Health Team. Psychiatria Danubina. 2014;26:231–9. 25413546

[124] da Costa E, Koyee P, Bogdan N, Qassem T. An audit of the management of depression in a community population with intellectual disabilities in accordance with NICE guidelines. Br J Dev Disabil. 2011;57(Part 2113):147–57.

[125] Sumner J, Böhnke JR, Doherty P. Does service timing matter for psychological outcomes in cardiac rehabilitation? Insights from the National Audit of Cardiac Rehabilitation. Eur J Prev Cardiol. 2018;25(1):19–28. 29120237 10.1177/2047487317740951 PMC5757407

[126] Verbist IL, Fabian H, Huey D, Brooks H, Lovell K, Blakemore A. Exploring access and engagement with improving Access to Psychological therapies (IAPT) services, before, during, and after the COVID-19 lockdown: a service evaluation in the Northwest of England. Psychother Res. 2024;34(2):216–27. 36878217 10.1080/10503307.2023.2184285

[127] Carlin E, Cox Z, Orazi K, Derry KL, Dudgeon P. Exploring Mental Health Presentations in Remote Aboriginal Community Controlled Health Services in the Kimberley Region of Western Australia Using an Audit and File Reviews. Int J Environ Res Public Health. 2022;19(3). Available from: https://pubmed.ncbi.nlm.nih.gov/35162765/. Cited 2AD Jan 1. 10.3390/ijerph19031743 PMC8835535 35162765

[128] Halacre M, Jalil R. Holistic therapy with disabled adults from a social and individual perspective: a service evaluation feasibility study. Counselling Psychother Res. 2017;17(4):320–9.

[129] Hales SA, Di Simplicio M, Iyadurai L, Blackwell SE, Young K, Fairburn CG, et al. Imagery-focused cognitive therapy (ImCT) for Mood instability and anxiety in a small sample of patients with bipolar disorder: a pilot clinical audit. Behav Cogn Psychother. 2018;46(6):706–25. 29983124 10.1017/S1352465818000334 PMC6140996

[130] Kwon JY, Kopec J, Sutherland JM, Lambert LK, Anis AH, Sawatzky R. Patient-reported mental health and well-being trajectories in oncology patients during radiation therapy: an exploratory retrospective cohort analysis using the Ontario Cancer Registry. Qual Life Res. 2023;32(10):2899–909. 37140774 10.1007/s11136-023-03430-0

[131] Taylor PJ, Fien K, Mulholland H, Duarte R, Dickson JM, Kullu C. Pilot service evaluation of a brief psychological therapy for self-harm in an emergency department: hospital outpatient psychotherapy engagement service. Psychol Psychother. 2021;94:64–78. 32338445 10.1111/papt.12277

[132] Middleton E, Agius M, Zaman R. Post-traumatic stress disorder (PTSD) treatment experience in Bedford East - audit and reaudit. Psychiatr Danub. 2011;23(Suppl 1):S104–109. 21894114

[133] Davis A, Smith T, Talbot J, Eldridge C, Betts D. Predicting patient engagement in IAPT services: a statistical analysis of electronic health records. Evid Based Ment Health. 2020;23(1):8–14. 32046987 10.1136/ebmental-2019-300133 PMC7034348

[134] Baldwin DS, Dang M, Farquharson L, Fitzpatrick N, Lindsay N, Quirk A et al. Quality of English inpatient mental health services for people with anxiety or depressive disorders: findings and recommendations from the core audit of the National Clinical audit of anxiety and depression. Compr Psychiatr. 2021;104:N.PAG-N.PAG. 10.1016/j.comppsych.2020.152212 33160123

[135] Munasinghe S, Page A, Mannan H, Ferdousi S, Peek B. Referral patterns to primary mental health services in Western Sydney (Australia): an analysis of routinely collected data (2005–2018). Int J Mental Health Syst. 2020;14(1):37. 10.1186/s13033-020-00368-5 PMC7249634 32508982

[136] Clarke AM, McLaughlin P, Staunton J, Kerins K, Power B, Kearney K, et al. Retrospective study of first episode psychosis in the Dublin Southwest Mental Health Service: demographics, clinical profile and service evaluation of treatment. Ir J Psychol Med. 2019;36(4):249–58. 31747988 10.1017/ipm.2017.46

[137] Burdett H, Greenberg N. Service evaluation of a human Givens Therapy service for veterans. Occup Med. 2019;69(8–9):586–92. 10.1093/occmed/kqz045 31120512

[138] Lesage A, Séguin M, Guy A, Daigle F, Bayle MN, Chawky N, et al. Systematic Services Audit of Consecutive Suicides in New Brunswick: the case for coordinating specialist Mental Health and Addiction services. Can J Psychiatry. 2008;53(10):671–8. 18940035 10.1177/070674370805301006

[139] Conrad AM, Lewin TJ, Sly KA, Schall U, Halpin SA, Hunter M, et al. Ten-year audit of clients presenting to a specialised service for young people experiencing or at increased risk for psychosis. BMC Psychiatry. 2014;14:318. 25403891 10.1186/s12888-014-0318-4 PMC4239381

[140] Williamson E, Pipeva A, Brodrick A, Saradjian A, Slade P. The birth trauma psychological therapy service: an audit of outcomes. Midwifery. 2021;102:N.PAG-N.PAG. 10.1016/j.midw.2021.103099 34293486

[141] Pybis J, Saxon D, Hill A, Barkham M. The comparative effectiveness and efficiency of cognitive behaviour therapy and generic counselling in the treatment of depression: evidence from the 2nd UK National Audit of psychological therapies. BMC Psychiatry. 2017;17(1):215. 28599621 10.1186/s12888-017-1370-7 PMC5466727

[142] Zala D, Kartha MR, McCrone P, Brabban A, Stirzaker A. The cost-effectiveness of the improving Access to Psychological therapies (IAPT) Programme in severe Mental illness: a decision Analytical Model using Routine Data. Commun Ment Health J. 2019;55(5):873–83. 10.1007/s10597-019-00390-z 30848414

[143] Barkham M, Saxon D. The effectiveness of high-intensity CBT and counselling alone and following low-intensity CBT: a reanalysis of the 2nd UK National Audit of Psychological Therapies data. BMC Psychiatry. 2018;18(1):321. 30285674 10.1186/s12888-018-1899-0 PMC6171289

[144] Shepherd M, Butler L. The underuse of couple therapy for depression in improving Access to Psychological therapies Services (IAPTS): a service evaluation exploring its effectiveness and discussion of systemic barriers to its implementation. J Family Therapy. 2021;43(4):493–515.

[145] Robjant K, Roberts J, Katona C. Treating posttraumatic stress disorder in female victims of trafficking using narrative exposure therapy: a retrospective audit. Front Psychiatry. 2017;8:63. 28620321 10.3389/fpsyt.2017.00063 PMC5451503

[146] Spalding WM, Bertoia ML, Bulik CM, Seeger JD. Treatment characteristics among patients with binge-eating disorder: an electronic health records analysis. Postgrad Med. 2023;135(3):254–64. 35037815 10.1080/00325481.2021.2018255

[147] Polley MJ, Jolliffe R, Boxell E, Zollman C, Jackson S, Seers H. Using a whole person Approach to support people with Cancer: a longitudinal, mixed-methods service evaluation. Integr Cancer Ther. 2016;15(4):435–45. 27060342 10.1177/1534735416632060 PMC5739159

[148] Swan KL, Keene T, Avard BJ. A 12-Month clinical audit comparing point-of-care lactate measurements tested by paramedics with In-Hospital serum lactate measurements. Prehosp Disaster Med. 2018;33(1):36–42. 29293078 10.1017/S1049023X17007130

[149] Greene A, Vu EN, Archer T, Norman S, Trojanowski J, Shih AW. A service evaluation of Prehospital Blood transfusion by critical care paramedics in British Columbia, Canada. Air Med J. 2021;40(6):441–5. 34794786 10.1016/j.amj.2021.07.004

[150] Eliakundu AL, Cadilhac DA, Kim J, Kilkenny MF, Bagot KL, Andrew E, et al. Determining the sensitivity of emergency dispatcher and paramedic diagnosis of stroke: statewide registry linkage study. J Am Coll Emerg Physicians Open. 2022;3(4):e12750. 35795711 10.1002/emp2.12750 PMC9249375

[151] Pilbery R, Teare MD, Lawton D. Do RATs save lives? A service evaluation of an out-of-hospital cardiac arrest team in an English ambulance service. Br Paramed J. 2019;3(4):32–9. 33328815 10.29045/14784726.2019.03.3.4.32 PMC7706746

[152] Bieler D, Franke A, Lefering R, Hentsch S, Willms A, Kulla M, et al. Does the presence of an emergency physician influence pre-hospital time, pre-hospital interventions and the mortality of severely injured patients? A matched-pair analysis based on the trauma registry of the German Trauma Society (TraumaRegister DGU^®^). Injury. 2017;48(1):32–40. 27586065 10.1016/j.injury.2016.08.015

[153] Houghton Budd S, Alexander-Elborough E, Brandon R, Fudge C, Hardy S, Hopkins L, et al. Drug-free tracheal intubation by specialist paramedics (critical care) in a United Kingdom ambulance service: a service evaluation. BMC Emerg Med. 2021;21(1):144. 34800983 10.1186/s12873-021-00533-0 PMC8605587

[154] Katayama Y, Kitamura T, Kiyohara K, Ishida K, Hirose T, Nakao S, et al. Effect of fluid administration on scene to traffic accident patients by EMS personnel: a propensity score-matched study using population-based ambulance records and nationwide trauma registry in Japan. Eur J Trauma Emerg Surg. 2022;48(2):999–1007. 33492423 10.1007/s00068-020-01590-z PMC9001559

[155] Al-Shaqsi S, Al-Kashmiri A, Al-Hajri H, Al-Harthy A. Emergency medical services versus private transport of trauma patients in the Sultanate of Oman: a retrospective audit at the Sultan Qaboos University Hospital. Emerg Med J. 2014;31(9):754–7. 23825061 10.1136/emermed-2013-202779

[156] Nichols M, Fouche PF, McPherson T, Evens T, Bendall J. Lessons from the first two years of a new out-of-hospital airway registry in New South Wales. Paramedicine. 2023;20(5):152–60.

[157] Deasy C, Hall D, Bray JE, Smith K, Bernard SA, Cameron P. Paediatric out-of-hospital cardiac arrests in Melbourne, Australia: improved reporting by adding coronial data to a cardiac arrest registry. Emerg Med J (EMJ). 2013;30(9):740–4. 23038692 10.1136/emermed-2012-201531

[158] Sinclair JE, Austin MA, Leduc S, Dionne R, Froats M, Marchand J, et al. Patient and Prehospital Predictors of Hospital Admission for patients with and without histories of Diabetes treated by paramedics for hypoglycemia: a Health Record Review study. Prehospital Emerg Care. 2023;27(7):955–66. 10.1080/10903127.2022.2137863 36264569

[159] Endo A, Kojima M, Uchiyama S, Shiraishi A, Otomo Y. Physician-led prehospital management is associated with reduced mortality in severe blunt trauma patients: a retrospective analysis of the Japanese nationwide trauma registry. Scand J Trauma Resusc Emerg Med. 2021;29(1):9. 33407748 10.1186/s13049-020-00828-4 PMC7789566

[160] Bossers SM, Verheul R, van Zwet EW, Bloemers FW, Giannakopoulos GF, Loer SA, et al. Prehospital Intubation of patients with severe traumatic brain Injury: a Dutch Nationwide Trauma Registry Analysis. Prehospital Emerg Care. 2023;27(5):662–8. 10.1080/10903127.2022.2119494 36074561

[161] Murad MK, Larsen S, Husum H. Prehospital trauma care reduces mortality. Ten-year results from a time-cohort and trauma audit study in Iraq. Scand J Trauma Resusc Emerg Med. 2012;20:13. 22304808 10.1186/1757-7241-20-13 PMC3298775

[162] MacCallum AG, Stafford PJ, Jones C, Vincent R, Perez-Avila C, Chamberlain DA. Reduction in hospital time to thrombolytic therapy by audit of policy guidelines. Eur Heart J. 1990;11(Suppl F):48–52. 2226541 10.1093/eurheartj/11.suppl_f.48

[163] Metcalf M, Robinson M, Hall P, Goss J. The Cardiac arrest support tier: a service evaluation. Br Paramed J. 2020;5(2):38–47. 33456390 10.29045/14784726.2020.09.5.2.38 PMC7783951

[164] Nichols M, Fouche PF, Bendall JC. Video versus direct laryngoscopy by specialist paramedics in New South Wales: preliminary results from a new airway registry. Emerg Med Australasia. 2022;34(6):984–8. 10.1111/1742-6723.14033 35717028

[165] Freeman-Sanderson A, Togher L, Phipps P, Elkins M. A clinical audit of the management of patients with a tracheostomy in an Australian tertiary hospital intensive care unit: focus on speech-language pathology. Int J Speech Lang Pathol. 2011;13(6):518–25. 21936760 10.3109/17549507.2011.582520

[166] Britton L, Albery L, Bowden M, Harding-Bell A, Phippen G, Sell D. A cross-sectional cohort study of Speech in five-year-Olds with Cleft Palate ± Lip to Support Development of National Audit standards. Cleft Palate Craniofac J. 2014;51(4):431–51. 24635034 10.1597/13-121

[167] Chalmers S, Harrall K, Wong SY, Kablan W, Clunie G. A retrospective study of patients presenting with speech and language therapy needs within multidisciplinary Long COVID services: A service evaluation describing and comparing two cohorts across two NHS Trusts. International Journal of Language & Communication Disorders. 2022;n/a(n/a). Available from: 10.1111/1460-6984.12868. Cited 2023 Jul 27. 10.1111/1460-6984.12868 36916685

[168] James NK, Twist M, Milward TM, Turner MM. An audit of velopharyngeal incompetence treated by the orticochea pharyngoplasty. Br J Plast Surg. 1996;49(4):197–201. 8757666 10.1016/s0007-1226(96)90050-8

[169] Rona RJ, Reynolds A, Allsop M, Morris RW, Morgan M, Mandalia S. Audit from preschool developmental surveillance of vision, hearing, and language referrals. Arch Dis Child. 1991;66(8):921–6. 1929488 10.1136/adc.66.8.921 PMC1793450

[170] Blyth KM, McCabe P, Heard R, Clark J, Madill C, Ballard KJ. Cancers of the Tongue and Floor of Mouth: five-year file audit within the Acute Phase. Am J Speech-Language Pathol. 2014;23(4):668–78. 10.1044/2014_AJSLP-14-0003 25089517

[171] Messing BP, Ward EC, Lazarus C, Ryniak K, Kim M, Silinonte J, et al. Establishing a Multidisciplinary Head and Neck Clinical Pathway: an Implementation Evaluation and Audit of Dysphagia-Related Services and outcomes. Dysphagia (0179051X). 2019;34(1):89–104. 10.1007/s00455-018-9917-4 PMC6349813 29922848

[172] Wight S, Miller N. Lee Silverman Voice Treatment for people with Parkinson’s: audit of outcomes in a routine clinic. Int J Lang Commun Disord. 2015;50(2):215–25. 25469736 10.1111/1460-6984.12132

[173] Inman DS, Thomas P, Hodgkinson PD, Reid CA. Oro-nasal fistula development and velopharyngeal insufficiency following primary cleft palate surgery—an audit of 148 children born between 1985 and 1997. Br J Plast Surg. 2005;58(8):1051–4. 16084930 10.1016/j.bjps.2005.05.019

[174] Stansfield J. Parents with learning disabilities and speech and language therapy. A service evaluation of referrals and episodes of care. Br J Learn Disabil. 2012;40(3):169–76.

[175] Sullivan R, Hemsley B, Harding K, Skinner I. Patient unable to express why he was on the floor, he has aphasia.’ A content thematic analysis of medical records and incident reports on the falls of hospital patients with communication disability following stroke. Int J Lang Communication Disorders. 2023;58(6):2033–48. 10.1111/1460-6984.12916 37355936

[176] Raol N, Lilley E, Cooper Z, Dowdall J, Morris MA. Preoperative counseling in Salvage Total Laryngectomy: Content Analysis of Electronic Medical Records. Otolaryngology-Head Neck Surg. 2017;157(4):641–7. 10.1177/0194599817726769 28828922

[177] Morris MA, Kho AN. Silence in the EHR: infrequent documentation of aphonia in the electronic health record. BMC Health Serv Res. 2014;14(1):425–425. 25248751 10.1186/1472-6963-14-425 PMC4181429

[178] Loundon N, Simon F, Aubry K, Bordure P, Bozorg-Grayeli A, Deguine O, et al. The French Cochlear Implant Registry (EPIIC): perception and language results in infants with cochlear implantation under the age of 24 months. Eur Ann Otorhinolaryngol Head Neck Dis. 2020;137:S11-8. 32863156 10.1016/j.anorl.2020.07.010

[179] McCrory E. Voice Therapy Outcomes in Vocal fold nodules: a retrospective audit. Int J Lang Communication Disorders. 2001;36(S1):19–24. 10.3109/13682820109177852 11340780

[180] Miller J, Hopkinson C. A retrospective audit exploring the use of relaxation as an intervention in oncology and palliative care. Eur J Cancer Care. 2008;17(5):488–91. 10.1111/j.1365-2354.2007.00899.x 18637113

[181] Krüger C, van der Westhuizen R. An audit of attendance at occupational therapy by long-term psychiatric in-patients at Weskoppies Hospital. South Afr J Occup Therapy. 2011;41(2):2–7.

[182] Green A. An audit of Occupational Therapy Outpatient attendance. Br J Occup Therapy. 1997;9(1/1997):384–8.

[183] Smith T, Rees V. An audit of referrals to Occupational Therapy for older adults attending an Accident and Emergency Department. Br J Occup Therapy. 2004;67(4):153–8.

[184] Rahja M, Comans T, Clemson L, Crotty M, Laver K. Are there missed opportunities for occupational therapy for people with dementia? An audit of practice in Australia. Aust Occup Ther J. 2018;65(6):565–74. 30187917 10.1111/1440-1630.12514

[185] McIntyre A. Elderly fallers: a baseline audit of admissions to a Day Hospital for Elderly people. Br J Occup Therapy. 1999;62(6):244–8.

[186] Stewart K, Hancock N, Stancliffe RJ. Factors related to hospital utilisation for people living with schizophrenia: examining Allen’s cognitive level scores, recommended supports and routinely collected variables. Aust Occup Ther J. 2019;66(5):591–602. 31342528 10.1111/1440-1630.12597

[187] Brewin J, Hazell A. How successful are we at getting our clients back to work? The results of an audit. Br J Occup Therapy. 2004;4(1/2004):148–52.

[188] Marnane K, Gustafsson L, Liddle J, Molineux M. Interventions for driving disruption in Community Rehabilitation: a Chart audit. Disabil Rehabilitation. 2023;45(26):4424–30. 10.1080/09638288.2022.2152501 36448310

[189] M L, H J. Occupational therapy and vocational rehabilitation: an audit of an outpatient occupational therapy service. Br J Occup Therapy. 2006;69(6):288–92.

[190] Foster HE, Pyle C, Walker DJ. Provision of medical and community services to people with severe arthritis; an audit. Br J Rheumatol. 1991;30(5):356–60. 1833021 10.1093/rheumatology/30.5.356

[191] Kutsuna I, Hoshino A, Morisugi A, Mori Y, Shirato A, Takeda M, et al. Relationship between emotional words in electronic medical records and leave periods of users of a return-to-work program with depression. Br J Occup Therapy. 2022;85(12):993–1001. 10.1177/03080226221107773 PMC12033768 40337345

[192] Davies N, Simelane B. The impact of seven-day working for patients and staff in an acute physical older adults unit: a service evaluation. Br J Occup Therapy. 2017;80(11):689–93.

[193] O’Reilly S, Strong J, Ziviani J, Brown J, McAuliffe T. The role of the Outpatient Occupational Therapist treating patients with small Burns: a retrospective audit of practice. J Burn Care Res. 2023;44(1):87–94. 36018792 10.1093/jbcr/irac123

[194] Clay F, Hunt R, Obiefuna N, Solly JE, Watson E, Wilkinson A, et al. The Use of Immersive virtual reality in sensory Sessions on a specialist dementia unit: service evaluation of feasibility and acceptability. Occup Therapy Health Care. 2024;38(2):317–30. 10.1080/07380577.2023.2270052 37933866

[195] Barry K, Kumar S, Linke R, Dawes E. A clinical audit of anatomical side marker use in a paediatric medical imaging department. J Med Radiat Sci. 2016;63(3):148–54. 27648278 10.1002/jmrs.176 PMC5016612

[196] Rodrigues JCL, Negus IS, Manghat NE, Hamilton MCK. A completed audit cycle of the lateral scan projection radiograph in CT pulmonary angiography (CTPA); the impact on scan length and radiation dose. Clin Radiol. 2013;68(6):574–9. 23541095 10.1016/j.crad.2012.11.016

[197] Karthik S, O’Regan DJ. An audit of follow-up chest radiography after coronary artery bypass graft. Clin Radiol. 2006;61(7):616–8. 16784948 10.1016/j.crad.2006.04.002

[198] Eze KC, Omodia N, Okegbunam B, Adewonyi T, Nzotta CC. An audit of rejected repeated x-ray films as a quality assurance element in a radiology department. Niger J Clin Pract. 2008;11(4):355–8. 19320410

[199] Henderson D, Gray WK, Booth L. Assessment of a reporting radiographer-led discharge system for minor injuries: a prospective audit over 2 years. Emerg Med J (EMJ). 2013;30(4):298–302. 22535693 10.1136/emermed-2011-200642

[200] Afolabi OA, Fadare JO, Essien EM. Audit of completion of radiology request form in a Nigerian specialist hospital. Ann Ib Postgrad Med. 2012;10(2):48–52. 25161413 PMC4111050

[201] Mukerji N, Wallace D, Mitra D. Audit of the change in the on-call practices in neuroradiology and factors affecting it. BMC Med Imaging. 2006;6(1):13. 17042951 10.1186/1471-2342-6-13 PMC1622746

[202] AB, PL, RS, TI. DIAGNOSTIC IMAGING AND IONIZING RADIATION EXPOSURE IN A LEVEL 1 TRAUMA CENTRE POPULATION MET WITH TRAUMA TEAM ACTIVATION: A ONE-YEAR PATIENT RECORD AUDIT. Radiat Prot Dosimetry. 2020;189(1):35–47. 10.1093/rpd/ncaa010 32060518

[203] Inal T, Atac G, İnal T, Ataç G. Dose audit for patients undergoing two common radiography examinations with digital radiology systems. Diagn Interventional Radiol. 2014;20(1):100–4. 10.5152/dir.2013.12122 PMC4463244 24317331

[204] McCarty M, Waugh R, McCallum H, Montgomery RJ, Aszkenasy OM. Paediatric pelvic imaging: improvement in gonad shield placement by multidisciplinary audit. Pediatr Radiol. 2001;31(9):646–9. 11512007 10.1007/s002470100515

[205] Johnson K. Reducing unnecessary skull radiographs in children: a multidisciplinary audit. Clin Radiol. 2004;59(7):616–20. 15208068 10.1016/j.crad.2003.11.019

[206] Priest JR, Williams GM, Mize WA, Dehner LP, McDermott MB. Nasal chondromesenchymal hamartoma in children with pleuropulmonary blastoma—A report from the International Pleuropulmonary Blastoma Registry registry. Int J Pediatr Otorhinolaryngol. 2010;74(11):1240–4. 20822816 10.1016/j.ijporl.2010.07.022

[207] Reuter B, Gumbinger C, Sauer T, Wiethölter H, Bruder I, Diehm C, et al. Access, timing and frequency of very early stroke rehabilitation - insights from the Baden-Wuerttemberg stroke registry. BMC Neurol. 2016;16:222–222. 27852229 10.1186/s12883-016-0744-7 PMC5112693

[208] Lamassa M, Di Carlo A, Pracucci G, Basile AM, Trefoloni G, Vanni P, et al. Characteristics, outcome, and care of stroke associated with atrial fibrillation in Europe: data from a multicenter multinational hospital-based registry (The European Community Stroke Project). Stroke. 2001;32(2):392–8. 11157172 10.1161/01.str.32.2.392

[209] Gittins M, Vail A, Bowen A, Lugo-Palacios D, Paley L, Bray B, et al. Factors influencing the amount of therapy received during inpatient stroke care: an analysis of data from the UK Sentinel Stroke National Audit Programme. Clin Rehabil. 2020;34(7):981–91. 32508132 10.1177/0269215520927454 PMC7324910

[210] Gittins M, Lugo-Palacios DG, Paley L, Bray B, Bowen A, Vail A, et al. How do patients pass through stroke services? Identifying stroke care pathways using national audit data. Clin Rehabil. 2020;34(5):698–709. 32141324 10.1177/0269215520907654 PMC7443957

[211] Li S, Lu Y, Fang S, Wang L, Peng B. Inpatient rehabilitation therapy in stroke patients with reperfusion therapy: a national prospective registry study. BMC Neurol. 2023;23(1). 10.1186/s12883-023-03144-3 PMC10073784 37020194

[212] Gittins M, Lugo-Palacios DG, Vail A, Bowen A, Paley L, Bray B, et al. Investigating the association between inpatient stroke therapy and disability, destination on discharge, length of stay and mortality: a prospective cohort study using the Sentinel Stroke National Audit Programme. BMJ Open. 2022;12(4):e059684. 35365545 10.1136/bmjopen-2021-059684 PMC8977818

[213] Kuptniratsaikul V, Kovindha A, Suethanapornkul S, Manimmanakorn N, Archongka Y. Long-term morbidities in stroke survivors: a prospective multicenter study of Thai stroke rehabilitation registry. BMC Geriatr. 2013;13(1):33–33. 23586971 10.1186/1471-2318-13-33 PMC3635998

[214] Rudd AG, Lowe D, Irwin P, Rutledge Z, Pearson M. Intercollegiate Stroke Working Party. National stroke audit: a tool for change? Qual Health Care. 2001;10(3):141–51. 11533421 10.1136/qhc.0100141.. PMC1743440

[215] Faluyi OO, Omodara JA, Tay KH, Muhiddin K. Retrospective audit of the acute management of stroke in two district general hospitals in the Uk. Ann Ib Postgrad Med. 2008;6(1):42–8. 25161444 10.4314/aipm.v6i1.64039 PMC4111017

[216] Lobo Chaves MA, Gittins M, Bray B, Vail A, Smith CJ. The Timing of Stroke Care Processes and Development of Stroke Associated Pneumonia: A National Registry Cohort Study. Front Neurol. 2022;13. Available from: 10.3389/fneur.2022.875893/full. Cited 2024 Sep 30. 10.3389/fneur.2022.875893 PMC9043446 35493828

[217] Bray BD, Cloud GC, James MA, Hemingway H, Paley L, Stewart K, et al. Weekly variation in health-care quality by day and time of admission: a nationwide, registry-based, prospective cohort study of acute stroke care. Lancet. 2016;388(10040):170–7. 27178477 10.1016/S0140-6736(16)30443-3

[218] Åkerblom S, Cervin M, Perrin S, Fischer MR, Gerdle B, McCracken LM. A Network Analysis of Clinical Variables in Chronic Pain: a study from the Swedish Quality Registry for Pain Rehabilitation (SQRP). Pain Med. 2021;22(7):1591–602. 33706371 10.1093/pm/pnaa473

[219] Boulton KA, Hodge A, Levu K, Ong N, Silove N, Guastella AJ. Access and barriers to supports for children and caregivers attending public child developmental assessment services: findings from the Sydney child neurodevelopment research registry. Autism Res. 2024;17(3):555–67. 38009266 10.1002/aur.3064

[220] Sakel M. An audit of botulinum toxin therapy services for adult spasticity. Int J Therapy Rehabilitation. 2008;15(1):15–21.

[221] Piette V, Deliens L, Debulpaep S, Cohen J, Beernaert K. Appropriateness of end-of-life care for children with genetic and congenital conditions: a cohort study using routinely collected linked data. Eur J Pediatr. 2023;182(9):3857–69. 37328636 10.1007/s00431-023-05030-z

[222] Azarbani N, Javadzadeh A, Mohseni I, Jalali A, Andalib E, Poormoghim H. Association of Musculoskeletal and Radiological Features with clinical and serological findings in systemic sclerosis: a single-centre Registry Study. Mediterr J Rheumatol. 2020;31(3):341–9. 33163868 10.31138/mjr.31.3.341 PMC7641026

[223] Bowen SJM, Thomson AH. British Thoracic Society Paediatric Pneumonia Audit: a review of 3 years of data. Thorax. 2013;68(7):682–3. 23291351 10.1136/thoraxjnl-2012-203026

[224] Hammond R, Lennon S, Walker MF, Hoffman A, Irwin P, Lowe D. Changing occupational therapy and physiotherapy practice through guidelines and audit in the United Kingdom. Clin Rehabil. 2005;19(4):365–71. 15929504 10.1191/0269215505cr784oa

[225] O’Regan E, Stadskleiv K, Czuba T, Alriksson-Schmidt AI. Cognitive assessments among children with cerebral palsy in Sweden and the use of augmentative and alternative communication and interpreters: a cross-sectional registry study. Disabil Rehabilitation. 2023;45(22):3656–67. 10.1080/09638288.2022.2138571 36308310

[226] Lewis D, Fullard K, Kolbe T, Chapman S, Divanoglou A, Doran C, et al. Does face-to-face pre-operative joint replacement education reduce hospital costs in a regional Australian hospital? A descriptive retrospective clinical audit. Eur J Orthop Surg Traumatol. 2020;30(2):257–65. 31612317 10.1007/s00590-019-02548-7

[227] Ohlmeier C, Saum KU, Galetzka W, Beier D, Gothe H. Epidemiology and health care utilization of patients suffering from Huntington’s disease in Germany: real world evidence based on German claims data. BMC Neurol. 2019;19(1):318. 31823737 10.1186/s12883-019-1556-3 PMC6905058

[228] Kalula SZ, de Villiers L, Ross K, Ferreira M. Management of older patients presenting after a fall–an accident and emergency department audit. S Afr Med J. 2006;96(8):718–21. 17019495

[229] Ringqvist Å, Dragioti E, Björk M, Larsson B, Gerdle B. Moderate and stable Pain reductions as a result of Interdisciplinary Pain Rehabilitation—A Cohort Study from the Swedish Quality Registry for Pain Rehabilitation (SQRP). J Clin Med. 2019;8(6):905. 31238588 10.3390/jcm8060905 PMC6617026

[230] Byrnes M, Beilby J, Ray P, McLennan R, Ker J, Schug S. Patient-focused goal planning process and outcome after spinal cord injury rehabilitation: quantitative and qualitative audit. Clin Rehabil. 2012;26(12):1141–9. 22653375 10.1177/0269215512442669

[231] Söderlund A, Löfgren M, Stålnacke BM, Söderlund A, Löfgren M, Stålnacke BM. Predictors before and after multimodal rehabilitation for pain acceptance and engagement in activities at a 1-year follow-up for patients with whiplash-associated disorders (WAD)-a study based on the Swedish Quality Registry for Pain Rehabilitation (SQRP). Spine J. 2018;18(8):1475–82. 29155342 10.1016/j.spinee.2017.11.014

[232] Lachiewicz AM, Stackhouse TM, Burgess K, Burgess D, Andrews HF, Choo TH et al. Sensory Symptoms and Signs of Hyperarousal in Individuals with Fragile X Syndrome: Findings from the FORWARD Registry and Database Multisite Study. J Autism Dev Disord. 2023. Available from: 10.1007/s10803-023-06135-y. Cited 2024 Jul 17. 10.1007/s10803-023-06135-y PMC11461590 37840096

[233] Hart O, Ryton BA. Sheffield spinal pathway audit cycle – pathways, mountains and the view from the top. Br J Pain. 2014;8(1):43–8. 26516533 10.1177/2049463713504387 PMC4590173

[234] Magon RK, Latheesh B, Müller U. Specialist adult ADHD clinics in East Anglia: service evaluation and audit of NICE guideline compliance†. BJPsych Bull. 2015;39(3):136–40. 26191453 10.1192/pb.bp.113.043257 PMC4478931

[235] Rubbo B, Saville C, Dall’Ora C, Turner L, Jones J, Ball J, et al. Staffing levels and hospital mortality in England: a national panel study using routinely collected data. BMJ Open. 2023;13(5):e066702. 37197808 10.1136/bmjopen-2022-066702 PMC10193053

[236] Barer Y, Ribalov R, Yaari A, Maor R, Arow Q, Logan J, et al. The clinical and economic Burden of Tardive Dyskinesia in Israel: real-World Data Analysis. J Clin Psychopharmacol. 2022;42(5):454. 36018237 10.1097/JCP.0000000000001597 PMC9426751

[237] Yanagi H, Terashi H, Takahashi Y, Okabe K, Tanaka K, Kimura C, et al. The Japanese registry for surgery of ischial pressure ulcers: STANDARDS-I. J Wound Care. 2020;29(Sup9a):S39–47. 10.12968/jowc.2018.27.3.174 29509114

[238] Milton-Cole R, O’Connell MDL, Sheehan KJ, Ayis S. The role of depression in the association between physiotherapy frequency and duration and outcomes after hip fracture surgery: secondary analysis of the physiotherapy hip fracture sprint audit. Eur Geriatr Med. 2023;14(5):999–1010. 37329488 10.1007/s41999-023-00808-8 PMC10587201

[239] Morphet J, Griffiths DL, Crawford K, Williams A, Jones T, Berry B, et al. Using transprofessional care in the emergency department to reduce patient admissions: a retrospective audit of medical histories. J Interprof Care. 2016;30(2):226–31. 26954260 10.3109/13561820.2015.1115394

[240] Fischer MR, Schults ML, Stålnacke BM, Ekholm J, Persson EB, Löfgren M. Variability in patient characteristics and service provision of interdisciplinary pain rehabilitation: a study using?the Swedish national quality registry for pain rehabilitation. J Rehabil Med. 2020;52(11):1–10. 10.2340/16501977-2765 33191437

[241] Cahill LS, Lannin NA, Purvis T, Cadilhac DA, Mak-Yuen Y, O’Connor DA, et al. What is ‘usual care’ in the rehabilitation of upper limb sensory loss after stroke? Results from a national audit and knowledge translation study. Disabil Rehabilitation. 2022;44(21):6462–70. 10.1080/09638288.2021.1964620 34498991

[242] Parashos SA, Bloem BR, Browner NM, Giladi N, Gurevich T, Hausdorff JM, et al. What predicts falls in Parkinson disease? Observations from the Parkinson’s Foundation registry. Neurol Clin Pract. 2018;8(3):214–22. 30105161 10.1212/CPJ.0000000000000461 PMC6075989

[243] Gerdle B, Åkerblom S, Brodda Jansen G, Enthoven P, Ernberg M, Dong HJ, et al. Who benefits from multimodal rehabilitation – an exploration of pain, psychological distress, and life impacts in over 35,000 chronic pain patients identified in the Swedish Quality Registry for Pain Rehabilitation. J Pain Res. 2019;12:891–908. 30881099 10.2147/JPR.S190003 PMC6411315

[244] Health and Care Professions Council. Registrant Snapshot – 4. October 2024. 2024 [cited 2024 Oct 25]. Registrant snapshot – 4 October 2024 |. Available from: https://www.hcpc-uk.org/resources/data/2024/registrant-snapshot-october-2024/.

[245] Turner KM, Huntley A, Yardley T, Dawson S, Dawson S. Defining usual care comparators when designing pragmatic trials of complex health interventions: a methodology review. Trials. 2024;25(1):117. 38342896 10.1186/s13063-024-07956-7 PMC10860249

[246] Whyte J, Hart T. It’s more than a Black Box; it’s a Russian Doll: defining Rehabilitation treatments. Am J Phys Med Rehabil. 2003;82(8):639. 12872021 10.1097/01.PHM.0000078200.61840.2D

[247] Bonnechère B. Unlocking the Black Box? A Comprehensive Exploration of large Language models in Rehabilitation. Am J Phys Med Rehabil. 2024;103(6):532. 38261757 10.1097/PHM.0000000000002440

[248] Sammani A, Jansen M, Linschoten M, Bagheri A, de Jonge N, Kirkels H, et al. UNRAVEL: big data analytics research data platform to improve care of patients with cardiomyopathies using routine electronic health records and standardised biobanking. Neth Heart J. 2019;27(9):426–34. 31134468 10.1007/s12471-019-1288-4 PMC6712144

[249] Ramani S, Whyle EB, Kagwanja N. What research evidence can support the decolonisation of global health? Making space for deeper scholarship in global health journals. Lancet Global Health. 2023;11(9):e1464–8. 37591593 10.1016/S2214-109X(23)00299-1

[250] Liu F, Demosthenes P. Real-world data: a brief review of the methods, applications, challenges and opportunities. BMC Med Res Methodol. 2022;22(1):287. 36335315 10.1186/s12874-022-01768-6 PMC9636688

[251] Rudrapatna VA, Butte AJ. Opportunities and challenges in using real-world data for health care. J Clin Invest. 2020;130(2):565–74. 32011317 10.1172/JCI129197 PMC6994109

[252] Harvey S, Stone M, Zingelman S, Copland DA, Kilkenny MF, Godecke E, et al. Comprehensive quality assessment for aphasia rehabilitation after stroke: protocol for a multicentre, mixed-methods study. BMJ Open. 2024;14(3):e080532. 38514146 10.1136/bmjopen-2023-080532 PMC10961567

[253] Agarwal A, Thirunarayan K, Romine WL, Alambo A, Cajita M, Banerjee T. Leveraging Natural Learning Processing to Uncover Themes in Clinical Notes of Patients Admitted for Heart Failure. In: 2022 44th Annual International Conference of the IEEE Engineering in Medicine & Biology Society (EMBC). 2022:2643–6. Available from: https://ieeexplore.ieee.org/abstract/document/9871400. Cited 2024 Oct 17. 10.1109/EMBC48229.2022.9871400 36085789

[254] Rust G, Cooper LA. How can practice-based Research Contribute to the elimination of Health disparities? J Am Board Fam Med. 2007;20(2):105–14. 17341746 10.3122/jabfm.2007.02.060131

[255] Langley J, Wolstenholme D, Cooke J. Collective making’ as knowledge mobilisation: the contribution of participatory design in the co-creation of knowledge in healthcare. BMC Health Serv Res. 2018;18(1):585. 30045726 10.1186/s12913-018-3397-y PMC6060522

[256] NHS England. Allied Health Professions’ Research and Innovation Strategy for England. 2022. Available from: https://www.hee.nhs.uk/our-work/allied-health-professions/enable-workforce/allied-health-professions%E2%80%99-research-innovation-strategy-england. Cited 2024 Oct 17.

[257] Crooke PJ, Olswang LB. Practice-based research: another pathway for closing the research–practice gap. J Speech Lang Hear Res. 2015;58(6):S1871–82. 26501941 10.1044/2015_JSLHR-L-15-0243

[258] Cordrey T, King E, Pilkington E, Gore K, Gustafson O. Exploring research capacity and culture of allied health professionals: a mixed methods evaluation. BMC Health Serv Res. 2022;22(1):85. 35039018 10.1186/s12913-022-07480-x PMC8764821

[259] Chalmers S, Hill J, Connell L, Ackerley SJ, Kulkarni AA, Roddam H. Allied health professional research engagement and impact on healthcare performance: a systematic review protocol. Int J Lang Communication Disorders. 2023;58(3):959–67. 10.1111/1460-6984.12812 36354267

[260] Harris J, Cooke J, Grafton K. Shaping Better Practice Through Research: A Practitioner Framework. 2019.

[261] Williams J, Craig TJ, Robson D. Barriers and facilitators of clinician and researcher collaborations: a qualitative study. BMC Health Serv Res. 2020;20(1):1126. 33278896 10.1186/s12913-020-05978-w PMC7718701

[262] Ciemins EL, Mollis BL, Brant JM, Hassell LA, Albritton S, Amoroso P, et al. Clinician engagement in research as a path toward the learning health system: a regional survey across the northwestern United States. Health Serv Manage Res. 2020;33(1):33–42. 31422696 10.1177/0951484819858830 PMC10729705

[263] Sackett DL. Evidence-based medicine. Semin Perinatol. 1997;21(1):3–5. 9190027 10.1016/s0146-0005(97)80013-4

[264] Schmitt-Egenolf M. The disruptive force of real-world evidence. J Clin Med. 2023;12(12):4026. 37373719 10.3390/jcm12124026 PMC10299663

[265] Norori N, Hu Q, Aellen FM, Faraci FD, Tzovara A. Addressing bias in big data and AI for health care: A call for open science. PATTER. 2021;2(10). Available from: https://www.cell.com/patterns/abstract/S2666-3899(21)00202-6. Cited 2024 Oct 18. 10.1016/j.patter.2021.100347 PMC8515002 34693373

[266] Straw I. The automation of bias in medical Artificial Intelligence (AI): decoding the past to create a better future. Artif Intell Med. 2020;110:101965. 33250145 10.1016/j.artmed.2020.101965

[267] Baumgartner R, Arora P, Bath C, Burljaev D, Ciereszko K, Custers B, et al. Fair and equitable AI in biomedical research and healthcare: social science perspectives. Artif Intell Med. 2023;144:102658. 37783540 10.1016/j.artmed.2023.102658

[268] Gillborn D, Warmington P, Demack S. QuantCrit: education, policy, ‘Big data’ and principles for a critical race theory of statistics. Race Ethn Educ. 2018;21(2):158–79.

[269] Gugnani N. Real world evidence: will the pyramid of evidence need some redefining…. Evid Based Dent. 2024;25(3):119–20. 38997511 10.1038/s41432-024-01035-1

[270] Khan SA. Decolonising global health by decolonising academic publishing. BMJ Global Health. 2022;7(3):e007811. 35301234 10.1136/bmjgh-2021-007811 PMC8931802

[271] Östlin P, Schrecker T, Sadana R, Bonnefoy J, Gilson L, Hertzman C, et al. Priorities for Research on Equity and Health: towards an Equity-Focused Health Research Agenda. PLoS Med. 2011;8(11):e1001115. 22069378 10.1371/journal.pmed.1001115 PMC3206017

