# Supplementary material for: Operationalising routinely collected patient data in research to further the pursuit of social justice and health equity: a team-based scoping review
Source: BMC Med Res Methodol. 2025 Jan 21;25:14. doi: 10.1186/s12874-025-02466-9 (PMC11749527; doi:10.1186/s12874-025-02466-9)
Supplement: Supplementary file 1 — Supplementary Material 1. [file 12874_2025_2466_MOESM1_ESM.docx]

| **Publication information** | | | | **Affiliation/s** | | **Study information** | | | | | | | | **Data information** | | |
| --- | --- | --- | --- | --- | --- | --- | --- | --- | --- | --- | --- | --- | --- | --- | --- | --- |
| **Title** | **Year** | **Journal** | **Authors** | **Lead author** | **Team** | **Country (first author)** | **Area** | **Dataset Size** | **Situated as filling a research-to-practice gap** | **Rationale** | **Aim** | **Setting** | **Participants** | **Variables** | **Data source** | **Data type** |
| Without it, I am not sure I would still be here: a mixed methods service evaluation for online EMDR trauma therapy in a primary care network in England. | 2023 | Frontiers in psychiatry | Kaptan SK and Dernedde C and Dowden T and Akan A | A | M | Turkey and UK | Psychology/ mental health | 139 | No | There are very long waiting lists for psychological therapy in the NHS in England and new service models may be required to address it. | To evaluate the feasibility of a new service delivery model and intervention, and explore effectiveness of EMDR therapy when delivered online in alleviating symptoms | Primary care | Patients with trauma who presented to GP services | Gender, age, referral reason, referring practice, and clinical outcome measures: GAD-7, PHQ-9, PCL-5, W&SAS | Patient records plus questionnaire | Mixed (but qualitative data is not clinical/routinely collected) |
| A 12-Month Clinical Audit Comparing Point-of-Care Lactate Measurements Tested by Paramedics with In-Hospital Serum Lactate Measurements. | 2018 | Prehospital and disaster medicine | Swan KL and Keene T and Avard BJ | M | M | Australia | Paramedical science | 290 | Yes - the authors identify how there is no evidence about the reliability of pLA "under field conditions." and use this to situate their audit. | sLA is a good prognostic tool for a number of diagnoses and outcomes (including sepsis) but is typically done at the hospital. Understanding whether pre-hospital measures (pLA) are associated with hospital measures would be useful for guiding diagnosis and management. | To ascertain whether pLA is a reliable predictor of sLA. | Ambulance services and emergency deparment | Adult patients using ambulance services, who had pLA measured | pLA | Patient records | Quantitative |
| A clinical audit of anatomical side marker use in a paediatric medical imaging department. | 2016 | Journal of medical radiation sciences | Barry K and Kumar S and Linke R and Dawes E | C | M | Australia | Radiography/Radiotherapy | 201 | Yes- the study is investigating adherence to evidence-based practice guidelines for using anatomical side markers in clinical practice. | It is best practice that antomical side markers are pre-applied to radiographic images but digital advancements mean that this can be done post-image, although evidence has suggested that this does not always happen, meaning images are missing the important markers. | This audit was to examine presence of anatomical sidemarkers (traditional and digital markers) in a sample of radiographic images from one hospital, whether they were correct, and wherther there were any differences in this in relation to selected variables. | Hospital | All paediatric patients who had radigraphic examination in a randomly selected 48 hour period. | Patient age (grouped), Presence of marker (lead or digital), incorrect of absence marker, appropriate marking. | Patient records | Quantitative |
| A clinical audit of the management of patients with a tracheostomy in an Australian tertiary hospital intensive care unit: Focus on speech-language pathology. | 2011 | International journal of speech-language pathology | Freeman-Sanderson A and Togher L and Phipps P and Elkins M | M | M | Australia | Speech and language therapy | 140 | Yes - the authors comment on the use of audit and benchmarking for quality improvement of services. | There is little information guiding practice about the timing of SLT input with adult tracheostomy patients and no benchmark/guideline. | To understand more about the profile of patients who receive a tracheostomy and their SLT input, especially the timing of insertion and the timing of recovery of certain functions. | Hospital | Patients coded in the system as 'Trache' patients and/or 'diagnosis related group' codes referring to tracheostomy | gender, age, admitting diagnosis, co-morbidities, intubation history, tracheostomy insertion details, mechanical ventilation hours, weaning variables, day of cuff deflation, speech-language pathology intervention, day of phonation, day of oral intake, decannulation, length of stay in the ICU and in hospital, and final discharge destination. day of cuff deflation, speech-language pathology intervention, day of phonation, day of oral intake, decannulation, length of stay in the ICU and in hospital, and final discharge destination | Patient records | Quantitative |
| A Clinican-friendly test battery with a passing rate similar to a 'gold standard' return-to-sport test battery 1 year after ACL reconstruction: Results from a rehabilitation outcome registry. | 2023 | Physical Therapy in Sport | Broman, Daniel and Piussi, Ramana and ThomeÃ©, Rol and Hamrin Senorski, Eric | C | M | Sweden | Physiotherapy | 588 | Yes- the authors challenge the evidence-based 'gold standard' in practice for assessing muscle strength in ACL reconstruction (isokinetic strength tests), and use the study to test a 'C friendly' alternative. | Gold standard tests for assessing muscle strength after anterior cruciate ligament injury and reconstruction are expensive and are not 'C friendly' and an alternative 'C friendly' battery has been proposed. The 'passing rate' for patients according to cut-off scores for batteries which only contain C-friendly items have not been investigated. | To identify passing rates for the C friendly tests and test batteries, and explore whether there is any difference in passing rates of these batteries, versus the gold-standard, one year post-reconstruction. | ? Rehabilitation services | Adult patients who had ACL injury or reconstruction with 1 year follow up data in the registry. | Muscle function tests, patient-reported outcomes, sex, Tegner activity scale, graft choice, weight, height and body mass index. | Patient records via Registry | Quantitative |
| A comparison of treatment signatures of high and low performing physical therapists for patients with lower back pain: analysis of spine care from a physical therapy outcomes registry. | 2022 | The spine journal : official journal of the North American Spine Society | Lutz AD and Windsor BA and Shanley E and Denninger TR and Harrington SE and Thigpen CA | M | M | USA | Physiotherapy | 1240 | No | There is great variability in patient outcomes, disability severity explains part of this. Part may be due to the treating C. Cs supporting patients that have better therapy outcomes may be doing something different and this should be explored. | To compare 'treatment signature' of physiotherapists who treat patients leading to particularly favourable outcomes ('outperforming') to those with particularly unfavourable outcomes ('underperforming). | ? Rehabilitation services | Patients who completed physiotherapy for non-operative lower back pain and were recorded on the ATI Patient outcomes registry. | Current procedure terminology (CPT) codes - describing intervention approach and corresponding classification for 'treatment signature', patient insurance provider, disability status, sex, age (deciles), patient reported outcome measures. | Patient records via Registry | Quantitative |
| A completed audit cycle of the lateral scan projection radiograph in CT pulmonary angiography (CTPA); the impact on scan length and radiation dose. | 2013 | Clinical radiology | Rodrigues JC and Negus IS and Manghat NE and Hamilton MC | C | M | England | Radiography/Radiotherapy | 50 | Partially- evidence gaps are identified which aim to be fulfilled but does not frame this as an implementation/translation/research to practice issue that a routine data study can address. | Radiation dose should be reduced when possible, though many methods are either not appropriate or have limitations and ideal scan length is debated. Overscanning (and potentially over exposure) are an issue in standard protocols. | To explore whether adding a new imaging component (lateral topogram) to the standard protocol (frontal topogram only) reduces overscanning in CT PA | Hospital | Sample of patients who had undergone CPTA in a 2 month period. | Sex, age, Scan length, estimated effective dose | Patient records | Quantitative |
| A cross-sectional cohort study of speech in five-year-olds with cleft palate +/- lip to support development of national audit standards: benchmarking speech standards in the United Kingdom. | 2014 | The Cleft palate-craniofacial journal : official publication of the American Cleft Palate-Craniofacial Association | Britton L and Albery L and Bowden M and Harding-Bell A and Phippen G and Sell D | C | M | England | Speech and language therapy | 1110 | Yes- the authors aims to establish and examine best practice guidelines which are lacking | Process and outcome standards are required for cleft speech. National data can be used to benchmark practice against these standards. | To develop standards and pilot them using audit data | Regional cleft centres (specialist centres) | Children with cleft accessing specialist centres | Year of birth, CAPS-A outcomes, presence of structual problems and/or surgeries, specialist centre | Patient records | Quantitative |
| A Network Analysis of Clinical Variables in Chronic Pain: A Study from the Swedish Quality Registry for Pain Rehabilitation (SQRP). | 2021 | Pain medicine (Malden, Mass.) | Ã…kerblom S and Cervin M and Perrin S and Rivano Fischer M and Gerdle B and McCracken LM | M | M | Sweden | Mixed- pain rehabilitation | 2241 | Partially- evidence gaps are identified which aim to be fulfilled but does not frame this as an implementation/translation/research to practice issue that a routine data study can address. | Response to chronic pain treatment is variable and there is lots of mediating factors. Network analysis is useful for identifying whether there are network properties that may be related to treatment response. | To determine the 'network structure' of patients and treatment response, to compare pre/post scores in relation to these, and identify if there are any particular properties that might be central to changes arising from treatment. | Specialist pain clinics. | Patients referred into specialist pain clinics | Gender, age, years since pain onsent, working status, educational status, place of bird, referring organisation, measures on a range of clinical indicators (inc depression, anxiety, pain intensity) | Patient records via Registry | Quantitative |
| A retrospective audit exploring the use of relaxation as an intervention in oncology and palliative care. | 2008 | European journal of cancer care | Miller J and Hopkinson C | C | C | England | Occupational therapy | 327 | Yes - the authors identify that most research on relaxation therapy focuses on nursing, there is a gap relating to occupational therapy. | Relaxation interventions are aligned with occupational therapy principles though there is lack of research on the intervention delivered by occupational therapists. | Identify who was referred for relaxation intervention, what approaches to intervention were used, and the effectiveness of it. | Tertiary care centre | Adults with cancer referred to occupational therapy service | Gender, referral source, setting, diagnosis, age, participation in intervention, reason for non-involvement, intervention technique, number of sessions, initial and post-tension score (Visual Analogue Scale) | Patient records | Quantitative |
| A retrospective service audit of a mobile physiotherapy unit on the PGA European Golf Tour. | 2012 | Physical therapy in sport : official journal of the Association of Chartered Physiotherapists in Sports Medicine | Smith MF and Hillman R | A | M | England | Physiotherapy | 7430 | Yes - the authors highlight the limited data on what occurs in practice, and understanding what treatments are delivered would support further research | Professional golf players are at high risk of developing golf-specific injuries at multiple times throughout the reason. Recovery is supported by prompt diagnosis and treatment which can be provided through a mobile physiotherapy unit at events. | To document the service provision of a mobile physiotherapy unit to support better diagnosis and management going forward. | Mobile physiotherapy unit | Professional golfers admitting to the unit | Year of admission, Type of injuiry, location of injury, type of treatment | Patient records | Quantitative |
| A retrospective study of patients presenting with speech and language therapy needs within multidisciplinary Long COVID services: A service evaluation describing and comparing two cohorts across two NHS Trusts. | 2023 | International Journal of Language & Communication Disorders | Chalmers, Sophie and Harrall, Kate and Wong, Sze Yin and Kablan, Widad and Clunie, Gemma | M | M | England | Speech and language therapy | 168 | Yes- the authors identify limited research into SLT needs in Long COVID and MDT services and aims to provide evidence to bridge this gap and enhance practice. | People with long COVID may experience symptoms that can be supported by SLT. More evidence in needed that examines the level of need and how services respond to it to guide practice. | To document the service needs and provision for SLT support for long COVID patients from 2 NHS long covid services. | 2 Long COVID services in NHS trusts | Patients with long covid | Age, gender, referral source, referral reason, diagnosis, intervention provided, MDT involvement | Patient records | Quantitative |
| A service evaluation and improvement project: a three year systematic audit cycle of the physiotherapy treatment for Lateral Epicondylalgia. | 2018 | Physiotherapy | Barratt PA and Selfe J | M | M | England | Physiotherapy | 182 | Partially- evidence gaps are identified which aim to be fulfilled but does not frame this as an implementation/translation/research to practice issue that a routine data study can address. | Outcomes from physiotherapy service for Lateral Epicondylalgia (LE) appeared sub-optimal. An audit cycle was initiated to improve quality. | To audit the service with respect to understanding treatments used and outcomes from them. | Physiotherapy outpient service | Patients with a diagnosis of LE | Number of appointments, treatment completion rates, pre-and post clinical measures using Visual Analogue Scales, Global Rating of Change Scales; Pain Free Grip Strength (PFGS), Patient-Rated Tennis Elbow Evaluation (PRTEE), Tampa Scale for Kiniesiophobia-11 (TSK-11). | Patient records | Quantitative |
| A service evaluation of current practices in the assessment of mental-health and referral for support following disclosure of sexual violence. | 2023 | International journal of STD & AIDS | Clarke J and Hyde A and Caswell RJ | A | M | England | Psychology/ mental health | 179 | Yes- the authors highlight an absence of clinical standards for supporting people who have disclosed sexual violence in Sexual Health and Reproductive Services (SHRS), even though evidence indicates early access to support has positive impacts. | Individuals disclosing sexual violence are best supported by a timely discussion about mental health, and further referrals. No standards exist to guide these practices. | To understand more about the profile of individuals who disclose sexual violence at the service and ascertian whether mental health has beeen assessed, or onward referral made. | Sexual and Reproductive Health Service | Patients presented to SRHS who indicated experiences of sexual violence | age, gender, time frame of sexual violence, documentation of mental health assessment, onward referral rate, services offered | Patient records | Quantitative |
| A Service Evaluation of Prehospital Blood Transfusion by Critical Care Paramedics in British Columbia, Canada. | 2021 | Air medical journal | Greene A and Vu EN and Archer T and Norman S and Trojanowski J and Shih AW | C | M | Canada | Paramedical science | 48 | No | Early blood transfusion for trauma patients supports good outcomes and can be given during transport by emergency services. This practice had been newly introduced in a service. | To evaluate how the introduction of pre-hospital transfusions and the outcomes of those who received it. | Emergency services | Patients who had received prehospital transfusion | Gender, age, injury severity score, assessment of blood consumption, early blood transfusion needs, revised assessment of bleeding and transfusion; type of call, mode of transport, etiology, clinical intervention, haemodynamic parameters, venous blood gas parameters, time of arrival at hospital | Patient records | Quantitative |
| A service evaluation of short-term mentalisation based treatment for personality disorder. | 2021 | BJPsych open | McGowan NM and Syam N and McKenna D and Pearce S and Saunders KEA | C | M | England | Psychology/ mental health | 176 | Yes- the authors highlight tension between research indicating treatment efficacy but lack of service availability in practice and ability to provide long-term treatments. | There is evidence to suggest mentalisation-based treatment is effective for people with personality disorder. There are challenges with waiting lists and acecss to services in the UK. It is not known whether mentalisation-based treatment can be effective if delivered more short-term. | To evaluate a service offering a short-term version of the therapy and examine patient outcomes from this. | Outpatient complex needs service | Individuals referred to the service with a confirmed diagnosis of personality disorder. | Gender, age, response rate to outcome measures, time of recording measures, personality disorder diagnosis, Pre and post Self report instruments (The Mentalization Questionnaire (MZQ);Client Satisfaction Questionnaire (CSQ-8) Emotional Reactivity Scale (ERS); General Health Questionnaire (GHQ-12), Social Functioning Questionnaire (SFQ); McLean Screening Instrument for Borderline Personality Disorder (MSI-BPD)) | Patient records | Quantitative |
| A service evaluation of the assessment process in a Step4 Psychological Therapies Service. | 2023 | Mental Health Review Journal | Robinson, Amie and De Boos, Danielle and Moghaddam, Nima | A | A | England | Psychology/ mental health | 185 | Partially- evidence gaps are identified which aim to be fulfilled but does not frame this as an implementation/translation/research to practice issue that a routine data study can address. | There is a national model for delivery of psychological therapies though access to this is varied and there are challenges with efficiency. There is a need to evaluate the practices happening within services to understand where efficiency may be being impacted. | To examine patient and service outcomes to understand the extent and implications of 'did not attends' and interrogate referral/discharge practices in light of time taken. | Step4 Therapies service. | Adults with personality disorder referred into Step4 care | Clinical Outcomes in Routine Evaluation Outcome Measure-34-item (core-34), University of Rhode Island change assessment (URICA); 'readiness for therapy'; referral letters, service contact, nature/severity of mental health difficulty, risk level. Ad-hoc demographic information including ethnicity, gender, age; Clinical and administrative staff time-taken questionnaires | Patient records | Quantitative - but does use small vignettes to illustrate results |
| A service evaluation of the management of patients with suspected cauda equina syndrome from an outpatient physiotherapy service in the United Kingdom. | 2022 | Musculoskeletal science & practice | Paling C and Hutting N and Devoto K and Galdeano J and Josling K and Goodway L | PS | M | England | Physiotherapy | 231 | Partially- evidence gaps are identified which aim to be fulfilled but does not frame this as an implementation/translation/research to practice issue that a routine data study can address. | Best practice guidance for managing suspected CES has changed substantially in recent years in response to emerging evidence and service management hs changed in response to this. Understanding current management/provision is useful for identifying future improvements. | To evaluate how patients with suspected CES are managed in the physiotherapy outpatient service. | Physiotherapy outpient service | Patients with suspected CES referred to service | Age, gender, symptoms, imaging received, diagnosis, treatment received, other referrals, other contact with professionals | Patient records | Quantitative |
| Access and barriers to supports for children and caregivers attending public child developmental assessment services: Findings from the Sydney child neurodevelopment research registry. | 2023 | Autism research : official journal of the International Society for Autism Research | Boulton KA and Hodge A and Levu K and Ong N and Silove N and Guastella AJ | M | M | Australia | Mixed - neurodevelopmental disorders | 202 | Yes- the authors acknowledge evidence-based recommendations but identify issues with implementing this in practice. The study is situated in the context of understanding the real-world experience. | Difficulties in accessing services are well-established and there are often long waiting lists. Caregivers are sometimes given supports whilst waiting to accessing services though thre are concerns that it will be particularly vulnerable families that are the least likely to access these. | To identify what currently exists to support families waiting to access services, and understand barriers to this (and whether socio-demographic factors were associated with barriers) | Child Development Centre within a hospital | Caregivers of children who attended the centre | Gender, highest level of education, family income, parental relationship status, culturally and linguistically diverse status, gender of child, support services accessed, reported issues with access, reported barriers to access, access to national disability insurance scheme, caregiver support accessed | Patient records | Quantitative |
| Access, timing and frequency of very early stroke rehabilitation - insights from the Baden-Wuerttemberg stroke registry. | 2016 | BMC neurology | Reuter B and Gumbinger C and Sauer T and WiethÃ¶lter H and Bruder I and Diehm C and Ringleb PA and Hacke W and Hennerici MG and Kern R | C | M | Germany | Mixed- stroke rehabilitation | 108577 | Partially - the authors identify highly relevant evidence and question how clinical practice may need to change in response to this, and situates the study within this. | The evidence around very early rehabilitation in stroke care in inconsistent with research indicating positive effects and negative effects including recent findings from a phase III RCT. It is useful to understand how very early rehab looks like in clinical practice. | To provide a detailed insight into the provision of physiotherapy, occupational therapy and speech and language therapy in stroke patients in clinical practice | Hospital | Adults with iscemic stroke or intracerebral haemorrhage who were hospitalised | Gender, age, diagnosis, NIHSS score, presence of paresis, aphasia, dysarthria, level of stroke care accessed, admitting ward, pneumonia status, median length of stay, modified Rankin Scales, number of sessions of PT, OT, SLT and timing of first session | Patient records via Registry | Quantitative |
| Accuracy of clinical diagnosis of internal derangement of the knee by extended scope physiotherapists and orthopaedic doctors: retrospective audit. | 2002 | Physiotherapy | J, Gardiner and P, Turner | C | C | England | Physiotherapy | 128 | No | Evidence suggests extended scope physiotherapists (ESPs) are useful in service development and reduces costs. ESPs were introduced at the local setting and arthoscopy was an area identified that could be supported by ESP, which is a cost-effective procedure. The clinical decision making processes by an ESP or medical professional should be compared. | To identify if an ESP was making good clinical decisions in comparison to medical professionals, to examine diagnostic accuracy, and review standards. | Outpatient physiotherapy service | Patients requiring arthoscopy | Clinical contact of patients, diagnosis, adherance to standards | Patient records | Quantitative |
| Achilles tendon diagnostic ultrasound examination: A locally designed protocol and audit. | 2014 | International Musculoskeletal Medicine | Liffen, Neil | C | N/a | England | Physiotherapy | 30 | Partially - the author discusses relevant evidence in support of the intervention and identifies a gap in the best-practice guidelines (a standard protocol), which is the purpose of the study. | A protocol for Achilles tendon diagnostic ultrasound has not been standardised, despite evidence to suggest its recommendation. A service developed a protocol for testing. | To examine adherance to the protocol and make recommendations for changes. | Physiotherapy department of a hospital | Adults referred to department with diagnosis of mid-portion Achilles tendinopathy | Gender, age, adherance to imaging protocol standard. | Patient records | Quantitative |
| Adequacy of clinical information in X-ray referrals for traumatic ankle injury with reference to the Ottawa Ankle Rules-a retrospective clinical audit. | 2020 | PeerJ | Gomes YE and Chau M and Banwell HA and Davies J and Causby RS | A | M | Australia | Physiotherapy | 262 | Yes- the authors identify evidence and best-practice guidelines but challenge the implementation of these in practice and situate the study in this context. | The Ottawa Ankle Rules (OAR) is an evidence-based clinical decision making tool but adoption and implementation of it is variable across contexts. Little is known about it's implementation in the region. | To assess implementation of OAR in the local context, and examine trends across referring professionals | Tertiary care centre | Adults referred for x-ray presenting with acute ankle injuries in emergency department | Gender, age, duration of injury, reason for injury, referring profession, adherance to OAR criteria | Patient records | Quantitative |
| Advanced practice physiotherapy-led triage in Irish orthopaedic and rheumatology services: national data audit. | 2018 | BMC musculoskeletal disorders | Fennelly O and Blake C and FitzGerald O and Breen R and Ashton J and Brennan A and Caffrey A and Desmeules F and Cunningham C | A | M | Ireland | Physiotherapy | 13981 | Partially- evidence gaps are identified which aim to be fulfilled but does not frame this as an implementation/translation/research to practice issue that a routine data study can address. | Advanced practice physiotherapists can help waiting list management and patient outcomes. Not enough is known about whether this is the case nationally. | Examine patient wait-times, explore decision-making pathways as well as outcomes and re-referral rates. | Tertiary care - triage service | Patients referred to MSK service | MSK disorder, referral source, clinical outcomes, wait time, returning appointments, onward referrals | Patient records | Quantitative |
| An audit of anthropometric measurements by medical and physiotherapy staff in patients with ankylosing spondylitis. | 1998 | Clinical rehabilitation | Lubrano E and Butterworth M and Hesselden A and Wells S and Helliwell P | A | M | England | Physiotherapy | 182 | No | People with Ankylosing spondylitis (AS) are closely monitored by health services with follow up appointments with medics and physiotherapists which may be via different clinics. Arising from this, patient information may be recorded differently, incompletely or in duplicate. | To evaluate the completeness of medical records of people with AS in the varied follow-up clinic settings in line with newly developed standards for record keeping. | Outpatient clinics | People with AS referred in to the participating sites | Age, gender,height, type of AS, duration of disease, anthropometric measures (chest expansion, cervical rotation, tragus or occiput to wall distance, modified Schober’s flexion and extension, side lumbar flexion, intermalleolar abduction, interfingertip abduction), NSAID usage,sulphasalazine usage, eye disease, aortic incompetence, renal disease, attendence to clinic in given period, number of contacts in given period, professional seen at contact. | Patient records | Quantitative |
| An audit of attendance at occupational therapy by long-term psychiatric in-patients at Weskoppies Hospital. | 2011 | South African Journal of Occupational Therapy | KrÃ¼ger, Christa and van der Westhuizen, RonÃ©l | M | M | South Africa | Occupational therapy | 261 | Yes - the authors identify gaps in the research about what OT intervention should be provided to psychiatrics patients, as well as the research using homogenous study populations or one type of therapy, and aims to provide insight to address these gaps, from practice. | Occupational therapy can be useful for psychiatric patients but not much is understood about the type of therapy that is provided. This study provides an audit of OT offered in a large psychiatric hospital. | To understand what the main types of therapy were that were provided, and which approaches were attended most frequently. | Psychiatric hospital | Long-term in-patients with psychiatric disorders at the hospital | Ward (based on functioning), ward type (open or closed), gender, diagnosis, level of functioning, therapeutic interventions offered, timing of intervention, attendance to inteventions | Patient records | Quantitative |
| An audit of botulinum toxin therapy services for adult spasticity...including commentary by Richardson D | 2008 | International Journal of Therapy & Rehabilitation | M, Sakel | C | n/a | England | Mixed- physiotherapy and occupational therapy | 100 | Partially- evidence gaps are identified which aim to be fulfilled but does not frame this as an implementation/translation/research to practice issue that a routine data study can address. | Botulinum toxin is recommended by research and has been newly introduced in practice following best-practice recommendations. | To measure the standard of the new service by auditing it in line with the RCP guidance. | MDT mixed service | Adults with muscle spasticity who were referred to the service | Gender, age, diagnosis, evidence of co-ordinated management, documentation on arrangements for post-injection therapy (physiotherapy and occupational therapy), treatment goals, provision of information, consent, injection solution and brand, dosage, intitial assessment and type, adverse reactions, | Patient records | Quantitative |
| An audit of follow-up chest radiography after coronary artery bypass graft. | 2006 | Clinical radiology | Karthik S and O'Regan DJ | C | C | England | Radiography/Radiotherapy | 666 | No | Chest radiography is routinely performed as a follow up to patients who have had a coronary artery bypass graft. The impact on patient management is unknown. | To explore the impact of chest radiography with regards to its value in identify new abnormalities, the correlation with clinical opinion, the need for further intervention and any financial consequences to the service | Follow up clinical | Patients who had received their first coronary artery bypass graft | Mortality status, attendance at clinic, performance of chest radiography, identification of abnormality via radiography and clinical opinion, adverse reactions/complications | Patient records | Quantitative |
| An Audit Of Obsessive Compulsive Disorder in A Bedford (UK) Community Mental Health Team. | 2014 | Psychiatria Danubina | Yi K and Austin J and Agius M and Zaman R | C | M | England | Psychology/ mental health | 1334 | Partially- evidence gaps are identified which aim to be fulfilled but does not frame this as an implementation/translation/research to practice issue that a routine data study can address. | There are clinical guidelines which state best practice. It is unknown what is happening in the local context with regards to best practice. | To explore the appropriateness of therapy that had been delivered, other treatments provided and the nature of patients receiveing it | Community mental health team | Patients with OCD under the local community mental health team | Diagnosi and comorbid diagnoses, age, care co-ordinator, referral to psychology, psychological therapy status, medication used, medication dose | Patient records | Quantitative |
| An Audit of Occupational Therapy Outpatient Attendance. | 1997 | British Journal of Occupational Therapy | Green, Andrew | C | N/a | England | Occupational therapy | 126 | Partially- evidence gaps are identified which aim to be fulfilled but does not frame this as an implementation/translation/research to practice issue that a routine data study can address. | There are challenges in engaging patients with mental health disorders' attendance to their occupational therapy appointments. New practices were put in place in the lcoal service to address this. | Using audit, to explore the attendance rates in relation to the ways in which the referal to OT had been set up. | Hospital - outpatients | Adults with neuropsychiatric disorders who had been referred to occupational therapy. | Gender, age, referall source, doctor and therapist, diagnosis, attendance at assessment, attendance at therapy, completion rate, agreed discharge reason. | Patient records | Quantitative |
| An audit of patient records into the nature of pulsed shortwave therapy use...including commentary by Dziedzic K, and Callaghan MJ | 2006 | International Journal of Therapy & Rehabilitation | MM, Al-M and eel and T, Watson | A | A | England | Physiotherapy | 1750 | Yes - the authors identify a gap in research on the clinical application of pulsed shortwave therapy, which the current study aims to fulfill. | Physiotherapists are expected to maintain good clinical documentation including outcome measures. More evidence is needed about the clinical application of PSWT which despite limited information, is a very popular appraoch to therapy. | To explore the use of PSWT in outpatient clinics and evaluate the clinical documentation. | Physiotherapy outpatient service | Patients referred to physiotherapy departments | Departmental counts of patients being seen and treated with PSWT, plus individual patient data on the number of PSWT sessions per week, number of sessions until discharge, treatment dose, outcome measures, diagnosis. | Patient records | Quantitative |
| An audit of physiotherapists' documentation on physical activity assessment, promotion and prescription to older adults attending out-patient rehabilitation. | 2022 | Disability and rehabilitation | Paim T and Low-Choy N and Dorsch S and Kuys S | M | M | Australia | Physiotherapy | 56 | Yes- the authors identify relevant evidence and best practice for physical activity intervention but state that little is known about whether physiotherapists do this for older adult rehabilitation in practice. | Physical activity interventions for older adults may help to maintain gains as part of a healthy lifestyle from rehabilitation, which is supported by evidence. Outpatient physios are well-placed to offer this. It is not known how much physiotherapists actively incorporate transitional physical activity intervention in to rehabilitation. | Evaluate the extent to which physios assesed, promoted and prescribed physical activity intervention to older adults as part of their rehabilitation plan. | Out-patient rehabilitation srvice | Patients aged 65 years or older who attended initial physiotherapy assessment at the rehabilitation unit. | Age, gender, living situation, referral reason, mobility level, gait aid used, type of intervention, whether physical activity was assessed, whether physical activity was promoted, whether physical activity was prescribed, whether information on physical activity to transition to the community was given | Patient records | Quantitative |
| An Audit of Referrals to Occupational Therapy for Older Adults attending an Accident and Emergency Department. | 2004 | British Journal of Occupational Therapy | Smith, Tony and Rees, Val | C | M | Wales | Occupational therapy | 1036 | No | Occupational therapists are well-placed to evaluate the functional status of patients admitted to emergency services. There is evidence to suggest that older people are poorer at judging their abilities and needs, and research that indicates having occupational therapists in A&E departments to understand these needs can have benefits. | To evaluate the effectiveness of having occupational therapists in A&E departments as part of a new service design. | Accident and emergency departments | A&E patients over the age of 45 who were referred to occupational therapy for assessment | Gender, age, diagnosis, time of referal, discharge decision, occupational therapy intervention, onward referral and team liaison, equipment provided, social support provided, readmission. | Patient records | Quantitative |
| An audit of rejected repeated x-ray films as a quality assurance element in a radiology department. | 2008 | Nigerian journal of clinical practice | Eze KC and Omodia N and Okegbunam B and Adewonyi T and Nzotta CC | C | M | Nigeria | Radiography/Radiotherapy | 15095 | No | Radiographic exposure carries a risk, and errors in the multi-disciplinary process of creating an image can lead to the need for repeated radiographic exposure. Quality assurance processes should be in place to reduce the need for additional exposure. One way of measure quality is by examinating the need for 'film rejection' leading to repeat scans and the reasons they were required. | To identify a baseline to monitor improvement on with regards to film rejection / repeat, and the reasons underlying them. | Hospital radiology department | Radiographic films from patients who were referred to the hospital for x-ray. | Repeat/rejection status, fault reason, film size, | Patient records | Quantitative |
| An audit of the management of depression in a community population with intellectual disabilities in accordance with NICE guidelines. | 2011 | British Journal of Developmental Disabilities | da Costa, Edward and Koyee, Promod and Bogdan, Nadine and Qassem, Tarik | C | M | England | Psychology/ mental health | 73 | Partially- evidence gaps are identified which aim to be fulfilled but does not frame this as an implementation/translation/research to practice issue that a routine data study can address. | Best-practice guidance have recently been created for the management of depression which can be applied to individuals with intelletual disability. | To audit the management approaches implemented in community services for people with intellectual disability | Specialist intellectual disabilities community service | Patients with intellectual disability receiving care from the service | Gender, marital status, employment status, living situation, severity of intellectual disability, depression severity and state, adherance to standards | Patient records | Quantitative |
| An audit of velopharyngeal incompetence treated by the Orticochea pharyngoplasty. | 1996 | British journal of plastic surgery | James NK and Twist M and Turner MM and Milward TM | C | C | England | Speech and language therapy | 56 | No | Research shows that pharyngoplasties have a positive effect on speech skills of people with velopharyngeal incompetence. Variations of the procedure have been attempted in research, to improve the effects. | To comparethe outcomes from use of an unmodified procedure in clinical practice with the outcomes identified in the research on modified techniques. | Plastic surgery | Patients with velopharyngeal incompetence referred for surgery. | Age, gender, aetiology of nasal escape, degree of nasal escape, degree of resonance, complications from surgery | Patient records | Quantitative |
| Appropriateness of end-of-life care for children with genetic and congenital conditions: a cohort study using routinely collected linked data. | 2023 | European journal of pediatrics | Piette V and Deliens L and Debulpaep S and Cohen J and Beernaert K | A | M | Belgium | Mixed- end of life care | 1934 | Partially- evidence gaps are identified which aim to be fulfilled but does not frame this as an implementation/translation/research to practice issue that a routine data study can address. | There is little research into end of life treatment options for children with congenical/genetic complex conditions, though previous research suggests end of life care for children is poor. These account for a large proportion of paediatric deaths. Quality indicators for care have previously been developed. | To eevaluate appropriateness of end of life care for children using quality indicators. | National cohort study | Children with congenital/genetic complex conditions who had died in Belgium | Diagnosis, gender, age, nationality, household make up, 'comfort' of household, urbanicty, household income, cause of death, presence/absence of quality indicator in care | Patient records via linked databases and registries. | Quantitative |
| Are there missed opportunities for occupational therapy for people with dementia? An audit of practice in Australia. | 2018 | Australian occupational therapy journal | Rahja M and Comans T and Clemson L and Crotty M and Laver K | A | M | Australia | Occupational therapy | 87 | Yes - the authors explictly identify a research to practice gap between clinical guidance and what occurs, and situate the study as one way to address this. | Research suggests that occupational therapy intervention can delay functional decline in people with dementia, and increase dependence, quality of life and wellbeing. Implementation of these interventions in practice is not widely researched, and therapists spend most their contact with this patient group on assessment rather than intervention. Little evidence exists about what therapy is actually delivered in practice. | To identify the common assessment and therapy approaches for people with dementia that occupational therapists implement in practice | Community geriatric services | People with dementia recieving care from the participating services who had been referred to occupational therapy. | Age, gender, living arrangement, cognitive assessment scores, clinical assessment scales, duration of intervention, number of visits per referral, type of contact, assessments conducted, interventions used, | Patient records | Quantitative |
| Assessment of a reporting radiographer-led discharge system for minor injuries: a prospective audit over 2 years. | 2013 | Emergency medicine journal : EMJ | Henderson D and Gray WK and Booth L | C | M | England | Radiography/Radiotherapy | 497 | No | Emergency departments face large demand and waiting times are often long, in conflict with the ambitions of the NHS. Radiographers may have the required expertise to offer a discharge scheme in emergency departments and reduce waiting times. | A new radiography-led discharge service was set up in an emergency department. The aim was to evaluate whether it had an impact on waiting times. | Accident and emergency departments | People who had been admitted to A&E with an injury either below the elbow or below the knee. | Gender, age, type/location of injury, diagnostic accuracy, waiting time at each step of care pathway, re-admittance, use of 'see and treat' | Patient records | Quantitative |
| Association of Musculoskeletal and Radiological Features with Clinical and Serological Findings in Systemic Sclerosis: A Single-Centre Registry Study. | 2020 | Mediterranean journal of rheumatology | Azarbani N and Javadzadeh A and Mohseni I and Jalali A and Andalib E and Poormoghim H | A | A | Iran | Mixed- Radiography/Radiotherapy and Biomedical sciences | 180 | No | There is some evidence suggesting MSK involvement is common in systemic sclerosis, and hand radiography may indicate these signs. It it useful to have more than one parameter to evaluate this. | To examine how common MSK manifestation was in patients, and to evaluate the correlation across disease parameters. | Hospital | People with Systemic sclerosis who had attended the hospital and had radiography of their hand. | Age, gender, skin score, disease duration, presence of clinical markers/indicators (angiectasia, amputation, calcinosis and MSK involvement), serological scores | Patient records | Quantitative |
| Audit and re-audit of the CSP Core Standards of Physiotherapy Practice. | 2000 | Physiotherapy | M, Sumner and J, Mead and ten Hove R | PS | PS | UK | Physiotherapy | 650 | No | New standards were recently published regarding physiotherapy pracitce, along with clinical audit tools. | To audit whether physiotherapy services were meeting the new standards, utilising the new audit tools. | Mixed - 8x different types of sites providing a range of physiotherapy services | Patients referred to physiotherapy departments | Yes/No conformance to standards demonstrated in patient records | Patient records | Quantitative |
| Audit from preschool developmental surveillance of vision, hearing, and language referrals. | 1991 | Archives of disease in childhood | Rona RJ and Reynolds A and Allsop M and Morris RW and Morgan M and Mandalia S | C | C | England | Speech and language therapy | 664 | No | Concerns had been raised about the effectiveness of routine assessments evaluating youg children's health in the areas of vision, hearing and language though it had never been formally evaluated. | To audit routine practice in these clinical areas within one region and examine the steps taken when a difficulty was identified. | Early years services | Children referred to either audiology, speech and language therapy, or opthalmology under the age of 4.5 years | Gender, age, service referred to, attendance at appointments, reasons for non-attendance, outcome (discharge/in system), diagnosis/difficulty if identified, specific clinical markers (e.g. articulation score, visual acuity defect, etc). | Patient records | Quantitative |
| Audit of completion of radiology request form in a nigerian specialist hospital. | 2012 | Annals of Ibadan postgraduate medicine | Afolabi OA and Fadare JO and Essien EM | C | C | Nigeria | Radiography/Radiotherapy | 202 | No | Radiology request forms are crucial components of communication between Cs and radiographer, and should be kept to a high standard. Previous research shows a substantial proportion of radiographic examination requests are inappropriate or unhelpful. High quality request forms may improve this situation. | To evaluate the quality and completeness of radiography request forms utilised in the site's radiology department | Radiology department | People who were referred for x-ray at the hospital in the given timeframe | Name, Age, gender, unit and ward information, address, body part to be examined, provision of clinical information, clinical assessment information, information about previous surgeries, information about previous x-rays, previous films and x-ray numbers. | Patient records | Quantitative |
| Audit of fractured neck of femur integrated care pathway. | 2005 | Journal of Integrated Care Pathways | M, Hempling and A, Adhikari | C | C | England | Physiotherapy | 55 | No | A new pathway was introduced for patients with fractured neck of the femur | To examine the quality of medical documentation in the new pathway and identify areas to improve communication across the MDT | Accident and emergency departments | Patients presenting with fracted neck of femur within a given period | Use of pathway, completion of documentation, professionals' use of ICP, filing status, duration of stay on pathway and discontinuation reason. | Patient records | Quantitative |
| Audit of the change in the on-call practices in neuroradiology and factors affecting it. | 2006 | BMC medical imaging | Mukerji N and Wallace D and Mitra D | C | C | England | Radiography/Radiotherapy | 74 | No | Changes had been made in practice meaning that out of hours referrals to neuroradiographs went directly to neuroradiographers rather than via an on-call neuroradiologist, where decisions for appropriateness of scans were guided by a protocol. | To evaluate whether this change impacted workload and to better understand the out-of-hours working patterns. | Radiology department | Cases of people who had been referred to for a neuro-radiograph during out of hours | Number of our of hours referrals, presence of booklet, completion of booklet, examinations conducted and requests for scans made | Patient records | Quantitative |
| Both positive and negative beliefs are important in patients with spine pain: findings from the Occupational and Industrial Orthopaedic Center registry. | 2018 | The spine journal : official journal of the North American Spine Society | Wertli MM and Held U and Lis A and Campello M and Weiser S | M | M | Switzerland | Physiotherapy | 2182 | Yes- the authors acknowledge best practice guidelines in identifying patients at risk of a delayed recovery, and relevant evidence to support the role of beliefs. | Negative and positive beliefs about pain may have an effect on recovery and the impact of treatment of patients with spine pain. Checklists have been developed to screen for beliefs but not much is known about how the results from these interact with treatment outcome. | Evaluate the influence of beliefs on initial presentation and treatment response, including assessing the predictive effect of beliefs on treatment outcome. | Outpatient physical therapy clinic | Patients referred to the service with spine pain | age, gender, educational level, martial status, self-report questionnaires, pain complains, pain intensity, duration, location, perceived disability (Oswestry Disability Index or Neck Disability Index), employment, and STarT Back Screening Tool (distress scales) | Patient records | Quantitative |
| British Thoracic Society Paediatric Pneumonia Audit: a review of 3 years of data. | 2013 | Thorax | Bowen SJ and Thomson AH | C | C | England | Mixed- respiratory disease | 891 | No | Best practice guidelines were recently updated. | To undertake an audit to examine adherance to guidelines | National audit | Children with community-acquired pneumonia | Age, gender, admitting institution, clinical indicators (oxygen saturation, costal recession, grunting, wheeze, fever), investigations carried out (chest xray, blood cultures), causative organism if identified, management (medicines and administration mode, physiotherapy given) | Patient records | Quantitative |
| Cancers of the tongue and floor of mouth: five-year file audit within the acute phase. | 2014 | American journal of speech-language pathology | Blyth KM and McCabe P and Heard R and Clark J and Madill C and Ballard KJ | M | M | Australia | Speech and language therapy | 85 | Yes- the authors highlight a gap in the evidence base and indicate how knowledge gained through this study will help bridge the gap. | Research indicates various aspects and elements of speech and language therapy can improve people's outcomes following surgery for head and neck cancer, but specific components of intervention and the outcomes from this are often poorly reported, especially at the early stages post-therapy. | To evaluate functional speech and language theray outcomes for patients following surgery in the acute phases, including an examination of patient characteristics and rehabilitation factors. | Tertiary hospital | Patients who had undergone specified oral surgery with a relevant cancer diagnosis referred into speech and language therapy | Age, gender, language spoken, alcohol and smoking history, tumour type, surgery type, radiotherapy provided, "postoperative events" (referral to services and further assessments), speech and swallowing measures, intervention information. | Patient records | Quantitative |
| Cardiac Rehabilitation in Abu Dhabi: A Retrospective Investigation of Program Delivery, Participants, and Factors Associated with Program Completion Utilizing a Hospital Registry. | 2023 | Journal of the Saudi Heart Association | Thrush AH | C | N/a | United Arab Emirates | Physiotherapy | 1774 | Yes - the authors refer to evidence to support the use of Cardiac Rehabilitation as well as research on how much it is utilised in the empirical context, highlighting a research to practice gap. | There are high rates of cardiovascular disease in the empirical context. There is good evidence that cardiac rehabilitation supports good outcomes, but programmes are rare in the empirical context and research in the areas is limited. More needs to be known about the current state to advance patient access to these programmes. | Examine use of cardiac rehabilition in Abu Dhabi including how the programme is delivered, who utilises the services and what influences completion | Outpatient physiotherapy service | Patients who have been referred into the service for cardiac rehabilitation | Gender, nationality, residency, insurance status, referral reason (diagnosis), presence of depression, walk test outcomes, age, BMI, EQ5-D and PHQ-9 data, number of sessions completed, evaluation undertaken, follow-up delivered, type of surgery (if relevant) | Patient records | Quantitative |
| Changes in physical activity and the association between pain and physical activity - a longitudinal analysis of 17,454 patients with knee or hip osteoarthritis from the GLA:DÂ® registry. | 2023 | Osteoarthritis and cartilage | Baumbach L and GrÃ¸nne DT and MÃ¸ller NC and Skou ST and Roos EM | A | M | Germany and Denmark | Physiotherapy | 17454 | Yes - the authors comment on recommended practice, issues in literature and that the effect of intervention is unknown "in real world clinical practice". | Research indicates that physical activity is useful to improve pain in people with osteoarthritis, though increasing activity is difficult and the role of joint pain in this unclear. | To evaluate the impact of a physical activity programme on activity levels and to explore associations between change in activity and pain. | Primary care | People with knee or hip osteoarthitis referred to service and in receipt of intervention | Age, gender, educational level, comorbidities, clinical outcomes (UCLA activity scale), pain intensity (visual analogue scales), self-efficacy (ASES scale), medications, quality of life, compliance with programme. | Patient records via Registry | Quantitative |
| Changing occupational therapy and physiotherapy practice through guidelines and audit in the United Kingdom. | 2005 | Clinical rehabilitation | Hammond R and Lennon S and Walker MF and Hoffman A and Irwin P and Lowe D | PS | M | England | Mixed- physiotherapy and occupational therapy | 8200 | Yes - the authors introduce the clinical guidelines and the role of the audit undertaken in evaluating how actual practice aligns with evidence-based standards. | Clinical guidelines were developed to guide practice in physiotherapy and occupational therapy stroke teams. There is a national audit (SSNAP) to collect data on adherance to these guidelines. | To examine physiotherapy and occupational therapy performance against specific clinical recommendations to highlight areas for development. | Hospital - acute care | People admitted to hospital with a stroke in the given timeframe | Time spent on stroke unit, mean length of stay, treating site information (stroke unit, presence of community team, staffing structures, interdisciplinary pathway, in-house training), assessment given in specified timeframe, functional ability measures, documented goal-setting, communication with carers/families, transfer/discharge rates, compliance with rehabilitation. | Patient records | Quantitative |
| Characteristics, outcome, and care of stroke associated with atrial fibrillation in Europe: data from a multicenter multinational hospital-based registry (The European Community Stroke Project). | 2001 | Stroke | Lamassa M and Di Carlo A and Pracucci G and Basile AM and Trefoloni G and Vanni P and Spolveri S and Baruffi MC and Landini G and Ghetti A and Wolfe CD and Inzitari D | A | M | Italy | Mixed- stroke rehabilitation | 4462 | Yes- the authors cite evidence from clinical practice, and a gap in the evidence from traditional research, to situate the study. | Atrial fibrilliation is a major risk factor for stroke but evidence about whether it is an independent determinant of outcomes following stroke is mixed. | To evaluate outcomes following stroke from people with and without atrial fibrillation. | Hospital - acute care | Patients admitted to hospital with stroke | Age, gender, living conditions, drug usage, modified rankin scale (pre-stroke), presence of vascular risk factors or comorbidities (e.g. hypertension, atrial fibrillation), clinical condition in acute stage, usage of tests, discharge destination, type of stroke. | Patient records via Registry | Quantitative |
| Cognitive assessments among children with cerebral palsy in Sweden and the use of augmentative and alternative communication and interpreters: a cross-sectional registry study. | 2023 | Disability & Rehabilitation | O'Regan, Elisabeth and Stadskleiv, Kristine and Czuba, Tomasz and Alriksson-Schmidt, Ann I. | A | M | Sweden | Mixed - speech and language therapy and Psychology / Mental health | 2627 | Yes - the authors outline a gap in research to guide practice, in that there it little information about how to assess children with CP and communication difficulties. | Children with cerebral palsy (CP) show a wide-ranging cognitive profile and this should be routinely assessed in the country (CPCog protocol). A substantial proportion of children with CP also have communication difficulties. There is not much guidance on how to assess cognitive skills of children with CP and communication difficulties. Using AAC and medical professionals to support with interpretation of this may facilitate this process. | To explore adherance to CPCog protocol and associations of cognitive level with a range of factors related to the child and their region, and to explore the use of AAC in these assessments. A second part of the study explored practitioners' perceptions of using AAC. | Rehabilitation centre | Children with cerebral palsy accessing rehabilitation centres submitting data to the registry | age, gender, type of CP, functional status (Gross Motor Function Classification System and Manual Ability Classification System), birthplace, type of cognitive assessment, use of AAC/medical interpreter in assessment. | Patient records via Registry | Quantitative |
| Comparing fly-in fly-out and telehealth models for delivering advanced-practice physiotherapy services in regional Queensland: An audit of outcomes and costs. | 2021 | Journal of telemedicine and telecare | Cottrell M and Judd P and Comans T and Easton P and Chang AT | C | M | Australia | Physiotherapy | 44 | Yes- the authors acknowledge the evidence base for the approach but explictly refer to challenges with implementing it in practice | Evidence supports a model of care where patients with non-urgent musculoskeletal conditions are seen and managed by a physiotherapist, indicating effectiveness and cost-effectiveness. This has been difficult to implement in rural healthcare settings where staff turnover is high. A new model was introduced where a leader from a metropolitan area was flown in to the area for a 2 day clinic. There is also evidence indicating that assessment could be done via telehealth, and this model was also implemented. | To compare the fly-in/fly-out model with the teleheaklh model of service delivery with respect to their economic value and efficiency. | Outpatients physiotherapy | Patients referred into the service in the given time period, with musculoskeletal conditions. | Age, gender, referal source, triage category, body region of concern, appointment attendance/reschedule/failure to attend, clinical actions (maintain in team, refer elsewhere, discharge to urgent care, discharge from service), safety incidents. (plus economic information not routinely collected) | Patient records | Quantitative |
| Criteria audit as a means of assessing the physiotherapy component of stroke rehabilitation. | 1982 | The Australian journal of physiotherapy | Truscott B and Worsam B | C | C | Australia | Physiotherapy | 39 | No | Documentation of physiotherapy records was an existing problem in the department. | To audit the documentation of records with respect to it's quality and also explore the extent and quality of physiotherapy and care provided based on the records. | Physiotherapy department of a hospital | Patients with stroke on the rehabilitation wards | Documentation metrics (assessment, symptoms, discharge summary), outcome metrics (assessment received, gait, steps, arm functio, relatives support, post-discharge treatment), complications, extent of integrated care | Patient records | Quantitative |
| Current physiotherapy practice in the management of tennis elbow: A service evaluation. | 2018 | Musculoskeletal care | Bateman M and Whitby E and Kacha S and Salt E | C | C | England | Physiotherapy | 65 | Yes- the authors outline evidence supporting intervention and treatment pathways but a lack of knowledge about whether this is used in practice. | There is evidence to guide practice on physiotherapy for tennis elbow, but lots of different therapies exist. Exercise-based therapy has a good degree of evidence for effectiveness, but optimum dosage/intensity is not known. It is not known what the specifics of the interventions are that are used in the UK. | To ascertain what physiotherapy interventions are used in the UK and explore the dosage and types of exercises given. | Physiotherapy department of a hospital | Patients referred to physiotherapy by a GP with tennis elbow | age, gender, duration of symptoms, duration of physio, number of sessions, outcome, therapist expertise level, treatments given, type/dosage, exercise progression | Patient records | Quantitative |
| Decreasing the number of arthroscopies in knee osteoarthritis - a service evaluation of a de-implementation strategy. | 2020 | BMC musculoskeletal disorders | Barlow T and Rhodes-Jones T and Ballinger S and Metcalfe A and Wright D and Thompson P | M | M | England | Physiotherapy | 377 | Yes - the authors identify a gap between research and practice guidelines and what is happening in practice. | Recent evidence overturned long-lasting recommendations to perform arthroscopy for knee osteoarthritis and is no longer recommended. Though, research suggests it is still performed, showing an issue with the implementation of new guidance. | To explore the effects of a new programme implemented to address the ongoing issues with adhering to recent practice guidelines, and to see if this reduced the number of arthroscopies. | Orthopaedic clinic (secondary care) | Patients referred to clinic with knee osteoarthritis, who according to traditional criteria would be put forward for knee arthroscopy. | Age, gender, BMI, Oxford Knee Score, EQ-5, number of physiotherapy and dietician sessions, arthroscopy performed | Patient records | Quantitative |
| Determining the sensitivity of emergency dispatcher and paramedic diagnosis of stroke: statewide registry linkage study. | 2022 | Journal of the American College of Emergency Physicians open | Eliakundu AL and Cadilhac DA and Kim J and Kilkenny MF and Bagot KL and Andrew E and Cox S and Bladin CF and Stephenson M and Pesavento L and Sanders L and Clissold B and Ma H and Smith K | A | M | Australia | Paramedical science | 4255 | Partially - the authors cite evidence to support identification of stroke in pre-hospital setting and comment that it is unknown how patient, clinical, and system factors influence staff's accuracy of this. However, they do not mention evidence-to-practice or implementation gaps explicitly. | Early identification of stroke improves outcomes from stroke. Paramedics/dispatchers can facilitate rapid identification, but it is not known what factors may influence this identification. | To evaluate the sensitivity of staff's stroke identification and explore factors associated with this; to explore the extent of complications related to time of arrival in hospital. | Emergency services | Patients with stroke or TIA admitted to participating hospitals |  | Patient records via registry | Quantitative |
| DIAGNOSTIC IMAGING AND IONIZING RADIATION EXPOSURE IN A LEVEL 1 TRAUMA CENTRE POPULATION MET WITH TRAUMA TEAM ACTIVATION: A ONE-YEAR PATIENT RECORD AUDIT. | 2020 | Radiation protection dosimetry | BÃ¥genholm A and LÃ¸vhaugen P and Sundset R and Ingebrigtsen T | M | M | Norway | Radiography/Radiotherapy | 144 | Partially - the authors acknowledge a lack of practice guidance and conflicting evidence, as well as a gap in evidence, but do not explicitly link these. | Radiation exposure is a risk to humans and the use of x-ray and CT is only acceptable if the health gain is greater than harm. Trauma patients may have whole-body CT scans, but the evidence is conflicting about whether this is required and/or effective for good outcomes. Radiation dose is an important factor in this, but there is no evidence about the total radiation dose that a trauma patient may receive across their entire clinical pathway (from accident to rehabilitation). | To record the extent of diagnostic imaging and radiation dose delivered to trauma patients across the pathway. | Hospital - level 1 trauma centre | Patients admitted to trauma centre in given timeframe. | Age, gender, tourist (y/n), mode of transport to hospital, type of trauma, transfer from other hospital, injury severity score, length of stay, mortality at 30 days, x-ray examinations (number and place), CT examinations (number and place), non-ionising examinations (Ultrasound and MRI - number and place), ionizing radiation exposure (dose-area-product, DAP, and dose length product, DPL) | Patient records | Quantitative |
| Do RATs save lives? A service evaluation of an out-of-hospital cardiac arrest team in an English ambulance service. | 2019 | British paramedic journal | Pilbery R and Teare MD and Lawton D | C | C | England | Paramedical science | 5868 | No | The survival rate of people who have a cardiac arrest out of hospital (but who were resuscitated and found to have a shockable rhythm on arrival of ambulance service) is low in the UK. A new intervention was introduced, which included a series of initiatives, to improve rates. Data indicates survival rates have increased but the effect of each initiative is unknown. | To evaluate the impact of one of the initiatives, Red Arrest Teams (RATs) on patient outcomes. | Ambulance services | People with out of hospital cardiac arrest of medical origin and resuscitated, in the given timeframe | age, gender, bystander CPR given, response time by ambulance, RAT response time (if in this arm), witness status, presenting cardiac rhythm, location, resuscitation time, survival measure (return of spontaneous circulation at hospital, survival status (survived, unknown, died) | Patient records | Quantitative |
| Does face-to-face pre-operative joint replacement education reduce hospital costs in a regional Australian hospital? A descriptive retrospective clinical audit. | 2020 | European journal of orthopaedic surgery & traumatology : orthopedie traumatologie | Lewis D and Fullard K and Kolbe T and Chapman S and Divanoglou A and Doran C and Hutton V and Santamaria J and Heales LJ | C | M | Australia | Mixed- physiotherapy and occupational therapy | 326 | No | Osteoporosis is a resource-heavy condition in that many patients require surgery which has associated costs for length of stay in hospital. Pre-surgery education can help reduce length of stay and may reduce healthcare costs. | To evaluate whether patients who engage in pre-surgery education have shorter length of stay in hospital compared to those who did not attend the education sessions. | Hospital | Patients who had total hip or knee replacement in the given timeframe at the hospital. | Age, gender, BMI, surgery received (hip or knee), attendance at education session (attended, did not attend), length of stay, Functional comorbidities index (FCI), living distance from hospital, discharged home, post-op complications, duration between education and surgery, associated costs (length of stay, travel, education) | Patient records | Quantitative |
| Does service timing matter for psychological outcomes in cardiac rehabilitation? Insights from the National Audit of Cardiac Rehabilitation | 2018 | European journal of preventive cardiology | Sumner J and Bohnke JR and Doherty P | A | A | England and Singapore | Psychology/ mental health | 39588 | No | Cardiac rehabilitation should be multi-modal and include management of mental health conditions. Research has indicated that the wait-time for cardiac rehabilitation is associated with fitness-related outcomes. Mental health outcomes may also potentially be impacted by wait-time. | To evaluate the extent of mental health symptoms in patients receiving cardiac rehabilitation, and to explore whether the outcomes related to these are associated with wait-times. | Outpatients’ rehabilitation | People with acute coronary syndrome who completed cardiac rehabilitation from the specific sites. | Age, gender, ethnicity, comorbidities, employment status, marital status, previous cardiac event, previous revascularisation, wait time to begin rehabilitation, rehabilitation programme duration, anxiety symptoms, anxiety score, depression symptoms, depression score, | Patient records | Quantitative |
| Does the presence of an emergency physician influence pre-hospital time, pre-hospital interventions and the mortality of severely injured patients? A matched-pair analysis based on the trauma registry of the German Trauma Society (TraumaRegister DGU(Â®)). | 2017 | Injury | Bieler D and Franke A and Lefering R and Hentsch S and Willms A and Kulla M and Kollig E | C | M | Germany | Paramedical science | 2470 | Partially - the authors acknowledge the conflicting research about which pre-hospital approach is best, the lack of consensus/clinical guidance, and how their study provides a unique insight for a comparison, but do not directly reference evidence to practice gaps or implementation. | There is conflicting evidence about which of the 'stay and play' or 'scoop and run' approaches to pre-hospital care for trauma patients leads to better outcomes, because there is so much heterogeneity in patients and in circumstances (such as region and structural differences). | To compare the outcomes of severely injury patients who received pre-hospital care by either emergency physicians (stay and play) or non-physician teams (scoop and run). | Emergency services | Patients living in Germany who experienced a trauma and were taken to hospital by either non-physician teams or emergency physician teams | age, injury severity score, single severe injuries, injuries, cause of trauma, mechanism of injury, treating pre-hospital team, level of consciousness, presence of shock prior to hospital arrival, outcome at 24 hours, in-hospital mortality and transfusion, pre-hospital time, on-scene time, transport destination, pre-hospital interventions given, vital signs. | Patient records via Registry | Quantitative |
| Dose audit for patients undergoing two common radiography examinations with digital radiology systems. | 2014 | Diagnostic and interventional radiology (Ankara, Turkey) | Inal T and Atac G | A | A | Turkey | Radiography/Radiotherapy | 40 | Yes - the authors refer to best practice guidance on radiation dose but comment that guidance does not yet give diagnostic reference levels for new x-ray technologies (which lower the patient dose) widely used in Turkey and situate their study in this context. | Authorities have not provided a diagnostic reference level for new digital Xray technologies but there is good information about this for conventional approaches - however, digital approaches are often used in Turkey. | To audit radiation dose and compare to international standards and results, and to evaluate the patient dosage from conventional radiography and digital radiography. | Radiology departments in four hospitals in the city. | A sample of patients undergoing chest xrays or kidney-ureter-bladder radiographs. | Patient weight, height, BMI, age, gender, radiography equipment used, type (conventional or digital), entrance surface dosage, exposure factors, focal-skin distance | Patient records | Quantitative |
| Drug-free tracheal intubation by specialist paramedics (critical care) in a United Kingdom ambulance service: a service evaluation. | 2021 | BMC emergency medicine | Houghton Budd S and Alexander-Elborough E and Brandon R and Fudge C and Hardy S and Hopkins L and Paul B and Philips S and Thatcher S and Winsor P | C | C | England | Paramedical science | 663 | Yes - the authors cite relevant evidence of clinical trials outlining the advantage of pre-hospital intubation but comment that they do not differentiate between specialist and non-specialist paramedics, which they intend to do in their study. | Development of critical care specialist paramedics provides the possibility of increasing success of tracheal intubation but only one study in one area has examined this. | To evaluate the tracheal intubation success rate of specialist paramedics in the region. | Emergency services | Patients receiving drug-free tracheal intubation by the specialist paramedics in the given period. | age, gender, weight, Successful attempt of tracheal intubation (first, second or third), grade of laryngoscopy view, use of bougie to facilitate insertion | Patient records | Quantitative |
| Effect of fluid administration on scene to traffic accident patients by EMS personnel: a propensity score-matched study using population-based ambulance records and nationwide trauma registry in Japan. | 2022 | European journal of trauma and emergency surgery : official publication of the European Trauma Society | Katayama Y and Kitamura T and Kiyohara K and Ishida K and Hirose T and Nakao S and Tachino J and Matsuyama T and Kiguchi T and Umemura Y and Noda T and Nakagawa Y and Shimazu T | A | M | Japan | Paramedical science | 10,908 | Partially - the authors refer to best practice guidance and comment that it is unknown whether implementing the intervention as suggested actually improves outcomes. | Guidelines were recently updated and recommended that emergency services staff could secure intravenous lines and administration a specific solution to trauma patients with shock or crush syndrome. Though, the evidence regarding effectiveness is mixed. | To evaluate the impact of fluid administration in this way on patient prognosis. | Emergency services | Patients with significant trauma from traffic accidents who were treated by ambulance services, across Japan in the given timeframe. | Age, gender, timing of accident (weekday or weekend, day time or night time), systolic blood pressure on scene, presence of shock, type of patient (position in car if in vehicle, pedestrian, cyclist, etc), Injury severity score, specific injury site, time to arrive on scene | Patient records via Registry | Quantitative |
| Effects of an electronic health record-based mobility assessment and automated referral for inpatient physical therapy on patient outcomes: A quasi-experimental study. | 2023 | Health services research | Chou A and Johnson JK and Jones DB and Euloth T and Matcho BA and Bilderback A and Freburger JK | C | M | USA | Physiotherapy | 6608 | Partially - the authors comment that research advises rehabilitation from a physiotherapist for to enhance mobility in hospitalised older adults is effective, but also that in practice, staffing is a challenge to promoting mobility. However, they do not indicate that the study may shed light on this. | Physiotherapy for older adults admitted to hospital to promote mobility is beneficial, but patients need to be evaluated on their need for this in order for insurance to cover the expense. Screening patients to identify those who would most benefit from physiotherapy in this way would be useful. | To trial a new screening system embedded in electronic patient records and use the data to evaluate the effectiveness of the screening tool with regards to patient outcomes. | Acute care hospital | Adult patients admitted with stroke to any of 12 participating hospital sites implementing the screening in given time frame, who were discharged alive plus control group of patients from hospitals in another country, not using the screening. | Age, gender, marital status, household income, race, insurance provider, clinical variables (comorbidity, discharge home, length of stay, 30-day readmission/death rate) | Patient records | Quantitative |
| Elderly Fallers: A Baseline Audit of Admissions to a Day Hospital for Elderly People. | 1999 | British Journal of Occupational Therapy | McIntyre, Anne | C | n/a | England | Occupational therapy | 196 | Yes- the author outlines gaps in the current evidence and the production of new rehabilitation guidelines but uncertainties in practice. | New guidelines on rehabilitation for elderly people with falls were produced, which includes for occupational therapists, but little research explores how occupational therapy services could be improved to support management of these patients. | To establish a baseline of incidence and consequences of falls in the elderly to inform on potential areas for improvement of occupational therapy services. | Hospital | Patients admitted to hospital in the given time frame, plus sub-sample of those who were elderly and presented with a fall. | Age, gender, fall status (faller or non-faller), activity undertaken prior to fall, location of fall (indoor/outdoor), specific diagnoses made, loss of consciousness, modified Barthel activities of daily living score, accommodation status (home, residential home etc), actions following fall (for example, home care provided, family/friend support provided, sleeping downstairs etc.) | Patient records | Quantitative |
| Electronic health record data extraction: Physical therapists' documentation of physical activity assessments and prescriptions for patients with chronic low back pain. | 2023 | Physiotherapy theory and practice | Wingood M and Vincenzo J and Gell N | A | A | USA | Physiotherapy | 18 | Yes- the authors cite relevant evidence but comment on the need for more research based on what happens in daily practice, and use this to situate their study. | Physical therapy intervention is known to improve outcomes for people with lower back pain. However, many research suggests this is not often prescribed, and the literature is mixed with regards to whether physiotherapists take this approach in practice. | To trial a new data capture system for documenting physical activity intervention given by physiotherapists, and use the data collection to evaluate physical activity assessment and intervention in practice. | Physiotherapy department of a hospital | Patients referred to physiotherapy in the given timeframe, with diagnoses of lower back pain. | age, gender, ethnicity, episodes of care (encounters with physiotherapist), delivery of: physical activity history assessment, physical activity assessment, physical activity intervention approach (during sessions, at home, general movement discussion, physical activity plan), physical activity intervention type (e.g. education, in-session warm ups), length of activity | Patient records | Quantitative |
| Emergency medical services versus private transport of trauma patients in the Sultanate of Oman: a retrospective audit at the Sultan Qaboos University Hospital. | 2014 | Emergency medicine journal : EMJ | Al-Shaqsi S and Al-Kashmiri A and Al-Hajri H and Al-Harthy A | A | M | New Zealand (but study is in Oman) | Paramedical science | 821 | No | Road traffic accidents are a major problem in Oman. A new emergency medicine service was developed which aimed to reduce mortality and morbidity from road traffic accidents. | To evaluate the new system and explore if there are differences between patients transported to hospital via this system, and those transported privately. | Emergency services | People involved in road traffic accidents and admitted to the hospital site. | Age, gender, ethnicity, weekend/weekday injury, time of arrival at hospital, triage (urgent, emergency, resuscitation), injury severity score, mode of transport to hospital, ICU admission state, surgical management, length of stay, outcome (mortality, discharge, transfer) | Patient records | Quantitative |
| Epidemiology and health care utilization of patients suffering from Huntington's disease in Germany: real world evidence based on German claims data. | 2019 | BMC neurology | Ohlmeier C and Saum KU and Galetzka W and Beier D and Gothe H | A | M | Germany | Mixed- Huntington's disease | 308 | Yes- the authors identify evidence gaps and comment how real-world evidence study could fill them. | People with Huntington's disease require complex management. Most research is not relevant to the German healthcare system. More needs to be known about how many people have Huntington's and how they are routinely treated. | To estimate the incidence/prevalence of Huntington's disease in Germany, how it presents and how it is routinely treated, included the costs for this. | Multiple - hospital and outpatients. | People with Huntington's disease living in Germany using insurance providers who submit to the database used | Age, gender, Huntington’s diagnosis, comorbidities, symptoms, drug prescriptions, therapies, and medical aids. | Patient records via claims database | Quantitative |
| Establishing a Multidisciplinary Head and Neck Clinical Pathway: An Implementation Evaluation and Audit of Dysphagia-Related Services and Outcomes. | 2019 | Dysphagia | Messing BP and Ward EC and Lazarus C and Ryniak K and Kim M and Silinonte J and Gold D and Thompson CB and Pitman KT and Blanco R and Sobel R and Harrer K and Ulmer K and Neuner G and Patel K and Tang M and Lee G | C | M | USA | Speech and language therapy | 63 | Yes- the authors highlight the relevant evidence and recommendations for practice but note that implementation of this in practice is challenging | People with head and neck cancer are at risk of poor swallow function which can be supported by speech therapists as part of a multi-disciplinary care team. There is good evidence that monitoring and treatment of this improves outcomes. However, implementation of the MDT pathway and incorporating speech therapy into this is challenging in routine practice. | A new integrated pathway was implemented. The study aimed to understand the barriers and facilitators to doing so successfully and evaluate the outcomes from the service. | Tertiary cancer service | Patients with head and neck cancer following the clinical pathway | Age, gender, tumour type and location, tumour stage, attendance at appointments, reasons for not attending, C reported outcomes and patient reported outcomes | Patient records | Quantitative |
| Exploring access and engagement with Improving Access to Psychological Therapies (IAPT) services, before, during, and after the COVID-19 lockdown: A service evaluation in the Northwest of England. | 2023 | Psychotherapy research : journal of the Society for Psychotherapy Research | Verbist IL and Fabian H and Huey D and Brooks H and Lovell K and Blakemore A | C | M | England | Psychology/ mental health | 13,019 | Partially- evidence gaps are identified which aim to be fulfilled but does not frame this as implementation/translation/research to practice issue that a routine data study can address. | Many people access and can benefit from IAPT services, but the COVID-19 lockdown meant service delivery had to change to telehealth. Evidence suggests this might have benefited some whilst been detrimental to others. Other factors are thought to be related to engagement with IAPT including age, ethnicity, socio-economic status and long-term conditions. However, little is known about how these might relate to telehealth-delivered therapy. | To evaluate access and engagement with IALP before, during and after COVID-19 where services switched from in person to remote and explore if there are client-factors associated with attendance. | Community mental health | People referred to IAPT services in Northwest England in the given time periods. | Age, gender, ethnicity, primary language, sexual orientation, employment status, perinatal status, long term condition status, psychotropic medication status, service area, wait time to first appointment, medium of communication, intervention intensity, attendance rates, discharge status, Patient Health Questionnaire (PHQ) scores, Generalised Anxiety Disorder scale (GAD) scores, time of access (pre-lockdown, during lockdown, after lockdown). | Patient records | Quantitative |
| Exploring Mental Health Presentations in Remote Aboriginal Community Controlled Health Services in the Kimberley Region of Western Australia Using an Audit and File Reviews. | 2022 | International journal of environmental research and public health | Carlin E and Cox Z and Orazi K and Derry KL and Dudgeon P | M | M | Australia | Psychology/ mental health | 92 | Partially- evidence gaps are identified which aim to be fulfilled but does not frame this as an implementation/translation/research to practice issue that a routine data study can address. | First Nations people in Australia experience high levels of mental-ill health and there are known barriers to engagement with mental health services. Primary health care and mental health care initiatives are trying to respond to this - including the Aboriginal Community Controlled Health Services. Little is known about the interaction between these. | To evaluate the role of Aboriginal Community Controlled Health Services and understand more about the patients accessing these and their outcomes. | Three Aboriginal Community Controlled Health Services mental health services. | Adults identifying as Aboriginal who accessed any of the clinics presenting with mental health concerns within the given period. | Gender, age, clinic presenting to, number of interactions with clinic, characteristics of first interaction. For a subset of patient records explored in more detail this also included: documentation of mental health screening, transfer to hospital, medication and diagnosis, and patient characteristics (alcohol or drug use, family/other violence, family/partner conflict, family member assault, imprisonment, food insecurity, guardianship order, housing, complex health), other health providers involved in care. | Patient records | Mixed |
| Factors influencing the amount of therapy received during inpatient stroke care: an analysis of data from the UK Sentinel Stroke National Audit Programme. | 2020 | Clinical rehabilitation | Gittins M and Vail A and Bowen A and Lugo-Palacios D and Paley L and Bray B and Gannon B and Tyson S | A | A | England | Mixed- stroke rehabilitation | 94905 | Yes- the authors acknowledge clinical guidance based on evidence but note that it is rarely achieved in practice. | Stroke patients rarely receive the required amount of therapy, which may be impacted by staffing levels and service organisation. A national audit of these is undertaken regularly in the UK. | To explore variations in the amount of therapy that stroke patients receive and identify factors which might be associated with this. | Hospital | People with stroke admitted to hospital in England in the given timeframe. | Age, gender, ethnicity, social deprivation, comorbidities, premorbid disability, type of stroke, stroke severity, degree of impairment, number of treating teams, time since stroke, day of the week and time of day of admission, therapy assessment conducted, minutes of therapy per day, organisation-related factors (thrombolysis service, average length of stay, type of team, number of staff, number of disciplines available 6-7 days per week, access to other teams) | Patient records | Quantitative |
| Factors related to hospital utilisation for people living with schizophrenia: Examining Allen's Cognitive Level Scores, recommended supports and routinely collected variables. | 2019 | Australian occupational therapy journal | Stewart K and Hancock N and Stancliffe RJ | M | M | Australia | Occupational therapy | 150 | Partially- evidence gaps are identified which aim to be fulfilled but does not frame this as an implementation/translation/research to practice issue that a routine data study can address. | People with schizophrenia are highly likely to be hospitalised. There is evidence that cognition levels of people with schizophrenia can help discharge planning and formulation of recommendations. The Allen Cognitive Level Screen (ACLS) supports this. Successful support in the community may reduce hospital admittance. There is less evidence about whether these recommendations are reliable sources for occupational therapists to identify and implement needs. | To explore what factors are associated with hospital use by people with schizophrenia including whether cognitive assessment and recommendation formulation (using the ACLS) post-discharge have an influence. | Hospital - mental health units | People with schizophrenia who were admitted to the hospital in the given time frame, and who had an ACLS assessment by an occupational therapist. | Age, gender, comorbidities, discharge accommodation type, living situation, support at home, ACLS score, match between ACLS-recommendations and actual support, mandated mental health outcome measures (Life-Skill Profile-16, Health of a Nation Outcome Scale, Kessler-10.), re-presentation at hospital and number of times, readmission and number of times, number of days between discharge and re-presentation, total number of days of re-admittance(s) | Patient records | Quantitative |
| French registry of cases of type I acute aortic dissection admitted to a cardiac rehabilitation center after surgery. | 2009 | European journal of cardiovascular prevention and rehabilitation : official journal of the European Society of Cardiology, Working Groups on Epidemiology & Prevention and Cardiac Rehabilitation and Exercise Physiology | Corone S and Iliou MC and Pierre B and Feige JM and Odjinkem D and Farrokhi T and Bechraoui F and Hardy S and Meurin P | PS | PS | France | Physiotherapy | 33 | Partially - the authors acknowledge that recent research and practice recommendations exist for rehabilitation of type 2 dissection, but some pertinent questions that would impact their implementation/success have not been addressed, and that understanding the heterogeneity of patients with type 1 dissection complicates research. | Patients who have had aortic dissection type 1 routinely receive and benefit from physical training programmes, however type 1 patients with more complex issues face a risk that physical programmes may aggravate their lesions. The benefit/risk ratio for these patients in unknown. | To evaluate routine cardiac rehabilitation practices and report on incidences for patients following type 1 dissection. | Rehabilitation centre | Patients with referred to cardiac rehabilitation centres who had type 1 dissection and a patent false lumen. | Gender, age, presence of Marfan syndrome, post-surgery complications, Arch surgery, pre-surgical hypertension treatment, aortic diameter (from CT scans), surgical intervention, number of training sessions attended, length of hospital stay, blood pressure (at rest and maximum during training), intensity of exercise, mortality at 12 months, return to work at 12 months. | Patient records | Quantitative |
| Graded exercise therapy for chronic fatigue syndrome: an audit. | 2001 | Physiotherapy | PD, White and VAB, Naish | C | M | England | Physiotherapy | 92 | Yes- the authors highlight from randomised controlled trials but comment that there is little evidence about how the tested treatments really far in real-life clinical settings, and discuss the role of benchmarking against RCT results - their study is situated in this context. | There is lots of evidence which demonstrates that graded exposure therapy (GET) benefits people with chronic fatigue syndrome (CFS)/ myalgic encephalomyelitis (ME), but little is known about the effectiveness in 'real life' clinical settings. | To examine the effectiveness of GET in an everyday clinical environment and compare the effect sizes observed with that seen in randomised controlled trials. | Tertiary care - specialist service within hospital | People with CFS/ME referred into service within time period | Age, gender, ethnicity, marital status, education level, Outcome measures (Chalder Fatigue Questionnaire, Work and Social Adjustment Scale, Short Form Health Survey, Hospital Anxiety and Depression Scale), number of sessions (and dropout rate). | Patient records | Quantitative |
| Greater trochanteric pain syndrome in the UK National Health Service: A multicentre service evaluation. | 2019 | Musculoskeletal care | Stephens G and O'Neill S and Clifford C and Cuff A and Forte F and Hawthorn C and Littlewood C | M | M | England | Physiotherapy | 162 | Yes- the authors critique existing evidence and the study is specifically aiming to evaluate research to practice gaps. | People with GTPS may benefit from physiotherapy programmes but most research is based on people not typically representative of those who present at services. Lots of personal and clinical characteristics may influence healthcare-seeking behaviours. | To examine patient characteristics to understand more about who seeks healthcare support for GTPS to examine whether findings from randomised controlled trials can be applied to them. | Mixed- outpatients services, general hospital, elective hospital | Patients presenting with lateral hip pain and diagnosis of GTPS to the participating sites within the timeframe. | Age, gender, hip pain (unilateral, bilateral), BMI, duration of symptoms, pain levels at assessment (Visual Analogue Scale), health related quality of life (EQ5D, MSK-HQ), Problems identified (e.g. walking, sleeping), symptoms, medical histories, medication. | Patient records | Quantitative |
| Has the quality of physiotherapy care in patients with Whiplash-associated disorders (WAD) improved over time? A retrospective study using routinely collected data and quality indicators. | 2018 | Patient preference and adherence | Oostendorp RA and Elvers H and van Trijffel E and Rutten GM and Scholten-Peeters GG and Heijmans M and Hendriks E and Mikolajewska E and De Kooning M and Laekeman M and Nijs J and Roussel N and Samwel H | M | M | Netherlands | Physiotherapy | 810 | Yes - the authors comment on the traditional evidence and practice recommendations and propose looking at routinely collected data to offer an advantageous way to understand treatment quality and management. | Clinical practice guidelines outline specific steps to follow in clinical scenarios. These can be used as quality indicators. Little is known about the quality of physiotherapy for whiplash-associated disorders in real clinical practice. | To develop new quality indicators based on guidelines and examine routine data to evaluate physiotherapy quality across time. | Physiotherapy centres | People with whiplash associated disorders who presented to the participating physiotherapy sites. | Age, gender, educational level, employment status, referral source, accident characteristics (direction of impact, collision, trauma, onset of complains, pre-existing health status), current health, activity and participation, Duration between accident and assessment, prior diagnostics and treatment (imaging, cervical collar, medication, physiotherapy), type of therapy (manual, massage, exercise etc), clinical complains and symptoms, type of whiplash associated disorder, Range of clinical indicators (e.g. pain behaviour, Waddell’s signs, coping, fear avoidance, neurological outcomes, walking test etc). | Patient records | Quantitative |
| Holistic therapy with disabled adults from a social and individual perspective: A service evaluation feasibility study. | 2017 | Counselling & Psychotherapy Research | Halacre, MÃ¨lani and Jalil, Rahul | CB | M | England | Psychology/ mental health | 91 | Yes - the authors comment that people with disabilities and mental health needs are not well researched, though disability studies indicate a wealth of suggestions for health services to adhere to. They identify this gap in research to practice and situate the study directly within it. | People with disabilities benefit from mental health support. Health professionals show bias towards people with disabilities and typical services are ill-adapted to support people with disabilities to attend and engage. | Evaluate a service which employs disabled therapists to provide therapy to people with disabilities | Mental health organisation (independent charity) | Adults with a disability and mental health needs who were referred into the service for therapy. | Age, gender, ethnicity, marital/relationship status, religious faith, disability type (physical impairment, chronic physical illness, sensory disability, presenting mental health issue, wait time between referral and initial assessment, number of sessions attended, psychological distress measures (pre and post therapy), therapy completion, reasons for non-attendance, discharge process used. | Patient records | Quantitative |
| How do patients pass through stroke services? Identifying stroke care pathways using national audit data. | 2020 | Clinical rehabilitation | Gittins M and Lugo-Palacios DG and Paley L and Bray B and Bowen A and Vail A and Gannon B and Tyson S | M | M | England | Mixed- stroke rehabilitation | 94905 | Partially - the authors acknowledge that recommendations exist for specific stages of the stroke pathway but there is a gap in that little research or guidelines addresses looking at stroke services across the pathway. | Organisation and configuration of stroke service across pathways and how this may impact patient outcomes has received little research attention. Understanding different configurations and how they relate to patient rehabilitation needs may support service improvement. | To evaluate utilisation of stroke care services across the pathway to determine 'service routes' in relation to patient needs and outcomes, and costs. | Hospital and post-hospital discharge services | Patients admitted to hospital with stroke in the given timeframe | Age, gender, ethnicity, social deprivation (quartiles), stroke severity (NIHSS), type of stroke, therapy needs (physiotherapy, occupational therapy, speech and language therapy, psychology), minutes of inpatient therapy per day, minutes of community therapy per day, length of inpatient stay, independence score (pre-morbid and post-stroke), mortality, pathway allocated, estimated cost associated with pathway. | Patient records | Quantitative |
| How Successful are We at Getting our Clients Back to Work? The Results of an Audit. | 2004 | British Journal of Occupational Therapy | Brewin, Jane and Hazell, Alexa | C | C | England | Occupational therapy | 16 | Partially- evidence gaps are identified which aim to be fulfilled but does not frame this as implementation/translation/research to practice issue that a routine data study can address. | People with acquired neurological disorders or chronic, fluctuating disorders often have a goal to return to work but current services to support them to meet this are inadequate and could be improved by multi-agency working including occupational therapy. A new service embedding this was set up, and some recommendations for practice were developed and implemented. | To evaluate the service in terms of the progress made in returning to work. | Community rehabilitation service/ outpatients | People with head injury or chronic condition referred to occupational therapy with a goal of returning to work. | Reason for referral, occupation at time of injury/illness, Age, gender, return to work status following rehabilitation, occupational changes, work activity engagement (in work, not in work), inter agency work adherence. | Patient records | Quantitative |
| Imagery-Focused Cognitive Therapy (ImCT) for Mood Instability and Anxiety in a Small Sample of Patients with Bipolar Disorder: a Pilot Clinical Audit. | 2018 | Behavioural and cognitive psychotherapy | Hales SA and Di Simplicio M and Iyadurai L and Blackwell SE and Young K and Fairburn CG and Geddes JR and Goodwin GM and Holmes EA | M | M | England | Psychology/ mental health | 11 | Partially- evidence gaps are identified which aim to be fulfilled but does not frame this as implementation/translation/research to practice issue that a routine data study can address. | The evidence around therapies for bipolar disorder is conflicting and contentious. There has also been little attention paid to the role of anxiety in therapy and treatment for people with bipolar disorder. A service was set up with this in mind. | To audit the service with respect to the patients referred and their presenting problems and treatment outcomes to refine the service/treatment offered. | Tertiary mental health service | Patients with bipolar disorder referred into the service not receiving other treatments. | Age, gender, ethnicity, employment status, marital status, diagnoses (type 1 or type 2), presenting clinical problems, medication, Quick Inventory of Depressive Symptomatology Self-Report (QIDS-SR) scores, the Altman Self-Rating Scale for Mania (ASRM) scores, Beck Anxiety Inventory scores, Patient Experience Questionnaire scores, number of manic and depressive episodes at baseline and follow-up, length of episode, number of sessions offered, number of sessions attended, reasons for declining service or non-attendance. | Patient records | Quantitative |
| Implementing circuit class training can increase therapy time and functional independence in people with stroke receiving inpatient rehabilitation: findings from a retrospective observational clinical audit. | 2023 | Physiotherapy theory and practice | McDonell I and Barr C and van den Berg M | M | M | Australia | Physiotherapy | 110 | Yes- the authors acknowledge findings from randomised controlled trials and identify that it is unknown whether the positive effects seen in these are replicated in a real-world implementation of the treatment programmes and situate the study in this context. | Research indicates that circuit class therapy (CCT) provided in groups, as an alternative to individual 1-2-1 therapy, is a useful intervention for people with stroke and can be efficient in terms of staff time. It is not known whether CCT in inpatient rehabilitation settings has similarly positive effects. | To evaluate a new CCT service in an inpatient rehabilitation setting in terms of therapy time and patient outcomes. | Inpatient rehabilitation unit | People with stroke admitted to the hospital site in the given timeframe. | Age, gender, type of stroke, stroke severity, stroke location, cognitive skills on admission, time between stroke onset and admission to unit, functional independent measure scores, treatment group | Patient records | Quantitative |
| Improved quality of physiotherapy care in patients with Whiplash-Associated Disorders: Results based on 16 years of routinely collected data. | 2022 | Frontiers in pain research (Lausanne, Switzerland) | Oostendorp RAB and Elvers H and van Trijffel E and Rutten GM and Scholten-Peeters GGM and De Kooning M and Laekeman M and Nijs J and Roussel N and Samwel H | M | M | Netherlands | Physiotherapy | 810 | Yes- the authors position use of routinely collected data to bridge the research to practice gap. | Whiplash associated disorders are highly prevalent and highly complex to manage. Quality improvement is central to a physiotherapist’s role. By examining routinely collected data, quality indicators can be developed, and improvements and advancements in care can be examined. | To synthesise recent reports on use of physiotherapy routine data which has helped develop quality indicators and test quality of care over a period. | Physiotherapy primary care | People referred to physiotherapy with whiplash associated disorders | Items of diagnostic clinical reasoning process adhered to (year of referral, referral source, time since accident, request for care, age, gender, educational level, employment status, accident information, type and location of trauma, functional problems experienced, pre-existing health status, recovery information, prognostic factors (Waddell's sign, use of coping, fear avoidance), recovery rate, musculoskeletal test scores, psychological test scores, health profile alignment. | Patient records | Quantitative |
| Indicators of adherence to physiotherapy attendance among Saudi female patients with mechanical low back pain: a clinical audit. | 2010 | BMC musculoskeletal disorders | Al-Eisa E | A | N/a | Saudi Arabia | Physiotherapy | 98 | Partially- evidence gaps are identified which aim to be fulfilled but does not frame this as an implementation/translation/research to practice issue that a routine data study can address. | Lower back pain physiotherapy known to have benefits. Greater benefits are seen with greater therapy adherence. Women in Saudi Arabia have social restraints which may affect women's health and adherence to treatments. Adherence is also influences by social and cultural factors. | To explore factors related to treatment adherence of women from Saudi Arabia with lower back pain. | Physiotherapy department of tertiary hospital. | All female Saudi patients referred to physiotherapy due to lower back pain | Age, marital status, occupation, time from referral to physiotherapy visit, number of sessions attended, adherence rate, pain improvement report, medical condition, duration of pain, diagnosis, first/recurrent episode, other medical problems | Patient records | Quantitative |
| Inequity in physiotherapeutic interventions for children with Cerebral Palsy in Sweden-A national registry study. | 2020 | Acta paediatrica (Oslo, Norway : 1992) | Degerstedt F and Enberg B and Keisu BI and BjÃ¶rklund M | A | A | Sweden | Physiotherapy | 2855 | No | Inequities in healthcare have been documented in Sweden which may be due to gender or country of birth, or where they live. Physiotherapy inequities have also been documented. | To investigate physiotherapy provision to children with cerebral palsy across Sweden and identify whether there are any inequities in relation to gender, country of birth, and geographical region. | Physiotherapy services | Children with cerebral palsy accessing rehabilitation centres submitting data to the registry | Gender, country of birth, geographical region, physiotherapy received (services received, frequent treatment, intensive training, frequent supervision) | Patient records | Quantitative |
| Inpatient rehabilitation therapy in stroke patients with reperfusion therapy: a national prospective registry study. | 2023 | BMC neurology | Li S and Lu Y and Fang S and Wang L and Peng B | M | M | China | Mixed- stroke rehabilitation | 209189 | Yes - the authors acknowledge that although evidence indicates best practice, little is known about ‘real' clinical practice in China and situate their study within this. | There is good evidence supporting inpatient rehabilitation approaches for stroke. There are challenges in China for implementing post-stroke rehabilitation and little is known about how much is provided and received, and the outcomes from it. | To determine the extent of inpatient rehabilitation therapy in China and explore the factors associated with it. | In patient stroke units in China | People with stroke and reperfusion therapy admitted to the unit. | Age, gender, nationality, region, hospital level, BMI, type stroke, Reperfusion therapy, reperfusion time, Stroke severity score (NIHSS - initial, 24 hours), modified Rankin scale (mRS - initial, 24 hours), length of stay, haemorrhage, COVID-19 status, rehabilitation therapies received (traditional rehabilitation, acupuncture or massage, physical therapy, occupational therapy, speech therapy, other rehabilitation, other treatments based on traditional Chinese medicine) | Patient records | Quantitative |
| Intensive physiotherapy for vegetative and minimally conscious state patients: a retrospective audit and analysis of therapy intervention. | 2013 | Disability and rehabilitation | Wheatley-Smith L and McGuinness S and Colin Wilson F and Scott G and McCann J and Caldwell S | C | M | Northern Ireland | Physiotherapy | 10 | Partially- evidence gaps are identified which aim to be fulfilled but does not frame this as an implementation/translation/research to practice issue that a routine data study can address. | Physiotherapy intervention can reduce contractures and consequences of spasticity in people with a range of difficulties. There is little evidence about intervention for contractures in minimally conscious patients with acquired brain injuries and the effects are unknown. | To explore physiotherapy interventions used in practice with the given patient group, including effectiveness of managing contractures. | In patient brain injury rehabilitation service | Patients admitted to the unit in 'low awareness states'. | Age, gender, Injury to admission duration in days, length of stay, Wessex Head Injury Matrix (WHIM) scores (pre and post), primary diagnosis, Physiotherapy intervention detail (goals set, goals achieved), stretching movements (passive, manual, limb area), period of stretching intervention (weeks), casting/splinting intervention (limb area), period of casting/splinting intervention (days), Botox intervention (limb area), Botox detail (cycles and dosage), Standing intervention (number of weeks, type of intervention), patient dependence levels, joint contractures. | Patient records | Quantitative |
| Interventions for Driving Disruption in Community Rehabilitation: A Chart Audit. | 2023 | Disability and rehabilitation | Marnane K and Gustafsson L and Liddle J and Molineux M | M | A | Australia | Occupational therapy | 80 | No | Many people entering community rehabilitation services will be 'driving disrupted'. The extent of intervention given in such settings is not known. | To identify how often community rehab patients experience 'driving disruption' and document the current practices in supporting it. | Community rehabilitation service | Patient who received a full programme of community rehabilitation | Age, gender, number of days in hospital, diagnosis, driving status (on admission and discharge), driving assessment conducted, driving intervention provided, professional providing intervention, onward services/discharge. | Patient records | Quantitative |
| Investigating the association between inpatient stroke therapy and disability, destination on discharge, length of stay and mortality: a prospective cohort study using the Sentinel Stroke National Audit Programme. | 2022 | BMJ open | Gittins M and Lugo-Palacios DG and Vail A and Bowen A and Paley L and Bray B and Gannon B and Tyson S | A | A | England | Mixed- stroke rehabilitation | 94905 | Partially- evidence gaps are identified which aim to be fulfilled but does not frame this as an implementation/translation/research to practice issue that a routine data study can address. | It is generally accepted that more therapy leads to better outcomes in stroke rehabilitation. Most evidence is based on chronic patients/community settings. There is insufficient evidence guiding the relationship between the amount of therapy and outcomes in the acute rehabilitation context. | To investigate the relationship between amount of therapy in inpatient rehabilitation stage and patient outcomes. | Hospitals / stroke units | People with stroke admitted to hospital in the given time frame | Age, gender, ethnicity, Stroke severity on admission (NIHSS), Co-morbidities, Stroke type, socio-deprivation, health outcomes at discharge (Disability status, mortality, discharge setting, length of inpatient stay), Requirement of each therapy, admission to therapies, Number of days of therapy (and % of days of time spent receiving therapy), duration of therapy (per session and per stay) | Patient records | Quantitative |
| Lee Silverman Voice Treatment for people with Parkinson's: audit of outcomes in a routine clinic. | 2015 | International journal of language & communication disorders | Wight S and Miller N | C | M | England | Speech and language therapy | 33 | Yes- the authors acknowledge the evidence base for the approach but identify its limitations in that the intervention has not been trialled from a real-world busy clinical setting and situate their study within this. | Lee Silverman Voice Therapy (LSVT) had been shown to be an effective intervention for people with Parkinson's disease in controlled trials which have a highly favourable context. The outcomes measured in these studies are often acoustic measures. It is not known how effective LSVT is when implemented in routine practice, and in relation to more functional measures. | To explore the effectiveness of LSVT in a busy routine clinic. | Outpatient service | People with Parkinson's disease referred into the service who had been recommended to receive LSVT by the therapist. | Age, gender, years since diagnoses, Hoehn-Yarh baseline (PD stage), Cognitive status, Follow-up attendance (attended, declined and reason), Sound pressure level (sustained phonation of vowel, reading, monologue), Voice Handicap Index scores, visual analogue scores, | Patient records | Quantitative |
| Lessons from the first two years of a new out-of-hospital airway registry in New South Wales. | 2023 | Paramedicine | Nichols, Martin and Fouche, Pieter Francsois and McPherson, Thomas and Evens, Tom and Bendall, Jason | C | C | Australia | Paramedical science | 872 | Partially - the authors acknowledge that more data is needed, and a registry was set up in response to this. | Intubation by paramedics can be controversial and the evidence-base is conflicting as to whether this should be performed by paramedics or physicians when necessary. | To explore use of intubation by paramedics, predictors of success and success rates, and evaluate how this changed over time. | Emergency services | Patients attended to by paramedics who had an attempt of intubation in the given time period. | Age, gender, weight, medical condition, indication for airway management, patient factors associated with airway difficulty, airway interventions applied prior to advanced airway management, location of intubation attempt, airway positioning manoeuvre, type of advanced airway attempt (endotracheal intubation or iGel), Success rate, Patient posture during attempt, access to airway, size of tube used, use of an airway assistant, grade of airway view, use of laryngoscope, manoeuvres used in laryngoscopy, adjuncts used, chest compression interruption, confirmation method of success, Reason for failure, difficulties encountered | Patient records | Quantitative |
| Long-term morbidities in stroke survivors: a prospective multicenter study of Thai stroke rehabilitation registry. | 2013 | BMC geriatrics | Kuptniratsaikul V and Kovindha A and Suethanapornkul S and Manimmanakorn N and Archongka Y | C | M | Thailand | Mixed- stroke rehabilitation | 214 | No | Stroke patients are at risk of comorbidities which complicate rehabilitation. There is little research and data about the long-term effects of multimorbidity in stroke rehabilitation. | To assess incidence and risk factors of morbidities in people who have had stroke during their first year after discharge. | Across the stroke pathway - hospital and rehabilitation | People with stroke admitted to participating hospitals | Age, gender, marital status, stroke aetiology, presence of family support, underlying disease, number of underlying diseases, days from stroke onset to rehabilitation admissions, length of stay in hospital, mortality, and follow-up rate, Barthel index scores, stroke-related complications. | Patient records | Quantitative |
| Management of older patients presenting after a fall--an accident and emergency department audit. | 2006 | South African medical journal = Suid-Afrikaanse tydskrif vir geneeskunde | Kalula SZ and de Villiers L and Ross K and Ferreira M | M | M | South Africa | Mixed- falls management | 100 | No | Research demonstrates the positive impact of an interdisciplinary approach to the management and prevention of falls in older people. | To evaluate how patients presenting to the hospital site following a fall are managed. | Accident and emergency departments | People admitted to accident and emergency following a fall. | Age, gender, history of fall, vital signs, examination method, radiological tests performed, type of injury, location of fracture, referral to other services, | Patient records | Quantitative |
| Moderate and Stable Pain Reductions as a Result of Interdisciplinary Pain Rehabilitation-A Cohort Study from the Swedish Quality Registry for Pain Rehabilitation (SQRP). | 2019 | Journal of clinical medicine | Ringqvist Ã… and Dragioti E and BjÃ¶rk M and Larsson B and Gerdle B | C | M | Sweden | Mixed- pain rehabilitation | 14666 | Yes- the authors comment on the evidence behind the approach based on trials and systematic reviews but comment on the need for understanding from routine data and practice | There is good evidence to support that interdisciplinary multimodal pain rehabilitation programs (IMMRPs) support good outcomes. There is little to no research exploring effect sizes in real-world patient cohorts. | To evaluate effect sizes of IMMRPs on patient outcomes in the short and long term and explore whether outcome can be predicted by initial self-reported pain measures. | Rehabilitation centre | Adults with complex chronic non-malignant pain who were referred to the specialist units/services | Age, gender, educational level, country or birth, Self-reported pain duration, persistent pain duration, number of days taken off work, pain distribution (location), number of areas with pain and Pain Region Index, psychometric measures, pain intensity, Multimodal Pain Inventory, psychological distress, Short Forum Health Survey, European Quality of Life Instrument (EQ5D), change in pain self-report. | Patient records | Quantitative |
| Nasal chondromesenchymal hamartoma in children with pleuropulmonary blastoma--A report from the International Pleuropulmonary Blastoma Registry registry. | 2010 | International journal of pediatric otorhinolaryngology | Priest JR and Williams GM and Mize WA and Dehner LP and McDermott MB | C | M | USA | Biomedical sciences | 625 | No | Nasal chondromesenchymal hamartoma (NCMH) is a rare presentation and can be associated with Pleuropulmonary blastoma (PPB). They have been described collectively in the literature. | To describe NCMH occurring in PPB patients to demonstrate that NCMH is a distinctive set of diseases associated with PPB. | Haematology Department. | Children with PPB who also developed NCMH | Age, gender, NCMH symptoms, age of NCMH diagnosis, Radiological findings, NCMH type (Unilateral or bilateral), extent of NCMH, treatment provided, age of follow-up assessment, Age at PPB diagnosis, PPB type, Other PPB findings, CT scans, histology specimens. | Patient records | Quantitative |
| National stroke audit: a tool for change? | 2001 | Quality in health care : QHC | Rudd AG and Lowe D and Irwin P and Rutledge Z and Pearson M | PS | PS | England | Mixed- stroke rehabilitation | 5589 | Partially- evidence gaps are identified which aim to be fulfilled but does not frame this as an implementation/translation/research to practice issue that a routine data study can address. | A series of audits into stroke services in England have been undertaken. Examining the quality and organisation of stroke services can be achieved through conducting audits but improvement depends on development and implementation of actions. | To examine changes to stroke services and their organisation taking place between two audit periods accompanied by a dissemination strategy to motivate change. | Hospital | People admitted to hospital with a stroke in the given timeframe | Age, gender, mortality, Bartel index (before stroke and after discharge), length of stay, length of stay in rehabilitation unit/general ward, organisational information about the treating hospital trust. | Patient records plus questionnaire | Quantitative |
| Obesity is associated with more disability at presentation and after treatment in low back pain but not in neck pain: findings from the OIOC registry. | 2016 | BMC musculoskeletal disorders | Wertli MM and Held U and Campello M and Schecter Weiner S | M | M | Switzerland | Physiotherapy | 739 | Partially- evidence gaps are identified which aim to be fulfilled but does not frame this as an implementation/translation/research to practice issue that a routine data study can address. | Obese people are at risk of developing spinal pain. Different approaches to treating spinal pain exist though not much is known about recovery and disability experienced with spinal pain. | To identify if there is an influence of body weight on level of disability and functionally related outcomes experienced by people with spinal pain. | Outpatients’ physiotherapy | People referred to physiotherapy services with neck pain or lower back pain. | Age, gender, BMI, relationship status, educational status, insurance type, occupation, work status, work status related to pain complaint, Pain type (acute, subacute, chronic), Oswestry Disability Index scores, Neck disability index scores, Fear avoidance beliefs questionnaire score, baseline pain score, treatment completion, | Patient records | Quantitative |
| Occupational therapy and vocational rehabilitation: an audit of an outpatient occupational therapy service. | 2006 | British Journal of Occupational Therapy | L, Main and J, Haig | C | C | Scotland | Occupational therapy | 118 | No | Occupational therapists are well-placed to offer vocational rehabilitation when there is not a specialist, although staffing levels need to be considered within the occupational therapy outpatient teams if this specialist area is incorporated. | To evaluate the vocational rehabilitation within an outpatient occupational therapy service to determine the demand for such interventions and their effectiveness. | Occupational therapy outpatients | People who required return-to-work interventions who were referred to the outpatient therapy service in the given time frame. | Age, gender, diagnosis, pre-injury employment status, employment status at time of initial assessment, employment status at discharge, type of occupational therapy intervention given, referral to other services. | Patient records | Quantitative |
| Oro-nasal fistula development and velopharyngeal insufficiency following primary cleft palate surgery--an audit of 148 children born between 1985 and 1997. | 2005 | British journal of plastic surgery | Inman DS and Thomas P and Hodgkinson PD and Reid CA | C | C | England | Speech and language therapy | 148 | No | Treatment for children born with cleft lip and/or palate (CLP) were found to be substandard. Specialist centres were created to improve this. | To evaluate patient outcomes from the local specialist unit. | Tertiary CLP service | Children born with CLP and accessing the specialist unit. | Age, gender, CLP diagnosis, age at operation, operating surgeon, date of operation, fistula status, fistula repair age, date of closure, surgeon of closure, pharyngoplasty status, type of pharyngoplasty, surgeon for pharyngoplasty, age at pharyngoplasty, age last seen in clinic, date last seen in clinic, speech therapy data (hyper-nasal and hypo-nasal resonance, audible nasal emission, nasal turbulence, grimace, voice-speech pattern, intelligibility) | Patient records | Quantitative |
| Orthopaedic triage at a physiotherapist-led 'Musculoskeletal Assessment Clinic': a seven-month service evaluation of outcomes. | 2014 | Irish journal of medical science | O'Farrell S and Smart KM and Caffrey A and Daly O and Doody C | A | M | Ireland | Physiotherapy | 714 | No | Physiotherapist-led triage clinics for orthopaedic outpatients have been found to be appropriate and effective. A new service utilising this approach was set up. | To describe the patients attending the service and their clinical outcomes, to identify how many were independently managed by physiotherapists and calculate how many physiotherapist referrals to an orthopaedic consultant convert to orthopaedic interventions. | Outpatients - orthopaedic and physio | People with orthopaedic complaint referred to the outpatient service. | Age, gender, employment status, diagnosis, symptoms, symptom duration, outcome at triage, outcome following orthopaedic consultant if relevant, medical investigations requested. | Patient records | Quantitative |
| Paediatric out-of-hospital cardiac arrests in Melbourne, Australia: improved reporting by adding coronial data to a cardiac arrest registry. | 2013 | Emergency medicine journal : EMJ | Deasy C and Hall D and Bray JE and Smith K and Bernard SA and Cameron P | M | M | Australia | Paramedical science | 301 | Partially- evidence gaps are identified which aim to be fulfilled but does not frame this as an implementation/translation/research to practice issue that a routine data study can address. | Cardiac arrests in children are rare and the underlying aetiology is most accurately identified through autopsy. Understanding aetiologies is helpful for influencing management/treatment. There is little research into autopsy findings and data about the cardiac arrests. | To link cardiac arrest data with coronary data and use this to develop understanding aetiologies. | Emergency services | Children who had experienced non-traumatic out-of-hospital cardiac arrest in the locality and time frame. | Age, gender, known cardiac condition, cardiac rhythm on arrival, discharge status/mortality, paramedic-report precipitant of arrest, coronial cause of death, | Patient records | Quantitative |
| Paediatric pelvic imaging: improvement in gonad shield placement by multidisciplinary audit. | 2001 | Pediatric radiology | McCarty M and Waugh R and McCallum H and Montgomery RJ and Aszkenasy OM | C | C | England | Radiography/Radiotherapy | 218 | No | Gonad shielding is essential for minimising radiation dose during radiographic imaging. Concerns had been raised in the literature and locally that placement of gonad shields was inappropriate, leading to repeat radiographs being required. | To assess local practices with regards to gonal shield placement and repeat scan requests. | Radiography department in a hospital | Children who had undergone pelvic radiography in the given time frame. | Age, gender, number of films made, presence of gonad shield, appropriateness of gonad shield device, appropriateness of position of gonad shield. | Patient records | Quantitative |
| Parents with learning disabilities and speech and language therapy. A service evaluation of referrals and episodes of care. | 2012 | British Journal of Learning Disabilities | Stansfield, Jois | C | C | England | Speech and language therapy | 124 | Partially- evidence gaps are identified which aim to be fulfilled but does not frame this as an implementation/translation/research to practice issue that a routine data study can address. | Parents who have a learning disability face many challenges which can be underpinned by communication. Communication success can be supported by speech and language therapists. The local learning disability team noted referral into the service was increasingly related to parenting/risk of harm to children. | To explore referral patterns into speech and langauge therapy learning disability team that related to parenting to identify the level of demand. | Community learning disability team | Adults with a learning disability that were newly referred into speech and language therapy services | Age, gender, parent status (y/n), number of children if parent, age of children if parent, living status of children (with parents, with guardian, adopted etc), referral reason, case management stage, referral form written notes. | Patient records | Mixed methods |
| Patient and Prehospital Predictors of Hospital Admission for Patients With and Without Histories of Diabetes Treated by Paramedics for Hypoglycaemia: A Health Record Review Study. | 2023 | Prehospital Emergency Care | Sinclair, Julie E. and Austin, Michael A. and Leduc, Shannon and Dionne, Richard and Froats, Mark and March and , Jane and Vaillancourt, Christian | O | M | Canada | Paramedical science | 791 | Partially- evidence gaps are identified which aim to be fulfilled but does not frame this as an implementation/translation/research to practice issue that a routine data study can address. | Diabetic emergencies of people with hypoglycaemia represent a considerable proportion of paramedics annual cases, and a vast volume of patients treated in emergency departments arrive by ambulance. In Canada, paramedics can treat hypoglycaemia on-site and new pathways have been set up to ensure appropriate triage and reduce the periods in which no ambulances are available. | To describe the patients treated by paramedics and taken to hospital for their hypoglycaemia with regards their clinical characteristics, the management and their outcomes | Emergency services | Adults assessed by paramedics with hypoglycaemia within the time frame. | Age, gender, medical history, medication, initial vital signs, diabetes status, hospital admission, emergency management, diagnoses, patient disposition, treating paramedic's scope of practice, pick-up location, diagnostic impression, paramedic intervention, final vital signs, dispatch code and priority, response intervals and for patients admitted to hospital, this also included: length of stay, final diagnosis, final vital status | Patient records | Quantitative |
| Patient unable to express why he was on the floor, he has aphasia.' A content thematic analysis of medical records and incident reports on the falls of hospital patients with communication disability following stroke. | 2023 | International Journal of Language & Communication Disorders | Sullivan, Rebecca and Hemsley, Bronwyn and Harding, Katherine and Skinner, Ian | M | M | Australia | Speech and language therapy | 72 | Yes - the authors acknowledge research in the area but report that often it excludes people with communication disability and situate their study in the context of this. | People with communication difficulties are often excluded from research around falls and patient safety. People with communication difficulties may experience difficulties following instructions about safe transfers and falls prevention. Medical records outlining falls may provide information on whether communication difficulties influenced a fall. | To examine medical records about falls to identify whether communication and communication disability was a factor that was mentioned. | Sub-acute rehabilitation ward in hospital | Patients with stroke admitted to ward, who have also acquired a communication disability from the stroke. | Age, gender, length of stay, Functional Independent Measure (FIM) on admission, number of falls, type of stroke, type of communication difficulties, speech and language therapy assessment used, text-based medical records or incident reports relating to falls. | Patient records | Qualitative |
| Patient-focused goal planning process and outcome after spinal cord injury rehabilitation: quantitative and qualitative audit. | 2012 | Clinical rehabilitation | Byrnes M and Beilby J and Ray P and McLennan R and Ker J and Schug S | A | M | Australia | Mixed- spinal injury rehabilitation | 100 | Partially- evidence gaps are identified which aim to be fulfilled but does not frame this as an implementation/translation/research to practice issue that a routine data study can address. | Multidisciplinary goal planning for spinal rehabilitation is supported by evidence. There is limited research about the processes involved in this, and how patients adjust through it. | To evaluate a goal planning programme with regards to the shift in psycho-social and physical functioning of patients, and their satisfaction and experiences of it. | Specialist rehabilitation unit. | Patients in the rehabilitation unit with spinal cord injury. | Age, gender, relationship status, educational level, length of inpatient stay, cause of injury, consequence of injury, Needs Assessment Checklist (NAC) goals set and scores at admission and discharge, qualitative sections of NAC pertaining to patient perceptions | Patient records | Mixed methods |
| Patient-reported mental health and well-being trajectories in oncology patients during radiation therapy: an exploratory retrospective cohort analysis using the Ontario Cancer Registry. | 2023 | Quality of life research : an international journal of quality of life aspects of treatment, care and rehabilitation | Kwon JY and Kopec J and Sutherland JM and Lambert LK and Anis AH and Sawatzky R | A | A | Canada | Psychology/ mental health | 3416 | Partially- evidence gaps are identified which aim to be fulfilled but does not frame this as an implementation/translation/research to practice issue that a routine data study can address. | People undergoing radiation therapy may experience distress and impact their mental health. Little is known about how personal factors may relate to these. Understanding more about this can improve patient care and their experiences. | To explore the varied trajectories of anxiety and depression symptoms in people undergoing radiation therapy and explore the association of these with socio-demographic factors, physical symptoms, and clinical variables. | Cancer registry | Patients with cancer undergoing radiation therapy in the sites contributing to the registry | Age, gender, income, immigration status, rurality, cancer diagnosis, comorbidities, Edmonton Symptom Assessment System—revised (ESAS-r) scores over time. | Patient records | Quantitative |
| Pelvic girdle pain - part 1: quantitative results from a mixed-methods service evaluation introducing a manual therapy treatment approach to usual care. | 2016 | Journal of Pelvic, Obstetric & Gynaecological Physiotherapy | Monaghan, C. and Haywood, A. | M | M | England | Physiotherapy | 46 | Partially- the authors comment that given the paucity of conclusive research in the area, practice guidance indicates physiotherapists should take a cautious theoretical approach, but do not explicitly refer to the value of routine data in evaluating this. | There is inconclusive evidence about the best approach to manual therapy for pelvic girdle pain. In routine practice, this may be used as an adjunct to other therapies but evidence is not clear as to which is more effective. | To compare the impact of manual therapy as an adjunct to routine practice to usual care on patient outcomes. | Physiotherapy outpatient service | Antenatal women with pelvic girdle pain referred to the service. | Age, fluency in English, referral reason, Pelvic Girdle Questionnaire scores at admission and discharge, therapy group (with manual therapy or without manual therapy) | Patient records | Quantitative |
| Physician-led prehospital management is associated with reduced mortality in severe blunt trauma patients: a retrospective analysis of the Japanese nationwide trauma registry. | 2021 | Scandinavian journal of trauma, resuscitation and emergency medicine | Endo A and Kojima M and Uchiyama S and Shiraishi A and Otomo Y | C | C | Japan | Paramedical science | 30551 | Partially- evidence gaps are identified which aim to be fulfilled but does not frame this as an implementation/translation/research to practice issue that a routine data study can address. | Evidence about physician-led prehospital trauma management is conflicting due to the inclusion of helicopter medical services which may confound the results. More research is needed that excludes helicopter transfers. | To evaluate the impact of physician-led trauma management independent of hospital transfer time and identify patient characteristics which may influence outcome. | Emergency services | Patients aged 15 and above who suffered severe blunt injuries who were attended to by emergency services. | Age, gender, mechanism of injury, year of injury, season of injury, time of injury, time of physician contact, time of hospital arrival, vital signs, and consciousness level (at scene of injury and hospital arrival), injury scale score, injury severity score, survival status at discharge, pre-hospital management group (physician led or paramedic led) | Patient records via Registry | Quantitative |
| Physiotherapist as an alternative to a GP for musculoskeletal conditions: a 2-year service evaluation of UK primary care data. | 2019 | The British journal of general practice : the journal of the Royal College of General Practitioners | Downie F and McRitchie C and Monteith W and Turner H | C | C | Scotland | Physiotherapy | 8417 | Partially- evidence gaps are identified which aim to be fulfilled but does not frame this as an implementation/translation/research to practice issue that a routine data study can address. | Extended scope physiotherapists (ESPs) can provide first contact for people with musculoskeletal disorders and support the challenges facing GP services in Scotland. Whilst these services increasingly exist, there is no published evaluation of the impact of them. | To evaluate an ESP service with regards to its uptake, onward referrals and impact on GP workload. | GP practice | Patients attending GP practices referred to the ESP service directly at the site. | Patient status (new or returning), attendance at appointments, appropriateness of referral, outcome of appointment (self-management, physiotherapy advice, onward investigations), requirement for GP (review, prescription, fit note etc), referral to orthopaedic services (either GP or ESP), outcome from orthopaedic referral, referral to physiotherapy services, requirements for steroid injection in the year following appointment, patient experience scores (via questionnaire). | Patient records | Quantitative |
| Physiotherapist-Led Triage at a Rheumatology-Based Musculoskeletal Assessment Clinic: an 18-Month Service Evaluation of Activity and Outcomes. | 2019 | ACR open rheumatology | Caffrey A and Smart KM and FitzGerald O | C | C | Ireland | Physiotherapy | 508 | Partially - the authors acknowledge a research gap and situate their study in this but do not explicitly refer to a implementation gap | There is emerging evidence that clinical specialist physiotherapists (CSPs) can support triage and assessment for a range of musculoskeletal disorders, to reduce burden on consultant-led clinics. Little is known about CSPs specifically in rheumatology settings. | To evaluate the proportion of rheumatology patients who were managed by CPSs in an assessment clinic and identify trends in these patients. | Outpatients’ rheumatology clinic | Patients with rheumatology complains referred to the clinic in the given timeframe. | Age, gender, employment status, diagnosis, symptom duration, professional contact (CSP or CSP and consultant), discharge point (GP, rheumatology team, other), further rheumatology involvement (intervention, prescription, injection). | Patient records | Quantitative |
| Physiotherapist-led triage of patients with thoracic spine pain in a musculoskeletal assessment clinic: A service evaluation of activity and outcomes. | 2019 | Physiotherapy Practice & Research | Smyth, Conor and Smart, Keith and Fitzpatrick, Martina and Caffrey, Aoife and McLoughlin, Catherine and Doody, Catherine | A | M | Ireland | Physiotherapy | 88 | No | Thoracic spine pain and/or dysfunction (TSPD) can occur from a range of disorders and there be no single explicit identifiable source of pain. It has become known as a 'red flag' for malignancy, but this has been challenged. Clinical specialist physiotherapists (CSPs) can support triaging these patients. | To examine records to estimate the prevalence of TSPD and explore the diagnostic categories to evaluate the extent of patients where TSPD could be attributable to a 'serious' pathology, as well as to explore the kinds of patients presenting with TSPD and how they were clinically managed in a physiotherapist-led clinic. | Secondary care musculoskeletal triage clinic | Patients with TSPD referred to the clinic | Age, gender, TSPD diagnosis category and subcategory, investigations requested (number and type), reasons for investigation request, management pathway (CPS management, referral to consultant, discharged, discharged to physiotherapy). | Patient records | Quantitative |
| Physiotherapists with musculoskeletal training in an emergency department for patients with non-specific low back pain: A service evaluation. | 2022 | Musculoskeletal care | Davies F and Pace J and Angus M and Chan-Braddock S and Jagadamma KC | A | M | Malta | Physiotherapy | 1125 | Yes - the authors acknowledge relevant research but identify that there is a lack of evidence on the routine practice in the local context, which the study addresses. | There is a lot of evidence to support the use of physiotherapists in emergency departments. Low back pain is a common musculoskeletal (MSK) complaint presenting to emergency departments which physiotherapists may be especially well-placed to support. MSK physiotherapists have been placed in the local hospital to support demand. | To evaluate the use of MSK physiotherapists in emergency departments by comparing interventional approaches taken by physiotherapists compared with medical doctors. | Emergency department | Patients with non-specific lower back pain attending the emergency department in the given time frame. | Presenting complaint, hour of attendance at emergency department, professional seen by (physiotherapist or doctor), return rate to emergency department, referrals for x-ray made, referral to orthopaedic out-patient made, referral to physiotherapy made. | Patient records | Quantitative |
| Physiotherapy in Mount Hagen General Hospital: an audit of activity over a six-month period. | 2001 | Papua and New Guinea medical journal | Powell N | C | n/a | Papua New Guinea | Physiotherapy | 571 | Partially- evidence gaps are identified which aim to be fulfilled but does not frame this as an implementation/translation/research to practice issue that a routine data study can address. | Physiotherapy coverage is minimal in Papua New Guinea, though it is well documented that physiotherapy can benefit people. There is not much awareness at the policy level of the benefits of physiotherapy in the empirical context. | To illustrate the role of physiotherapy in a hospital by exploring the use of the service and the types of patients supported. | Physiotherapy department of a hospital | Patients referred to physiotherapy department | Age, gender, outpatient or inpatient, ward seen if inpatient, diagnosis, day/s of contact, minutes spent during contact, number of treatment sessions. | Patient records | Quantitative |
| Physiotherapy management of first nations children with bronchiectasis from remote top end communities of the northern territory: a retrospective chart audit. | 2023 | Frontiers in pediatrics | Welford A and McCallum GB and Hodson M and Johnston H | C | M | Australia | Physiotherapy | 143 | Yes- the authors comment on the evidence supporting intervention for children with bronchiectasis, and practice guidelines, but critique this in that there are specific barriers faced by First Nations children in accessing this but there has been no research on this effect. They situate their study in this context. | First Nations children experience barriers to healthcare for bronchiectasis although they are also disproportionately impacted by it. Access to services for First Nations children in remote communities is unknown. | To explore First Nation children's physiotherapy management for bronchiectasis from remote communities. | Inpatient and outpatient physiotherapy teams | First Nation children with bronchiectasis accessing service from the government healthcare facility | Age, gender, birth weight, pre-term status, immunisation record, number of hospitalisations (ever and in last 12 months), aetiology, caregiver-reported primary symptoms, inpatient management (physiotherapy intervention provided, home program provided, discharge plan, setting referred to), outpatient management (care plan, physiotherapy referral, physiotherapy intervention, respiratory exacerbations). | Patient records | Quantitative |
| Physiotherapy provision to hospitalised stroke patients: Analysis from the UK Sentinel Stroke National Audit Programme. | 2019 | European stroke journal | McGlinchey MP and Paley L and Hoffman A and Douiri A and Rudd AG | C | M | England | Physiotherapy | 306,078 | Yes- the authors comment on the current evidence but critique that is utilises small samples, which it addresses directly through is use of audit data. | Evidence supports that physiotherapy in inpatient stroke care improve patient outcomes. Less is known about which aspects of physiotherapy result in better outcomes and how different patients respond to them. | To explore associations between physiotherapy factors (relating to applicability for physiotherapy, minutes of therapy provided and intensity of therapy), patient characteristics and patient outcomes. | Hospitals/ stroke units | Patients with stroke admitted to hospital. | Age, gender, pre-morbid modified Rankin scale (functional independence), stroke severity on admission (NIHSS), stroke type, applicability for physiotherapy, presence of UTI or pneumonia in first 7 days, comorbidities, stroke onset in hospital (yes/no), hospital's availability of early supported discharge, hospital length of stay, minutes of physiotherapy, intensity of physiotherapy. | Patient records | Quantitative |
| Pilot service evaluation of a brief psychological therapy for self-harm in an emergency department: Hospital Outpatient Psychotherapy Engagement Service. | 2021 | Psychology and psychotherapy | Taylor PJ and Fien K and Mulholland H and Duarte R and Dickson JM and Kullu C | A | M | England | Psychology/ mental health | 89 | Partially- evidence gaps are identified which aim to be fulfilled but does not frame this as an implementation/translation/research to practice issue that a routine data study can address. | Suicide and self-harm are major problems and a substantial volume of people present to emergency departments with self-harm. There is emerging evidence that use of brief psychological therapies in emergency department settings can be beneficial for people presenting with psychological challenges. A new service was set up in an emergency department for people who present with self-harm. | To evaluate the feasibility of a new service delivery model (HOPE), explore patient factors related to engagement, and examine change over time in relation to psychological distress. | Emergency department and outpatient psychology service | Adults presenting to the site's emergency department with self-harm and a history of self-harm. | Age, gender, type of self-harm (including suicidal or non-suicidal), Affective Styles Questionnaire (ASQ) scores, Inventory of Statement about Self-harm (ISAS) scores, Clinical Outcomes in Routine Evaluation (CORE10;) scores, Session Rating Scale (SRS) scores, attendance at sessions. | Patient records | Quantitative |
| Post-traumatic stress disorder (PTSD) treatment experience in Bedford East - audit and reaudit. | 2011 | Psychiatria Danubina | Middleton E and Agius M and Zaman R | A | M | England | Psychology/ mental health | 64 | No | A range of treatments including psychological and pharmaceutical can be used to treat post-traumatic stress disorder (PTSD). | To examine the types of treatments currently used for people with PTSD in the service and compare this with data from 2 years ago to examine any changes. | Community mental health team | Patients referred to the service with a diagnosis of PTSD in the given timeframes | Age, gender, comorbid diagnoses, risk factors present, psychological therapy status, antidepressant treatment, antipsychotic treatment, mood stabilizer treatment, medicines prescribed and dosage. | Patient records | Quantitative |
| Practical challenges and limitations using the Oswestry Disability Low Back Pain Questionnaire in a private practice setting in New Zealand. A clinical audit. | 2012 | New Zealand Journal of Physiotherapy | Nunn, Nicola | C | N/a | New Zealand | Physiotherapy | 447 | Yes- the authors directly discuss evidence generated by controlled trials and the challenges with transferring this to practice and how routinely collect outcome measurement audit presents an alternative. | Routine outcome measures are useful to understand the effects of clinical practice. Outcome measures vary with regards to how much they capture different patient factors. The Oswestry Disability Low Back Pain Questionnaire (ODQ) has been recently implemented in a practice setting. | To examine use of the ODQ in practice and evaluate how this relates to clinical decision making. | Physiotherapy practice | Adult patients accessing the service with an initial diagnosis of low back pain. | Age, gender, number of treatments, days since injury, duration between date of injury and first treatment, ODQ results at initial assessment, discharge and post-discharge follow up. | Patient records | Quantitative |
| Predicting patient engagement in IAPT services: a statistical analysis of electronic health records. | 2020 | Evidence-based mental health | Davis A and Smith T and Talbot J and Eldridge C and Betts D | A | M | England | Psychology/ mental health | 959100 | Partially - the authors acknowledge that there is research focusing on similar aspects but that it is limited due to small sample sizes and argue that the approach using routine data overcomes this. | Lots of factors are thought to influence patient attendance at Improving Access to Psychological Therapies (IAPT) services but the evidence is varied and methodologically limited. | To identify if a generalised linear mixed effects model can be fitted to IAPT data from across the country to predict engagement in therapy, and to predict if a patient will attend their first appointment. | Outpatient psychology | Adults presenting with mental health difficulties referred to the participating IAPT services in the given time period | Age gender, long term condition status, time to first appointment, Agoraphobia-Mobility Inventory scores, Panic Disorder Severity Scale scores, Generalised Anxiety Disorder Assessment scores, Obsessive Compulsive Inventory scores, Impact of Events Scale - Revised scores, Social Phobia Inventory, Statutory sick pay indication, employment status, purpose of appointment, time of appointment, consultation medium (including SMS), Professional role treating, referral source, step intensity, attendance. | Patient records | Quantitative |
| Predictors before and after multimodal rehabilitation for pain acceptance and engagement in activities at a 1-year follow-up for patients with whiplash-associated disorders (WAD)-a study based on the Swedish Quality Registry for Pain Rehabilitation (SQRP). | 2018 | The spine journal : official journal of the North American Spine Society | SÃ¶derlund A and LÃ¶fgren M and StÃ¥lnacke BM | A | M | Sweden | Mixed- pain rehabilitation | 386 | No | A multi-disciplinary approach to rehabilitation of various kinds is often recommended and often applied in pain management. There is little research specifically about the effectiveness of these rehabilitation programmes for people with whiplash associated disorders. | To explore the predictive value of chronic pain acceptance and engagement 1 year following multimodal rehabilitation (MMR) for whiplash associated disorders. | Rehabilitation service in a hospital | Patients with whiplash associated disorder referred into the MMR. | Age, gender, country of birth, education level, work status, expected outcome at start of rehabilitation, Multidimensional Pain Inventory scores, Tampa Scale of Kinesiophobia scores, Chronic Pain Acceptance Questionnaire scores. | Patient records | Quantitative |
| Prehospital Intubation of Patients with Severe Traumatic Brain Injury: A Dutch Nationwide Trauma Registry Analysis. | 2023 | Prehospital Emergency Care | Bossers, Sebastiaan M. and Verheul, Robert and van Zwet, Erik W. and Bloemers, Frank W. and Giannakopoulos, Georgios F. and Loer, Stephan A. and Schwarte, Lothar A. and Schober, Patrick | C | M | Netherlands | Paramedical science | 8946 | Partially- evidence gaps are identified which aim to be fulfilled but does not frame this as an implementation/translation/research to practice issue that a routine data study can address. | The evidence around pre-hospital endotracheal intubation is mixed, and the benefits and risks are not fully understood. Intubation can be performed by paramedics with or without a physician. | To explore the relationship between patient mortality and outcome of those who received endotracheal intubation pre-hospital from either a physician-based emergency service or by paramedics. | Emergency services | Patients with traumatic brain injury who were attended to by the emergency team | Age, gender, Glasgow coma scale scores, injury severity, time to arrival on scene, transport to hospital time, involvement of physician-staffed helicopter, Prehospital intubation status, hospital mortality. | Patient records | Quantitative |
| Prehospital trauma care reduces mortality. Ten-year results from a time-cohort and trauma audit study in Iraq. | 2012 | Scandinavian journal of trauma, resuscitation and emergency medicine | Murad MK and Larsen S and Husum H | M | M | Iraq | Paramedical science | 2778 | Yes- the authors identify the lack of transfer of evidence produced in Wester scenarios to the empirical context and identify the need to create and evaluate new models of working in practice. | "Scoop and run" emergency dispatch services common in Western contexts are not viable in low income and war-torn countries where distances to hospital are far and trauma can be extreme (land mine disasters, bombs, heavy gunfire). More evidence is needed about approaches that are better fit for these contexts. | To evaluate the trauma system that exists in the empirical context | Emergency services | Trauma patients treated by paramedics in the time frame. | Age, gender, Injury severity score, physiological severity score, respiratory rate, blood pressure, site of death (on-site, pre-hospital, in-hospital), prehospital transit time, site of injuries, type of injury (superficial, burn, penetrating, blunt), critical area injuries, (head, neck, torso), expected survivor status. | Patient records | Quantitative |
| Preoperative Counseling in Salvage Total Laryngectomy: Content Analysis of Electronic Medical Records. | 2017 | Otolaryngology--head and neck surgery : official journal of American Academy of Otolaryngology-Head and Neck Surgery | Raol N and Lilley E and Cooper Z and Dowdall J and Morris MA | M | M | USA | Speech and language therapy | 58 | Partially- evidence gaps are identified which aim to be fulfilled but does not frame this as an implementation/translation/research to practice issue that a routine data study can address. | Patients considering salvage total laryngectomy (STL) face difficult decisions on treatment options and must prepare for significant post-surgery impairments. Pre-operative counselling may improve outcomes, but what this involves in poorly standardised, and it is not known what is really said in these sessions. | To explore how often non-surgical and surgical treatment options were discussed with SLT patients, how often patient goals were recorded, and the language involved in these, as well as post-operative discussions and expectations especially around advance care planning. | Tertiary care cancer unit | Patients who underwent total laryngectomy at the hospital in the given time period | Age, gender, mortality, number of notes documented, professional writing the notes, content of medical notes | Patient records | Mixed methods |
| Prevention of falls in hospital: Audit report from a Tertiary care hospital of Pakistan. | 2021 | JPMA. The Journal of the Pakistan Medical Association | Pidani AS and Ahmad T and Panjwani N and Noordin S | C | C | Pakistan | Physiotherapy | 28 | No | Elderly people are at risk of falls and in-hospital falls are common. Falls prevention programmes aim to minimise this. | To evaluate the falls prevention policy in a musculoskeletal service. | Hospital | Patients admitted to hospital under the musculoskeletal service. | Number of falls in hospital, Morse Fall Scale scores. | Patient records | Quantitative |
| Provision of medical and community services to people with severe arthritis; an audit. | 1991 | British journal of rheumatology | Foster HE and Pyle C and Walker DJ | C | C | England | Occupational Therapy | 251 | No | Many people with severe arthritis can become chairbound and non-ambulatory, which is a major cause of disability. The number of people affected in the empirical context is unknown. | To explore how many people referred to the community occupational therapy services experience arthritis which contributes to disability | Community occupational therapy services | Adults with arthritis who had received care from the community occupational therapy service. | Age, gender, Clinical diagnosis, stage of arthritis, mortality, comorbidities, arthritis complications, referral to surgery, surgery execution, ambulatory status, dependence on family. | Patient records plus questionnaire | Quantitative |
| Psychological, social and lifestyle screening of people with low back pain treated by physiotherapists in a National Health Service musculoskeletal service: an audit. | 2023 | European Journal of Physiotherapy | Singh, Gurpreet and McNamee, George and Sharpe, Laura and Lucas, Michael and Lewis, Paul and Newton, Christopher and O'Sullivan, Peter and Lin, Ivan and O'Sullivan, Kieran | C | M | England | Physiotherapy | 81 | Yes- the authors cite relevant evidence but comment that there is an absence of evidence about whether it is translated into routine practice. They conduct the study to investigate implementation. | Evidence suggests that many factors influence the management and outcome of physiotherapy for lower back pain (LBP) including psychological, social and lifestyle factors. Physiotherapists have access to screening tools to support identification of these, which are supported by evidence. There is paucity of research into whether they are used in practice. | To audit the implementation of use of the Short Form Orebro Musculoskeletal Pain Questionnaire (SFÖQ) in routine practice following the setting of a departmental standard | Physiotherapy outpatient service | Patients with non-specific lower back pain referred to the physiotherapy unit. | Age, gender, banding of treating physiotherapist, number of physiotherapy treatments, SFOQ factors assessed, completion of SFOQ. | Patient records | Quantitative |
| Quality of English inpatient mental health services for people with anxiety or depressive disorders: Findings and recommendations from the core audit of the National Clinical Audit of Anxiety and Depression. | 2021 | Comprehensive psychiatry | Baldwin DS and Dang M and Farquharson L and Fitzpatrick N and Lindsay N and Quirk A and Rhodes E and Shah P and Williams R and Crawford MJ | A | M | England | Psychology/ mental health | 3795 | No | Psychological care in England is varied and some patients report negative experiences. Audit efforts and quality improvement programmes have tried to address this. Quality standards exist to guide service providers. The National Clinical Audit of Anxiety and Depression evaluates quality of care across services. | To develop quality of mental health services and evaluate comparative data from across sites. | Hospital | Adults with mental health difficulties admitted to hospital | Age, gender, ethnicity, employment status, accommodation status, main diagnoses, additional diagnoses, adherence to standards according to medical notes relating to admission, assessment, care plan, medication, psychological therapies, physical health (BMI, blood pressure, glucose, cholesterol, smoking/alcohol/substance consumption), discharge, readmission, follow-up data, crisis planning and use of outcome measures. | Patient records | Quantitative |
| Real world evidence of improved attention and cognition during physical therapy paired with neuromodulation: a brain vital signs study. | 2023 | Frontiers in human neuroscience | Kirby ED and Jones CB and Fickling SD and Pawlowski G and Brodie SM and Boyd LA and Venter J and Moser N and Kalsi-Ryan S and Medvedev G and D'Arcy RCN | A | M | Canada | Physiotherapy | 33 | Yes - the authors outline the existing research and identify that understanding the intervention in the 'real world' is the next step, which provides the context of the study | There is good evidence emerging that supports the use of combining physiotherapy with Translingual neurostimulation (TLNS) in trials to improve outcomes of people with neurological disorders. Little is known about the real-world application of this. | To evaluate whether combined TLNS + physiotherapy treatment created changes in brain vital signs in a diverse neurorehabilitation group. | Rehabilitation clinic | Adults with neurological conditions referred to the participating clinics | Age, gender, neurological condition, brain vital sign assessment (baseline and post-rehab), cognitive status (CogBAT). | Patient records | Quantitative |
| Record audit: a study of the quality and effectiveness of the treatment of knee conditions. | 1995 | Physiotherapy | C, Dobson | C | N/a | England | Physiotherapy | 187 | No | Clinical audit is considered to provide incentive for quality improvement. | To utilise audit methods to evaluate physiotherapy service quality for people with knee conditions | Physiotherapy outpatient service | Adults referred to the department with diagnoses knee condition | Age, date of referral to physiothearpy, diagnosis, Physiotherapy Inputs (PIU metric based on minutes of therapy and grade of staff providing therapy), clinical outcome measures, discharge status, attendance at appointments, wait time between referral and first appointment. | Patient records | Quantitative |
| Redesigning care for back pain in an Australian hospital setting: A service evaluation to identify need for change. | 2023 | Musculoskeletal Care | Gorgon, Edward and Maka, Katherine and Sullivan, Justin and Nisbet, Gillian and Hancock, Michelle and Regan, Gerard and Leaver, Andrew | A | M | Australia | Physiotherapy | 252 | Yes- the authors comment on the best practice guidelines and address the challenges in implementing them in the real world, and the study is situated in this context. | Back pain is a major problem in Australia with a large healthcare burden, though the effectiveness of care is unclear. Recent guidelines indicate a more holistic approach to care is valuable but fall short of guiding how this can be implemented in practice. Hospitals may be designed to address back pain though health service redesign is required. | To evaluate processes of care for patients with back pain in physiotherapy clinics to inform a future service redesign. | Physiotherapy outpatient service | Patients referred to physiotherapy with back pain in the given period. | Age, gender, country of birth, primary language, need for interpreter, emoployment status, referral source, wait time from referal to first appointment, back pain features (chronicity, area affected, body location of pain, surgery status), length of episode of care, individual or group sessions given, attendance at appointments, discharge status/destination, return to physiotherapy | Patient records | Quantitative |
| Reducing unnecessary skull radiographs in children: a multidisciplinary audit. | 2004 | Clinical radiology | Johnson K and Williams SC and Balogun M and Dhillon MS | C | N/a | England | Radiography/Radiotherapy | 1704 | No | Skull radiography of children with head trauma is a point of contention in research as it may identify an underlying brain injury but does not exclude significant intra-cranial injuries. Radiation exposure should be limited. New practice guidance was produced to guide skull radiograph requests. | To audit the use of the guidelines in the local site across 3 audit cycles and investigate compliance of skull radiograph requests | Radiography department in a hospital | Children attending hospital with head trauma | Clinical indicators for skill radiograph, skill radiography performed, abnormality found, CT performed, compliance with guidelines. | Patient records | Quantitative |
| Reduction in hospital time to thrombolytic therapy by audit of policy guidelines. | 1990 | European heart journal | MacCallum AG and Stafford PJ and Jones C and Vincent R and Perez-Avila C and Chamberlain DA | C | C | England | Paramedical science | 50 | No | The time between onset of myocardial infarction and thrombolytic therapy is important in influencing outcomes. Rapid responses from emergency services and a minimal delay to getting to hospital is beneficial. | To evaluate arrival times for patients to hospital and initiation of thrombolytic therapy for patients identified by paramedics as candidates for 'Fast Track'. | Emergency services | Adults presenting with myocardial infarction attended to by paramedics in the given site. | Age, diagnosis, Adherence to 'fast track' criteria, Contraindications for thrombolytic therapy, Receipt of thrombolytic therapy, type of thrombolytic treatment, ‘door to needle time', reasons for delay. | Patient records | Quantitative |
| Referral patterns to primary mental health services in Western Sydney (Australia): an analysis of routinely collected data (2005-2018). | 2020 | International journal of mental health systems | Munasinghe S and Page A and Mannan H and Ferdousi S and Peek B | M | M | Australia | Psychology/ mental health | 20507 | Partially - the authors acknowledge research and policy about best practice in mental health services but highlight that more needs to be known about the local, 'real' context to implement. | Australia has a high proportion of people with mental and substance use disorders. The government have established a programme to enhance access to mental health services that are low cost or free in primary mental health care (PMHC). To be effective, local contexts need to be understood for service design to be appropriate. | To examine referral pathways and utilisation of PMHC in the local context. | Primary care - mental health service | Adults referred to PMHC in the given period and given location. | Age, gender, socio-economic status, diagnosis, Referral source, Principal focus of treatment, Suicide referral status, Non-attendance to treatment, Waiting time for first appointment, Mode of contact, Number of sessions, date of referral. | Patient records | Quantitative |
| Relationship between emotional words in electronic medical records and leave periods of users of a return-to-work program with depression. | 2022 | British Journal of Occupational Therapy | Kutsuna, Ichiro and Hoshino, Aiko and Morisugi, Ami and Mori, Yukari and Shirato, Aki and Takeda, Mirai and isaji, Hikari and Suwa, Mami | M | M | Japan | Occupational therapy | 42 | No | There is a need to support people to return to work (RTW) which is supported by occupational therapists. Evaluation about RTW can be subjective, does not always consider variation in emotional state and with few objective measures. Natural language processing may present one option for evaluation that is less subjective. | To assess and score emotional states of people undergoing RTW programmes using natural language processing using medical records and evaluate the association of this with duration of sick leave. | Mental health clinic | Adults with a diagnosis of depression or adjusted disorder and who were on sick leave from work accessing the clinic. | Age, gender, diagnosis, somatic disease, education level, living situation, length of employment, employing organisation, occupation, position, number of sick leave times taken, depression severity (Beck Depression Inventory-II) scores, duration of program participation, textual data from electronic medical records. | Patient records | Mixed methods |
| Retrospective audit of the acute management of stroke in two district general hospitals in the uk. | 2008 | Annals of Ibadan postgraduate medicine | Faluyi OO and Omodara JA and Tay KH and Muhiddin K | C | C | England | Mixed- stroke rehabilitation | 98 | No | Major determinants of outcomes post-stroke are the treatment given in the acute stages and secondary prevention measures. There are clinical practice guidelines on this management. | To evaluate whether local hospitals conformed to the guidelines about stroke management. | Hospitals | Adults with suspected stroke admitted to the selected sites | Age, gender, diagnosis (stroke or TIA), stroke type, Conformity of care with guidelines (standards for Acute management, secondary prevention, multi-disciplinary care) | Patient records | Quantitative |
| Retrospective study of first episode psychosis in the Dublin Southwest Mental Health Service: demographics, clinical profile and service evaluation of treatment. | 2019 | Irish journal of psychological medicine | Clarke AM and McLaughlin P and Staunton J and Kerins K and Power B and Kearney K and McGuinness M and Whitty P | C | C | Ireland | Psychology/ mental health | 66 | No | A new healthcare initiative on treatment of first episode of psychosis is being rolled out in response to evidence about the need in the population. To evaluate the effectiveness of the new initiative, there is a need to understand baseline context of patients and treatments. | To explore the characteristics of people currently accessing services, what their treatment programmes were, and their outcomes from it. | Community mental health service | Adults with first episode psychosis presenting to the team. | Age, gender, relationship status, living arrangements, employment, educational level, diagnosis, substance use, psychiatric history, re-admission over subsequent 2 years, intervention received, physical health metrics (weight, BMI etc.), antipsychotic prescriptions given. | Patient records | Quantitative |
| Risk Adjustment of the Modified Low Back Pain Disability Questionnaire and Neck Disability Index to Benchmark Physical Therapist Performance: Analysis From an Outcomes Registry. | 2020 | Physical therapy | Lutz AD and Brooks JM and Chapman CG and Shanley E and Stout CE and Thigpen CA | M | M | USA | Physiotherapy | 182276 | No | Many patient characteristics can influence treatment outcome in musculoskeletal health, but the therapist performance/quality of care may also play a role. This is currently under researched. | To develop a model for back and neck pain treatment that compares physiotherapist performance. | Physiotherapy services | Adults with back or neck pain accessing physiotherapy from the provider (and in the registry) | Age, gender, Modified Low Back Pain Disability Questionnaire (MDQ) scores, Neck Disability Index (NDI) scores, Veterans RAND 12-Item Health Survey scores, Household income, Insurance type, duration of symptoms, BMI, comorbidities, Treating physiotherapist. | Patient records | Quantitative |
| Sensory Symptoms and Signs of Hyperarousal in Individuals with Fragile X Syndrome: Findings from the FORWARD Registry and Database Multisite Study. | 2023 | Journal of autism and developmental disorders | Lachiewicz AM and Stackhouse TM and Burgess K and Burgess D and Andrews HF and Choo TH and Kaufmann WE and Kidd SA | C | M | USA | Mixed - neurodevelopmental disorders | 933 | Partially- evidence gaps are identified which aim to be fulfilled but does not frame this as an implementation/translation/research to practice issue that a routine data study can address. | Fragile X syndrome is associated with sensory symptoms (SS) and hyperarousal (HA) although there is relatively less empirical research about them. Not much is known about how these symptoms relate to someone's participation in activities. | To examine the prevalence of SS and HA in individuals with Fragile X syndrome, including co-occurrence rates and with other symptoms, how it is associated with participation and how it is managed. | Outpatient clinic | Individuals with Fragile X syndrome attending the selected clinics. | Age, gender, race and ethnicity, parent report form, clinical report form, Aberrant Behavior Checklist-Community (ABC-C) scores, IQ scores or intellectual functioning assessment, presence of autism spectrum disorder, Answers to registry questions pertaining to SS and HA as well as behaviour, medication, interventions, and therapies. | Patient records via Registry | Quantitative |
| Service evaluation of a Human Givens Therapy service for veterans. | 2019 | Occupational medicine (Oxford, England) | Burdett H and Greenberg N | A | A | England | Psychology/ mental health | 504 | No | Veterans are at high risk of having Post traumatic stress disorder (PTSD), and mental health support is often through charitable services. Human Givens Therapy (HGV) is a relatively new intervention approach without a strong evidence base. | To examine the outcomes from veterans accessing HGV therapy and how patient factors were associated with this and compare this with outcomes and attrition rates of NHS IAPT services. | Charity | Veterans with PTSD accessing the charitable service | Age, gender, employment status, accommodation status, relationship status, dependents, self-reported distress scales scores (baseline and post-therapy), prior treatment received, pharmaceutical use, 10-item Clinical Outcomes in Routine Evaluation (CORE-10) scores (baseline and post-therapy) | Patient records | Quantitative |
| Service evaluation of telehealth in a physiotherapy musculoskeletal setting: Patient outcomes and results from risk stratification. | 2022 | Musculoskeletal care | Slattery B and Ackerman L and Jagadamma KC | C | A | Scotland | Physiotherapy | 89 | No | COVID-19 accelerated the use of telehealth in the NHS. There is good evidence to support use of telehealth in musculoskeletal physiotherapy. Local services have transferred to using telehealth and blended approaches to therapy. | To evaluate whether patients accessing the service using a blended approach improve their outcomes, and to examine whether a new screening tool can identify patients at risk of poorer outcomes using telehealth. | Physiotherapy outpatients | Adults with musculoskeletal complaints referred into the service. | Age, gender, MSK condition, employment status, stage of condition (acute, sub-acute, chronic), risk stratification tool outcome tool (including Numerical Pain Rating Scale) pre and post therapy, medium used (phone, face to face, telehealth, or a blend), attendance at appointment/programme completion, Musculoskeletal Health Questionnaire (MSK HQ) scores pre and post therapy. | Patient records | Quantitative |
| Sheffield spinal pathway audit cycle - pathways, mountains and the view from the top. | 2014 | British journal of pain | Hart O and Ryton BA | C | C | England | Mixed- pain rehabilitation | 1325 | Partially- evidence gaps are identified which aim to be fulfilled but does not frame this as an implementation/translation/research to practice issue that a routine data study can address. | In the empirical context, GPs refer patients to one of two pathways where those with 'red flags' are directly referred to secondary care (urgent referrals) and others referred to spinal specialist teams for assessment and physiotherapy (routine referrals). It was noted that the urgent referrals to secondary care were often inappropriate. | To examine the adherence to the pathway guidance. | GP practice | Adults with spinal-related pain presenting to participating GP sites. | Referral type (urgent, routine), pain type, nerve root pain, symptom timescale, surgical input required, psychosocial assessment scores, referral destination (orthopaedics, neurosurgery, CarePlus, Physioworks), year of referral. | Patient records | Quantitative |
| Silence in the EHR: infrequent documentation of aphonia in the electronic health record. | 2014 | BMC health services research | Morris MA and Kho AN | A | A | USA | Speech and language therapy | 81 | No | It is important that healthcare staff know if someone they are supporting has a communication disability. Recording this in medical documentation is helpful. There is limited understanding of how providers do this in practice, and how the nuances of changes in disability level are captured. People with laryngectomy often have some kind of communication disability. | To examine the extent of documentation of communication dis/abilities in patient records. | Hospital | Adults who had undergone a laryngectomy at the selected site. | Age, gender, ethnicity, laryngectomy type (CPT code), ICD-9 codes related to communication, textual information from notes about communication ability | Patient records | Mixed methods |
| Specialist adult ADHD clinics in East Anglia: service evaluation and audit of NICE guideline compliance. | 2015 | BJPsych bulletin | Magon RK and Latheesh B and MÃ¼ller U | C | M | England | Mixed - neurodevelopmental disorders | 150 | Partially- evidence gaps are identified which aim to be fulfilled but does not frame this as an implementation/translation/research to practice issue that a routine data study can address. | ADHD is a very common neurodevelopmental disorder and new practice guidelines have been developed which have been implemented in the given site. | To evaluate compliance with guidelines for ADHD assessment and management in the given clinics. | ADHD Clinic | Adults referred to the clinics in the given timeframe. | Age, gender, ethnicity, employment status, clinic attending, transition to adult services from child services, adherence to guidance (in relation to: diagnosis and treatment, psychological intervention, alcohol and substance misuse, person-centred care, organisational service). | Patient records | Quantitative |
| Staffing levels and hospital mortality in England: a national panel study using routinely collected data. | 2023 | BMJ open | Rubbo B and Saville C and Dall'Ora C and Turner L and Jones J and Ball J and Culliford D and Griffiths P | A | A | England | Mixed- acute hospital services | 540 | Partially- evidence gaps are identified which aim to be fulfilled but does not frame this as an implementation/translation/research to practice issue that a routine data study can address. | Hospital staffing is an important consideration for safe and efficient hospitals. Much research into staffing levels focuses on nurses and little is known about allied health professions. | To examine the association between allied health professionals' staffing levels and patient mortality in hospitals. | Acute adult inpatient services | AHP staff employed in the participating acute inpatient sites and adult patients admitted to acute inpatient sites. | Patient mortality, staffing levels of each profession. | Patient records via registry, plus organisational data | Quantitative |
| Systematic services audit of consecutive suicides in New Brunswick: the case for coordinating specialist mental health and addiction services. | 2008 | Canadian journal of psychiatry. Revue canadienne de psychiatrie | Lesage A and SÃ©guin M and Guy A and Daigle F and Bayle MN and Chawky N and Tremblay N and Turecki G | A | M | Canada | Psychology/ mental health | 102 | No | Many people who die by suicide have not been committed to mental health services. Those who have often do not have systematic data collected about them. Inadequate recommendations for strategies may be inappropriate if they are based on this absence information. Psychological autopsy can be used to gather information. This can be used to help understand what the unmet needs are and potential shortcomings in intervention and prevention programmes. | To establish priorities for suicide prevention strategies through examining patient history and past service access. | Mental health services (hospital and community) | People who died by suicide in the given time frame and area | Gender, age, ethnicity, marital status at time of death, employment status at time of death, disability status at time of death, mental health disorder diagnoses, symptoms, comorbidity, interventions provided, service use. | Patient records plus coroner reports | Quantitative |
| Ten-year audit of clients presenting to a specialised service for young people experiencing or at increased risk for psychosis. | 2014 | BMC psychiatry | Conrad AM and Lewin TJ and Sly KA and Schall U and Halpin SA and Hunter M and Carr VJ | M | M | Australia | Psychology/ mental health | 1997 | Yes- the authors specifically describe issues of implementation of research into practice and their study is situated in this context. | There is much research around psychosis risk and early intervention though it is conflicting. Comorbidities and the nature and severity of presenting problems may influence outcomes. Specialised psychosis services could offer insight to the debate. | To evaluate a specialist psychosis service with respect to the clinical characteristics of people presenting and the outcomes of those who engaged in treatment compared to those who did not. | Community specialist mental health service | Young people with a recent episode of psychosis accessing the service. | Age, gender, referral source, marital status, educational level, accommodation status, employment status, diagnosis, psychosis status (existing or recent, ultra-high risk), comorbidities, number of contacts with services (community, admissions), admission details (number of admissions, number of bed days), receipt of ongoing treatment at specialist service. | Patient records | Quantitative |
| The assessment of pain: an audit of physiotherapy practice. | 1996 | The Australian journal of physiotherapy | Turner P and Whitfield A and Brewster S and Halligan M and Kennedy J | A | M | England | Physiotherapy | 1010 | Partially- evidence gaps are identified which aim to be fulfilled but does not frame this as an implementation/translation/research to practice issue that a routine data study can address. | Objective measurement of pain is useful to evaluate effectiveness of physiotherapy intervention. The extent to which pain measurement is routinely quantified in clinical practice is uncertain. | To evaluate the extent to which the symptom of pain was recorded and measured by physiotherapists, and how. | Physiotherapy department of a hospital (in and outpatients) | Adults with a complaint of pain presenting to the service. | Age, gender, in/outpatient and type, pain assessment undertaken (baseline and repeat), how assessment was taken (quantified or descriptive), treating physiotherapist expertise, pain treatment given. | Patient records | Quantitative |
| The birth trauma psychological therapy service: An audit of outcomes. | 2021 | Midwifery | Williamson E and Pipeva A and Brodrick A and Saradjian A and Slade P | C | M | England | Psychology/ mental health | 114 | Yes- the authors highlight translation of evidence to practice challenges and offer how practice-based evidence (such as routinely collected data studies) can support this. | Post-traumatic stress disorder (PTSD) post-childbirth is increasingly common. Interventions for PTSD do receive a lot of scholarly attention but not much is known about outcomes for PTSD following childbirth. | To evaluate the impact of a psychological therapy service for women attending a 'birth trauma clinic' who have PTSD. | Outpatient clinic | Women with PTSD following childbirth attending the clinic | Age, number of children, months post-natal, Impact of Event Scale-Revised (IES-R) scores, Patient Health Questionnaire (PHQ-9) scores, Generalised Anxiety Disorder (GAD-7) scores, CORE-10 scores, assessment only or therapy commenced, therapy completion. | Patient records | Quantitative |
| The Cardiac Arrest Support Tier: a service evaluation. | 2020 | British paramedic journal | Metcalf M and Robinson M and Hall P and Goss J | C | M | England | Paramedical science | 178 | No | The survival rate of people who have a cardiac arrest out of hospital is low in the UK and the empirical context. An approach was piloted which aimed to tackle this - the Cardiac Arrest Support Tier’s (CAST) which involved paramedics who are special trained to deliver clinical care to patients in hazardous environments delivering a designated cardiac arrest team (CAST). | To evaluate the new model in terms of its clinical effectiveness, feasibility, and acceptability. | Emergency services | Adults who had experienced an out of hospital cardiac arrest | Age, gender, bystander CPR given, cardiac rhythm (shockable or non-shockable), cause (traumatic or medical), Treating team (CAST or other), Treating paramedic history (exposure to cardiac arrest in previous year), aspects of care bundle given, Survival of patient. | Patient records | Quantitative |
| The Clinical and Economic Burden of Tardive Dyskinesia in Israel: Real-World Data Analysis. | 2022 | Journal of clinical psychopharmacology | Barer Y and Ribalov R and Yaari A and Maor R and Arow Q and Logan J and Chodick G and Arkadir D and Eitan R | A | M | Israel | Mixed- movement disorder | 454 | No | Tardive dyskinesia (TD) is a complex disorder with substantial healthcare resource requirements. It can be caused by antipsychotic medications. Little is known about the health care burden of TD. | To evaluate patient characteristics, treatment, and economic burden of TD in Israel. | Mixed | Adults with diagnosis of TD accessing participating health care services | Age, gender, diagnosis, age of TD diagnosis, socioeconomic status, healthcare access prior to diagnosis, duration of healthcare to follow-up, comorbidities, antipsychotic medication use, type of antipsychotic medication, duration of antipsychotic use, professional diagnosing TD, continuation rate of medicine. | Patient records | Quantitative |
| The comparative effectiveness and efficiency of cognitive behaviour therapy and generic counselling in the treatment of depression: evidence from the 2(nd) UK National Audit of psychological therapies. | 2017 | BMC psychiatry | Pybis J and Saxon D and Hill A and Barkham M | PS | M | England | Psychology/ mental health | 33243 | Partially- evidence gaps are identified which aim to be fulfilled but does not frame this as an implementation/translation/research to practice issue that a routine data study can address. | Most people with depression are treated with CBT in the UK according to the model or care, though counselling is offered if this does not lead to improved outcomes. Counselling is widely used, but there is little evidence of its effectiveness in the literature. | To examine the effect of CBT and counselling offered through the Improving Access to Psychological Therapists (IAPT) service on patient outcomes. | Community mental health service | Adults with depression accessing any treatment via IAPT in the given timeframe. | Age, gender, ethnicity, diagnosis or diagnoses, therapy accessed (CBT or counselling), therapy completion, clinical outcomes (Patient Health Questionnaire-9 (PHQ-9) and the Generalised Anxiety Disorder (GAD-7)). | Patient records | Quantitative |
| The Cost-Effectiveness of the Improving Access to Psychological Therapies (IAPT) Programme in Severe Mental Illness: A Decision Analytical Model Using Routine Data. | 2019 | Community mental health journal | Zala D and Brabban A and Stirzaker A and Kartha MR and McCrone P | A | M | England | Psychology/ mental health | Not reported | Yes- the authors comment on current evidence from controlled trials but argue how understanding real world clinical practice and real-world populations to study is useful. | People with severe mental illness (SMI) have heavy healthcare utilisation. Economic evaluations of treatment for SMI have mixed results. The Improving Access to Psychological Therapies (IAPT) service in the UK piloted a service for people with SMI. | To evaluate IAPT treatments for people with SMI to assess their cost effectiveness. | Community mental health service | Adults with SMI presenting to participating sites | Age, gender, SMI diagnosis, type of treatment given, functional impairment scores (pre and post therapy), incidence of harmful behaviours, treatment attrition (completer or drop out). | Patient records | Quantitative |
| The effectiveness and cost of corticosteroid injection and physiotherapy in the treatment of frozen shoulder-a single-centre service evaluation. | 2014 | Clinical rheumatology | Bateman M and McClymont S and Hinchliffe SR | C | C | England | Physiotherapy | 55 | Yes- the authors comment on existing evidence but critique its transferability to clinical practice in the UK. | Frozen shoulder is a very common physiotherapy complaint. This is often treated with eclectic physiotherapy or if there is no improvement, corticosteroid injections. The effectiveness and cost effectiveness of injections is unknown. | To evaluate current clinical practice for frozen shoulder, explore the effectiveness of physiotherapy and corticosteroid injections and their associated costs. | Physiotherapy outpatient service | Adults with frozen shoulder attending the site | Age, gender, diabetes diagnosis, thyroid disorder diagnosis, duration of symptoms, treatment given (injection, type of physiotherapy, number of contacts, discharge status, patient-reported pain scores (pre and post therapy). | Patient records | Quantitative |
| The effectiveness of high-intensity CBT and counselling alone and following low-intensity CBT: a reanalysis of the 2nd UK National Audit of Psychological Therapies data. | 2018 | BMC psychiatry | Barkham M and Saxon D | A | A | England | Psychology/ mental health | 33,243 | No | Adults with depression are referred to Improving Access to Psychological Therapies (IAPT) initiative to receive treatment in the UK and receive either CBT or counselling services, which may be low or high intensity per the 'Step' that the care is given, ie. Low intensity CBT/High intensity CBT, High intensity CBT, Low intensity CBT/High intensity Counselling, and High intensity Counselling | To compare CBT and counselling across the different steps and intensity. | Community mental health service | Adults with depression accessing any treatment via IAPT in the given timeframe. | Type of therapy, intensity of therapy, Patient Health Questionnaire- 3and Generalised Anxiety Disorder-7 scores before, throughout and after treatment, number of sessions, | Patient records | Quantitative |
| The effectiveness of ultrasound guided hydrodistension and physiotherapy in the treatment of frozen shoulder/adhesive capsulitis in primary care: a single centre service evaluation. | 2017 | Shoulder & elbow | Bryant M and Gough A and Selfe J and Richards J and Burgess E | C | M | England | Physiotherapy | 33 | No | Frozen shoulder is a very common physiotherapy complaint. Hydrodistension intervention may be one approach, but the evidence is unclear about how it compares to other approaches (such as physiotherapy and corticosteroid injection). Hydrodistension can be performed by radiology or a sonography-trained physiotherapist. | To evaluate the effectiveness of physiotherapist-led hydrodistension therapy plus physiotherapy for patients with frozen shoulder. | Physiotherapy outpatient service | Adults with frozen shoulder diagnosis who received hydrodistension and a physiotherapy programme | Age, gender, diabetes diagnosis, duration of symptoms, previous injections received, previous physiotherapy received, pre and post therapy clinical outcome measures (Patient reported outcome measures, Shortened Disabilities of the Arm, Shoulder and Hand (QuickDASH) score and Shoulder Pain Disability Index (SPADI) and Clinical Recorded Outcome Measures (CROMs) on pain and should movement). | Patient records | Quantitative |
| The extended scope physiotherapist in orthopaedic out-patients - an audit. | 2006 | Annals of the Royal College of Surgeons of England | Pearse EO and Maclean A and Ricketts DM | C | C | England | Physiotherapy | 150 | No | Extended scope physiotherapists (ESPs) can support orthopaedic clinics demands by assessment and treating many cases. | To audit ESPs in an orthopaedic clinic and assess adherence to standards. | Orthopaedic clinic (secondary care) | Adults referred to orthopaedic clinic for physical complaints | Age, gender, site of pathology, independence of ESP in care (assessment and management),independent physiotherapy management (advice, physiotherapy programme), requirement for consultant review, outcome following consultant review, re-referral rate | Patient records plus questionnaire | Quantitative |
| The French Cochlear Implant Registry (EPIIC): Perception and language results in infants with cochlear implantation under the age of 24 months. | 2020 | European annals of otorhinolaryngology, head and neck diseases | Loundon N and Simon F and Aubry K and Bordure P and Bozorg-Grayeli A and Deguine O and Eyermann C and Franco-Vidal V and Godey B and Guevara N and Karkas A and Klopp N and Labrousse M and Lebreton JP and Lerosey Y and Lescanne E and Marianowski R and Merklen F and Mezouaghi K and Mom T and Moreau S and Mosnier I and NoÃ«l-Petroff N and Parietti-Winkler C and Piller P and Poncet C and Radafy E and Roman S and Roux-Vaillard S and Schmerber S and Tavernier L and Truy E and Vincent C and De Lamaze A | C | M | France | Speech and language therapy | 615 | No | In 2011, France launched the French Registry of cochlear implants (EPIIC) registry to collect data about people with cochlear implants. It was the only existing national registry at the time of publication. | To examine the information available for children who were operated on before they were 2 years or age. | Mixed - data is from a range of settings | Children who were born deaf and who had surgery for a cochlear implant before the age of two | Cause of deafness, Age of first cochlear implant, surgeries received (first implantation, explanation, re-implantations), bilateral or unilateral, speech therapy data over time (auditory perception, language development, speech intelligibility, comprehension), type of schooling, | Patient records via Registry | Quantitative |
| The impact of introducing hydrodistension as a treatment fo frozen shoulder in a primary care musculoskeletal service: A retrospective audit. | 2023 | Musculoskeletal Care | Whelan, Gareth and Yeowell, Gillian and Littlewood, Chris | C | M | England | Physiotherapy | 102 | No | People with Frozen shoulder often access primary care to begin treatment. However, the treatment is typically at a secondary care (hospital) setting. Specially trained physiotherapists can provide hydrodistension treatment at the point of care. | To evaluate onward referral rates made by treating physiotherapists providing hydrodistension and compare this to historical data. | Primary care musculoskeletal clinic | Adults with frozen shoulder accessing the primary care site in the given timeframe (plus historical data) | Age, gender, diabetes diagnosis, months from referral to treatment, injection procedure details (type, professional providing treatment, position, guidance method (ultrasound or palpation), corticosteroid used, anaesthetic used, saline used, volume of injection, resolve of symptoms, onward referral. | Patient records | Quantitative |
| The impact of seven-day working for patients and staff in an acute physical older adults unit: A service evaluation. | 2017 | British Journal of Occupational Therapy | Davies, Naomi and Simelane, Brian | C | C | England | Occupational therapy | 1851 | No | The professional body and government in the UK recommend occupational therapy should be available in acute services seven days a week. There is little research examining the impact of such services. | To evaluate the clinical outcomes from adults in the acute services in a newly expanded seven-day occupational therapy service. | In patient acute services | Adults admitted to the acute care ward | Date of admission to ward, discharge date and day of the week, length and time of occupational therapy sessions. | Patient records plus questionnaire | Quantitative |
| The impact of the frequency, duration and type of physiotherapy on discharge after hip fracture surgery: a secondary analysis of UK national linked audit data. | 2022 | Osteoporosis international : a journal established as result of cooperation between the European Foundation for Osteoporosis and the National Osteoporosis Foundation of the USA | Goubar A and Ayis S and Beaupre L and Cameron ID and Milton-Cole R and Gregson CL and Johansen A and Kristensen MT and Magaziner J and Martin FC and Sackley C and Sadler E and Smith TO and Sobolev B and Sheehan KJ | A | M | UK | Physiotherapy | 5395 | Yes - the authors identify a gap in research and critique that little is known about what 'usual care' is, which situates their study auditing what routine clinical practice looks like. | The evidence is mixed about what best practice is for physiotherapy following hip surgery, and how physiotherapy influences outcomes. | To describe the type (frequency, duration, approach) of physiotherapy commonly given post- hip surgery and to examine if there was an association between these characteristics and discharge. | Acute hospital | Adults aged 60 years or over with hip fracture who had surgery | Age, gender, ethnicity, social-economic deprivation, Charlson Comorbidity Index, American Society of Anaesthesiologists (ASA) grade, pre-fracture ambulation, type of fracture, surgery given within target timeframe, type of procedure, day of admission (Mon-Fri or weekend), first mobilisation after surgery target met, pre-fracture accommodation type, anaesthesia type used, Hospital Frailty Index score, duration of physiotherapy given, type of physiotherapy given. | Patient records | Quantitative |
| The Influence of Patient Choice of First Provider on Costs and Outcomes: Analysis From a Physical Therapy Patient Registry. | 2018 | The Journal of orthopaedic and sports physical therapy | Denninger TR and Cook CE and Chapman CG and McHenry T and Thigpen CA | C | M | USA | Physiotherapy | 603 | Partially - the authors comment on what best practice states, and what happens in practice and articulate a mismatch. The study aims to deliver new information to address this gap. | Physiotherapy may support spinal-pain patients' outcomes, although best practice advises as 'watchful waiting' approach and physiotherapy is only referred to if not improvement is observed. More evidence is needed about direct access to physiotherapy. | To compare direct physiotherapy access and traditional referral programmes in terms of clinical outcomes and cost effectiveness. | Physiotherapy services | Adults with low back or neck related complaints | Age, gender, primary diagnosis, secondary diagnosis, duration of symptoms, Clinical measures, and post treatment (Numeric pain-rating scale [NPRS]), disability (ODI or NDI), psychosocial features (Patient Health Questionnaire [PHQ-4]), and overall health status (European Quality of Life-5 Dimensions [EQ-5D]), treatment arm (direct physio or traditional). | Patient records | Quantitative |
| The Japanese registry for surgery of ischial pressure ulcers: STANDARDS-I. | 2018 | Journal of wound care | Yanagi H and Terashi H and Takahashi Y and Okabe K and Tanaka K and Kimura C and Ohura N and Goto T and Hashimoto I and Noguchi M and Sasayama J and Shimada K and Sugai A and Tanba M and Nakayama T and Tsuboi R and Sugama J and Sanada H | M | M | Japan | Mixed- physiotherapy and occupational therapy | 59 | No | Treatment for pressure ulcers includes a multi-disciplinary team including physiotherapists. Having standards for indication for surgical treatment is helpful. | To establish surgical indicators for patients with pressure ulcers | Mixed | Adults with pressure ulcers | Age, gender, height and weight, primary illness, comorbidities, availability of therapists, functional independence measures, surgical history, scoliosis status, wheelchair adjustments, infection status, blood test measures, location, and size of wound. | Patient records | Quantitative |
| The national clinical audit of falls and bone health-secondary prevention of falls and fractures: a physiotherapy perspective. | 2010 | Physiotherapy | Goodwin V and Martin FC and Husk J and Lowe D and Grant R and Potter J | A | M | England | Physiotherapy | 8826 | Partially- evidence gaps are identified which aim to be fulfilled but does not frame this as an implementation/translation/research to practice issue that a routine data study can address. | Hip fractures in older people are common following falls. The evidence is mixed regarding the effectiveness of physiotherapy for people who have had falls and how to identify those at most risk of subsequent falls. More evidence is needed about new ways to manage falls and frailty services. | To evaluate how patients were managed pre-and post-hip fracture surgery and the activities around secondary prevention in relation to physiotherapy services and best practice guidelines. | Acute hospital | Patients over the age of 65 who experienced a fracture following a fall. | Type of fracture (Hip or non-hip), Hospital admission, Physiotherapy-related assessment conducted (gait, balance, mobility), functional ability status measured, neuromuscular status measured, fear of falling questioned, attendance to exercise programme, attendance to falls prevention programme, exercise prescription by trained professional, training delivered on how to get up from floor, written advice given, attendance at falls clinic. | Patient records | Quantitative |
| The prevalence of foot pain and association with baseline characteristics in people participating in education and supervised exercise for knee or hip osteoarthritis: a cross-sectional study of 26,003 participants from the GLA:DÂ® registry. | 2023 | Journal of foot and ankle research | Gates LS and Cherry L and GrÃ¸nne DT and Roos EM and Skou ST | A | M | England but data is from Denmark. | Physiotherapy | 26003 | Partially- evidence gaps are identified which aim to be fulfilled but does not frame this as an implementation/translation/research to practice issue that a routine data study can address. | Foot pain may exacerbate pain from knee osteoarthritis or may increase the risk of developing knee symptoms. More research about the relationship between foot and knee symptoms is needed. | To explore the prevalence of foot pain in people who have knee or hip osteoarthritis, examine clinical characteristics, and see if they are associated. | Physiotherapy outpatients | Adults with knee or hip symptoms and/or hip osteoarthritis receiving healthcare services | Age, gender, BMI, most affected joint, pain medication, presence of depression, physical activity level, foot pain presence, distribution of foot pain, most affected joint side, knee/hip severity, knee pain distribution, hip pain distribution, number of knee/hip joints affected, | Patient records via Registry | Quantitative |
| The role of depression in the association between physiotherapy frequency and duration and outcomes after hip fracture surgery: secondary analysis of the physiotherapy hip fracture sprint audit. | 2023 | European geriatric medicine | Milton-Cole R and O'Connell MDL and Sheehan KJ and Ayis S | A | A | England | Mixed- physiotherapy and psychology / mental health services | 5005 | No | Patients who have had hip fracture surgery and depression may experience greater challenges with recovery than those without depression. Patients who have had surgery require physiotherapy. Depression may lead to variation in engagement with physiotherapy and clinical outcomes. | To examine any associations between physiotherapy engagement (duration and frequency) and depression diagnoses on survival and readmission. | Physiotherapy outpatients | Adults aged 60 or over who had surgery for first nonpathological hip fracture. | Age, gender, pre-fracture residence, fracture type, ambulation prior to hip fracture, timing of surgery post-incident, timing of first mobilisation (within or outside target), number of comorbidities, presence of depression, depression diagnosis, days of physiotherapy in the first week post-surgery, duration of physiotherapy in first week post-surgery. | Patient records | Quantitative |
| The Role of the Outpatient Occupational Therapist Treating Patients With Small Burns: A Retrospective Audit of Practice. | 2023 | Journal of burn care & research : official publication of the American Burn Association | O'Reilly S and Strong J and Ziviani J and Brown J and McAuliffe T | M | M | Australia | Occupational therapy | 454 | Yes - the authors cite relevant evidence but outline that there is a gap in terms of understanding the impact of small burns on a clinical service. | Small burns can impact a person's quality of life and still benefit from a range of treatments. Most research is focused on recovery from large burns. | To examine occupational therapy treatments given to patients with small burns in a hospital. | Specialist outpatient’s department | Adults with small burns who required an inpatient admission. | Age, gender, burn injury characteristics (size, location, depth, method of injury, surgical treatment provided, presence of hypertrophic scarring), type of occupational therapy treatment (scar massage, compression therapy, silicone products, taping), clinical outcome measures and scar characteristics over the duration. | Patient records | Quantitative |
| The Timing of Stroke Care Processes and Development of Stroke Associated Pneumonia: A National Registry Cohort Study. | 2022 | Frontiers in neurology | Lobo Chaves MA and Gittins M and Bray B and Vail A and Smith CJ | A | A | England | Mixed- stroke rehabilitation | 413,133 | No | Stroke associated pneumonia (SAP) is a common sequala of stroke and leads to poorer outcomes and increased mortality. Early assessment and management of swallow can reduce the risk of SAP. Other care processes may be associated but there is little research. | To examine stroke care processes associated with risk of SAP. | Multiple pre-hospital sites: hyperacute and acute care | Adults with stroke admitted to hospital | Age, gender, ethnicity, comorbidities, previous stroke or TIA, type of stroke, modified Rankin scale (disability score), stroke severity, dysphagia status, time from stroke onset to hospital admission, thrombolysis status, 'door to needle' time, time from hospital arrival to swallow screen or nurse assessment, time from hospital arrival to stroke specialist doctor, time from hospital arrival to physiotherapist assessment. | Patient records | Quantitative |
| The UK Haemophilia Doctors Organisation triennial audit of UK Comprehensive Care Haemophilia Centres. | 2012 | Haemophilia : the official journal of the World Federation of Hemophilia | Wilde JT | C | n/a | UK | Physiotherapy | Not reported | No | In the UK, patients with haemophilia receive care through comprehensive care centres (CCCs) or smaller haemophilia centres (HCs). A national audit was established. | To examine the quality of haemophilia care in the UK. | Specialist care hubs | Individuals with haemophilia accessing care from the participating CCCs or HCs | Adequacy of patient diagnostic and treatment information, evidence of satisfactory care, availability of professionals at treating centre, plus other audit metrics unrelated to routine data. | Patient records | Quantitative |
| The underuse of couple therapy for depression in Improving Access to Psychological Therapies Services (IAPTS): a service evaluation exploring its effectiveness and discussion of systemic barriers to its implementation. | 2021 | Journal of Family Therapy | Shepherd, Melanie and Butler, Lucy | C | C | England | Psychology/ mental health | 81 | Yes- the authors acknowledge a practice-to-research gap in that services offer a treatment option that does not have a strong evidence base, and situate their research in this context. | Clinical guidance suggests that Couple therapy for depression (CTfD) should be offered as a treatment option for people with depression accessing Access to Psychological Therapies Services (IAPTS). It appears underused though its effectiveness is comparable with other treatment options. | To evaluate the effectiveness of CTfD in the clinical context as well as its accessibility by patients and service factors influencing this. | Community mental health service | Adults with depression referred into IAPTS in the given timeframe and locality. | Age, gender, ethnicity, sexual orientation, Clinical outcome measures (patient health questionnaire (PHQ-9), generalised anxiety disorder assessment (GAD-7), and couples satisfaction index (CSI-32)), assessment conducted, acceptance of therapy, therapy completion rate, number of sessions attended, referral route. | Patient records | Quantitative |
| The Use of Immersive Virtual Reality in Sensory Sessions on a Specialist Dementia Unit: Service Evaluation of Feasibility and Acceptability. | 2023 | Occupational therapy in health care | Clay F and Hunt R and Obiefuna N and Solly JE and Watson E and Wilkinson A and Chohan R and Hatfield C and Fletcher PC and Underwood BR | C | M | England | Occupational therapy | 14 | Partially - the authors acknowledge research in the area but identify that there is no evidence in this clinical setting. | Immersive Virtual Reality (iVR) can be used for people with dementia to enhance relaxation and improve wellbeing. It has not yet been evaluated in a dementia unit setting | To evaluate the feasibility of using iVR in a dementia unit. | Dementia unit in hospital | Adults with dementia on the unit who opted into to iVR | Age, gender, diagnosis, Clinical Dementia Rating Scale score, significant medical conditions, Primary reason for admission, Referred into iVR therapy programme (yes, no) Neuropsychiatric Inventory Scale, SOAP notes, incident reports. | Patient records | Quantitative |
| Treating Posttraumatic Stress Disorder in Female Victims of Trafficking Using Narrative Exposure Therapy: A Retrospective Audit. | 2017 | Frontiers in psychiatry | Robjant K and Roberts J and Katona C | C | M | England | Psychology/ mental health | 10 | Yes- the authors comment that there is evidence about principles of treatment but without testing treatments to apply in clinical practice, which their study aims to address. | Victims of human trafficking experience high levels of post-traumatic stress disorder (PTSD), and have complex and multiple trauma. Narrative exposure therapy (NET) was designed to address PTSD arising from multiple traumas and so may be suitable for this patient group. | To evaluate the use of NET for victims of human trafficking and PTSD. | Mental health services (charitable organisation) | Women with PTSD following human trafficking experience | Immigration status, country of origin, age, experience of traumatic events, use of interpreters, Posttraumatic Diagnostic Scale (PDS) scores, Clinical Outcomes in Routine Evaluation (CORE) scores (pre and post therapy and follow up), number of sessions. | Patient records | Quantitative |
| Treatment characteristics among patients with binge-eating disorder: an electronic health records analysis. | 2023 | Postgraduate medicine | Spalding WM and Bertoia ML and Bulik CM and Seeger JD | Cor | Mixed | USA | Psychology/ mental health | 384 | Yes- the authors cite that there is absence of evidence from a real-world setting and situate their study in this. | There are multiple treatment options for people with binge-eating disorder supported by research, but little is known about what is really used in practice. | To describe routine clinical treatments for people with binge-eating disorder. | Mental health providers contributing data | Adults with binge eating disorder accessing participating services at least once. | Age, gender, ethnicity, Diagnosis (confirmed or probable), comorbidities, BMI, weight category, geographic region, year accessing services, smoking status, physical activity level, type of treatment, medicinal treatments. | Patient records | Quantitative |
| Treatment of low back pain: monitoring clinical practice through audit. | 2005 | Physiotherapy | V, Sparkes | C | n/a | England | Physiotherapy | 130 | No | Back pain is a major and very common cause of disability and can be treated by physiotherapists. There is mixed evidence regarding the effectiveness of exercise programmes for lower back pain. | To evaluate routine practice through two audits of routine care to established standards. | Outpatient physiotherapy service | Adults with lower back pain referred to physiotherapy and attended at least one appointment. | Age, Adherence to care standards in relation to: Nature of back pain (acute or chronic), time assessed following referral, provision of advice to keep active, diagnostic triage, onward referrals, advice given re. bed rest, manipulation given for symptoms, assessment red flags, psychosocial yellow flags, association with violent trauma, pain site, nature of pain (constant, progressive, non-mechanical), medical history, weight loss and general health, neurology symptoms, restriction of lumbar flexion, presence of structural deformity | Patient records | Quantitative |
| Treatment patterns of multiple sclerosis patients: a comparison of veterans and non-veterans using the NARCOMS registry. | 2005 | Multiple sclerosis (Houndmills, Basingstoke, England) | Lo AC and Hadjimichael O and Vollmer TL | M | M | USA | Physiotherapy | 20963 | Yes- the authors highlight shortfalls with clinical trials and the benefits of large real-world databases and situate their study in this context. | The therapy/treatment for Multiple sclerosis (MS) therapy is variable as is the course of the disease. Looking at disease and treatment on a large scale is useful for identifying patterns. The North American Research Committee on Multiple Sclerosis (NARCOMS) Database may be useful for this. | To explore characteristics and treatment patterns of MS in veterans using the NARCOMS database. | Mixed- medical centres | Veterans with MS receiving care from participating centres | Age, gender, duration of disease, MS diagnosis, MS type, Veteran status, Patient-Determined Disease Steps (PDDS) scores (disability scale, handicap scale, quality of life scale) healthcare services used, insurance type, medical history, therapies, and treatments received, use of medications for specific symptoms. | Patient records | Quantitative |
| Understanding differences in mental health service use by men: an intersectional analysis of routine data. | 2022 | Social psychiatry and psychiatric epidemiology | Smyth N and Buckman JEJ and Naqvi SA and Aguirre E and Cardoso A and Pilling S and Saunders R | A | M | England | Psychology/ mental health | 9904 | No | There is emerging evidence around social characteristics and their association with mental health service use. Understanding this is useful to identify subgroups who may be at risk of discontinuing treatment and obtaining poorer outcomes. There is an absence of research utilising a quantitative approach to this. | To explore the association between social determinants and use of treatment services and identify potential at-risk groups | Community mental health service | People who identify as male referred into the service and had received an assessment from the participating sites. | Age, gender, ethnicity, sexual orientation, religious affiliation, employment status, neighbourhood deprivation, assessment outcome group (entered, disengaged, service deemed unsuitable), treatment phase group (completed, disengaged, referred elsewhere), Patient Health Questionnaire 9-item scores and the Generalised Anxiety Disorder-7 scores. | Patient records | Quantitative |
| Using a Whole Person Approach to Support People With Cancer: A Longitudinal, Mixed-Methods Service Evaluation. | 2016 | Integrative cancer therapies | Polley MJ and Jolliffe R and Boxell E and Zollman C and Jackson S and Seers H | A | M | England | Psychology/ mental health | 135 | Partially- the authors comment that service evaluations are useful to understand complex health interventions prior to a controlled trial. | People with cancer or living beyond cancer have a poorer quality of life and overall health. Holistic care is helpful to support their varied psychological and emotional and health-related quality of life needs. A new course 'Living Well with the Impact of Cancer' was created to address this. | To evaluate the effectiveness of the Living Well with the Impact of Cancer (LWC) in the short and long-term. | Charity | Adults living with cancer | Age, gender, residential (yes or no), type of cancer, disease stage, mortality at follow up, Measure Yourself Concerns and Wellbeing (MYCaW) scores pre and post, Functional Assessment of Chronic Illness Therapy Extended Spiritual questionnaire scores pre and post, participant satisfaction data. | Patient records | Quantitative |
| Using transprofessional care in the emergency department to reduce patient admissions: A retrospective audit of medical histories. | 2016 | Journal of interprofessional care | Morphet J and Griffiths DL and Crawford K and Williams A and Jones T and Berry B and Innes K | A | M | Australia | Mixed- physiotherapy and occupational therapy | 150 | No | Hospital emergency department waiting times are often beyond targets. A transprofessional care model within an emergency department was introduced to address this issue. | To evaluate the outcomes from the new transprofessional care model in terms of patient admission rates, length or stay and patient re-admittance. | Emergency department | Patients presenting to the emergency department referred to the care pathway plus matched sample not referred. | Age, gender, time, month and day of admittance, presenting complaint, triage category, end diagnosis/disposition, past medical history, social support, living arrangements, interventions given, investigations undertaken, length of stay in emergency department, profession referring into the transprofessional team, discharge details. | Patient records | Quantitative |
| Utilising a non-surgical intervention in the knee osteoarthritis care pathway: a 6-year retrospective audit on NHS patients. | 2023 | Therapeutic advances in musculoskeletal disease | Benn R and Rawson L and Phillips A | C | C | England | Physiotherapy | 571 | No | Knee osteoarthritis is a substantial cause of disability and demand on surgery. Non-surgical alternative interventions are useful. A home-based non-surgical treatment option is biomechanical intervention. | To explore clinical outcomes and onward referral rates of patients with knee osteoarthritis who have used the biomechanical intervention. | Musculoskeletal clinic | Adults with knee osteoarthritis considered suitable for this treatment. | Age, gender, days of treatment, Onward referral to secondary care consultation in subsequent years post-treatment (service type and sub-service), Western Ontario and McMaster Universities Osteoarthritis Index (WOMAC) questionnaire scores, OKS scores, OptoGait system assessment scores. | Patient records | Quantitative |
| Variability in patient characteristics and service provision of interdisciplinary pain rehabilitation: A study usingÂ the Swedish national quality registry for pain rehabilitation. | 2020 | Journal of rehabilitation medicine | Fischer MR and Schults ML and StÃ¥lnacke BM and Ekholm J and Persson EB and LÃ¶fgren M | M | M | Sweden | Mixed- pain rehabilitation | 3511 | Yes- the authors comment on the evidence base but identify there is scarce research focused on real-life settings and how using routine data via a registry can provide valuable knowledge to address this gap. | Multi-disciplinary pain rehabilitation may lead to favourable outcomes for people experiencing chronic pain. Research reports lack detail on what the rehabilitation involves. | To evaluate registry data to describe routine interdisciplinary pain rehabilitation and explore differences in the type of patients presenting to different type of rehabilitation settings. | Rehabilitation clinics via a registry | Adults with chronic pain referred to rehabilitation unit | Pain severity, native country, treating clinic, pain location/s, anxiety and depression scores (HADS), EQ5D index, Health status (Thermometer), SF-36 Physical function and SF-36 Vitality scores, number of days of rehabilitation, hours per day, rehabilitation intensity. | Patient records | Quantitative |
| Video versus direct laryngoscopy by specialist paramedics in New South Wales: Preliminary results from a new airway registry. | 2022 | Emergency Medicine Australasia | Nichols, Martin and Fouche, Pieter F and Bendall, Jason C | C | C | Australia | Paramedical science | 322 | No | Video laryngoscopy may provide better visualisation of the glottis to support endotracheal intubation success by paramedics, compared with direct laryngoscopy. Data is routinely collected about airway management. | To compare video laryngoscopy with direct laryngoscopy to assist endotracheal intubation by specialist paramedics in terms of success rate | Emergency services | People who required endotracheal intubation by paramedics | Age, gender, condition, Cormack-Lehane Grading at first intubation attempt, presumed cause of cardiac arrest, primary indication for advanced airway use, medications used prior to airway management, relevant patient factors (obesity, pregnancy, beard, dentures, piercing, deformity, neck movement restricted, trismus) | Patient records via Registry | Quantitative |
| Voice therapy outcomes in vocal fold nodules: a retrospective audit. | 2001 | International journal of language & communication disorders | McCrory E | A | n/a | Northern Ireland | Speech and language therapy | 26 | No | Voice therapy can improve outcomes from voice disorders but there is only a small body of research that has investigated the effectiveness of voice therapy. | To evaluate routine management approaches for people with vocal fold nodules and how to compares to the best practice standards. | Outpatients voice clinic | Adults with vocal fold nodules referred to the clinic | Age, gender, referral source, therapy components, laryngoscopy findings, auditory perceptual voice quality ratings, fundamental frequency scores, patient voice quality ratings, number of therapy sessions attended. | Patient records | Quantitative |
| Was the impact of COVID-19 on a spinal triage service as significant as expected? A retrospective service evaluation: Results and evaluation. | 2022 | Musculoskeletal care | Wood L and Eveleigh C and Dixon M and Dunstan E and Salem K | M | C | England | Physiotherapy | 407 | No | Advanced practitioner physiotherapists have become embedded in services to mitigate long waiting lists and provide non-surgical treatment for people with spinal pain. COVID-19 disrupted these kinds of services. The effect on the given site of spinal triage service is unknown. | To compare pre-COVID-19 and post-COVID-19 referral rates and how care was received. | Secondary care spinal unit | Adults with spinal pain referred to the service | Year of referral, new referral status, mode of appointment (telehealth or routine), wait time for appointment, number of follow up appointments, wait time for follow up appointment, appointment attendance, discharge at first attendance, MRI requested, Injection requested, hours of clinical time, virtual MDT appointment, virtual MDT appointment outcome, face-to-face MDT appointment, face-to-face MDT appointment outcome. | Patient records | Quantitative |
| Weekly variation in health-care quality by day and time of admission: a nationwide, registry-based, prospective cohort study of acute stroke care. | 2016 | Lancet (London, England) | Bray BD and Cloud GC and James MA and Hemingway H and Paley L and Stewart K and Tyrrell PJ and Wolfe CD and Rudd AG | A | M | England | Mixed- stroke rehabilitation | 74307 | No | Quality of stroke care is known to vary by day of hospital admission. Given that outcomes following stroke are also time-dependent, it is possible that time of admission during the day may also have an effect. | To examine whether there is an effect of time of day of admission for stroke and outcomes. | Acute hospitals | Adults with stroke admitted to hospital | Age, gender, place of stroke onset (in or out of hospital), stroke type, vascular comorbidity (atrial fibrillation, heart failure, diabetes mellitus, previous stroke or transient ischaemic attack, hypertension), pre-stroke functional level (as measured by the modified Rankin Scale), time from stroke onset to admission, stroke severity (National Institutes of Health Stroke Scale score [NIHSS] or level of consciousness on admission, time of admission (time category), survival at 30 days, quality of care received (in line with standards). | Patient records | Quantitative |
| What is "usual care" in the rehabilitation of upper limb sensory loss after stroke? Results from a national audit and knowledge translation study. | 2022 | Disability and rehabilitation | Cahill LS and Lannin NA and Purvis T and Cadilhac DA and Mak-Yuen Y and O'Connor DA and Carey LM | A | M | Australia | Mixed- physiotherapy and occupational therapy | 24996 | Yes- the authors specifically cite research to practice gap and the use of a knowledge translation study to address this, which forms part of the audit they report. | Many people post stroke experience somatosensory symptoms, but traditional rehabilitation research has overlooked this. Clinical trials use 'usual practice' as a comparator though it is unknown what usual practice is in somatosensory rehabilitation and more evidence is needed on this. | To characterise usual practice in somatosensory rehabilitation for people post-stroke. | Acute and rehabilitation hospitals | Adults with stroke admitted to hospital | Age, gender, pre-morbid function (modified Rankin Scale), co-morbidities including previous stroke (acute only), stroke type, stroke severity, cognitive impairment on admission (rehabilitation only), current function (Functional Independence Measure) (rehabilitation only), and use of hospital protocols for sensory impairment (acute only) plus questionnaire data. | Patient records | Quantitative (an additional component not using routine data adopted a qualitative approach) |
| What predicts falls in Parkinson disease?: Observations from the Parkinson's Foundation registry. | 2018 | Neurology. Clinical practice | Parashos SA and Bloem BR and Browner NM and Giladi N and Gurevich T and Hausdorff JM and He Y and Lyons KE and Mari Z and Morgan JC and Post B and Schmidt PN and Wielinski CL | M | M | USA | Mixed- falls management | 3795 | No | People with Parkinson's disease are at risk of falls, subsequently requiring complex, burdensome medical care. Falls prevention strategies may be useful though identifying those at risk of falling is helpful for targeting falls prevention. | To identify characteristics of people with Parkinson's disease who are at risk of falls. | Parkinson’s clinics | Adults with Parkinson's attending NPF Centers of Excellence | Age, gender, race, living arrangements, support from partner/carer, study site, disease duration, confidence in diagnosis, presence of rest tremor, presence of motor fluctuations history of falls over the preceding 3 months, comorbidities, treatment with levodopa, dopamine agonists, monoamine oxidase type B inhibitors, catechol-O-methyl transferase inhibitors, amantadine, anticholinergics, antidepressants, antipsychotics, and cognitive enhancers, history of DBS for PD, hospitalizations in the preceding year, physical, occupational, and speech therapy, exercise, social or mental health services, Parkinson's Disease Questionnaire (PDQ-39) scores, Timed Up-and-Go (TUG) scores, modified Multidimensional Caregiver Strain Index (MCSI) scores, immediate and delayed recall of 5 words (from version 1 of the English language Montreal Cognitive Assessment), semantic fluency (number of animals recited in 1 minute), new addition or discontinuation of any of the above classes of medications, new referrals for any of the above mentioned treatment modalities. Changes in medical history between visits: changes in comorbidities, hospitalizations, or living situation between baseline and follow-up visits. | Patient records via Registry | Quantitative |
| Who benefits from multimodal rehabilitation - an exploration of pain, psychological distress, and life impacts in over 35,000 chronic pain patients identified in the Swedish Quality Registry for Pain Rehabilitation. | 2019 | Journal of pain research | Gerdle B and Ã…kerblom S and Brodda Jansen G and Enthoven P and Ernberg M and Dong HJ and StÃ¥lnacke BM and Ã„ng BO and Boersma K | A | M | Sweden | Mixed- pain rehabilitation | 39916 | No | Psychological symptoms of anxiety and depression are related to pain and recovery from pain, but the relationship is unclear and it is unknown how multi-modal rehabilitation impacts this. | To evaluate pain and psychological symptoms in people receiving pain rehabilitation to explore their relationship, and to identify how this impacts rehabilitation outcomes | Rehabilitation clinics via a registry | Adults with chronic pain attending participating clinics | Age, gender, educational level, country of birth, pain intensity, pain severity, areas of pain, psychological distress (HADS), pain interference scales | Patient records via Registry | Quantitative |

***Note: Lead author affiliation: A= academic affiliation, C=clinical affiliation, M=multiple and diverse affiliations***
